# CD64-Targeted Polymer-Drug Conjugates Exploit Cathepsin K‑Dependent Payload Release for Selective Elimination of Immunosuppressive Macrophages

**DOI:** 10.1021/acs.molpharmaceut.5c01931

**Published:** 2026-04-21

**Authors:** Dominik Musil, Markéta Krhutová, Kristýna Blažková, Anežka Kramná, Andrea Brázdová, Barbora Výmolová, Magdalena Houdová Megová, Martin Hadzima, Robin Kryštůfek, Vladimír Šubr, Libor Kostka, Tomáš Etrych, Tereza Ormsby, Pavel Šácha, Jakub Abramson, Jan Konvalinka

**Affiliations:** † Institute of Organic Chemistry and Biochemistry of the Czech Academy of Sciences, Prague 6 160 00, Czech Republic; ‡ Department of Genetics and Microbiology, Faculty of Science, 69729 Charles University, Prague 2 128 00, Czech Republic; § Department of Cell Biology, Faculty of Science, Charles University, Prague 2 128 00, Czech Republic; ∥ Institute of Biochemistry and Experimental Oncology, First Faculty of Medicine, Charles University, Prague 2 128 00, Czech Republic; ⊥ Institute of Macromolecular Chemistry of the Czech Academy of Sciences, Prague 6 162 00, Czech Republic; # Department of Immunology and Regenerative Biology, Weizmann Institute of Science, Rehovot 7610001, Israel; ¶ Department of Biochemistry, Faculty of Science, Charles University, Prague 2 12800, Czech Republic

**Keywords:** CD64, targeted polymer-drug conjugates (TPDCs), monocyte-derived macrophages (MDMs), tumor-associated macrophages (TAMs), immunotherapy

## Abstract

Selective depletion of immunosuppressive macrophages in the tumor microenvironment is a promising strategy in cancer therapy. CD64 is broadly expressed on myeloid cells, including both pro-inflammatory M1-like and immunosuppressive M2-like macrophages that resemble tumor-associated macrophages (TAMs), and thus represents an attractive entry receptor for targeted payload delivery. We developed HPMA-based CD64-targeted polymer–drug conjugates (CD64-TPDCs) that combine multivalent receptor engagement with enzyme-responsive payload release. These copolymers are decorated with the CD64-binding cyclic peptide cp33 and carry the cytotoxic payload mertansine (DM1) bound via cathepsin-cleavable peptide linkers. Multivalent cp33 presentation on the polymer markedly increased the apparent affinity for human CD64, resulting in subnanomolar binding and selective recognition of CD64-expressing cells, significantly improving the binding potency of monovalent cp33 peptide. In polarized M2-like human monocyte-derived macrophages (MDMs), we showed that cytotoxic Gly-Phe-Leu-Gly-DM1 CD64-TPDCs selectively induced apoptosis. In contrast, M1-like MDMs were largely spared despite expressing higher levels of CD64. In M2-like MDMs, CD64-TPDCs rapidly accumulated in lysosomes, whereas in M1-like cells, they remained largely confined to endosomes. To elucidate the basis of this selectivity, we profiled expression of cathepsins in polarized MDMs. We found that M2-like MDMs display substantially higher levels of cathepsin K, establishing a model in which cathepsin K is the major protease responsible for Gly-Phe-Leu-Gly linker cleavage and DM1 release in M2-like macrophages. These findings demonstrate that CD64-TPDCs can be engineered to exploit subset-specific trafficking and cathepsin K-dependent linker cleavage for the selective elimination of M2-like macrophages. This work provides a generalizable design principle for stimuli-responsive PDCs that may actively target immunosuppressive myeloid cells in tumors.

## Graphical abstract

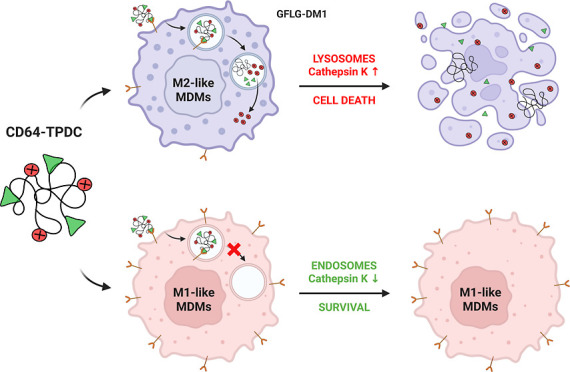

## 1 Introduction

Macrophages are key immune effector cells involved in host defense, inflammation, cytokine production, phagocytosis, and tissue remodeling.^1^ They exhibit remarkable phenotypic plasticity and can be polarized into classically activated (M1) and alternatively activated (M2) states in response to microenvironmental signals.^2^ Although these functionally distinct subtypes exist along a continuum, the M1/M2 paradigm remains a useful concept for understanding macrophage plasticity in health and disease.

Under pathological conditions, macrophages can acquire immunosuppressive functions. Within the tumor microenvironment (TME), tumor-associated macrophages (TAMs) contribute to uncontrolled tumor growth.^3–9^ TAMs, which often display an M2-like phenotype, secrete pro-angiogenic factors and immunosuppressive cytokines such as IL-10 and TGF-β, that inhibit the activation and effector functions of T cells.^10^ Moreover, TAM-secreted chemokines such as CCL22 facilitate the recruitment of regulatory T cells (Tregs) to the tumor site.^11^ The coordinated immunosuppressive activity of TAMs and Tregs further suppresses cytotoxic T lymphocyte (CTL) responses and impairs antitumor immunity, thereby promoting tumor progression, metastasis, and resistance to immunotherapeutic interventions.

Consequently, there is considerable interest in therapeutic strategies that target or modulate macrophage activity. Strategies under investigation include TAMs depletion or repolarization, CAR macrophages,^12^ and blocking circulating monocytes to avoid their recruitment. For example, small-molecule CSF1R inhibitors Pexidartinib or CSF1R monoclonal antibody (mAb) Emactuzumab,^13^ are clinically used to modulate TAM phenotypes or block downstream cytokine signaling.^14,15^

Direct targeting of macrophages through specific surface immunoreceptors represents an additional strategy for myeloid-cell-targeting therapies.^16^ Among these receptors, the high-affinity IgG FcγRI receptor CD64 ^17^ has emerged as an attractive target due to its abundant expression on monocytes, acute myeloid leukemia cells, and tissue-resident macrophages, including TAMs.^18,19^ Elevated CD64 expression on TAMs has been associated with poor clinical prognosis in metastatic melanoma and glioblastoma,^20,21^ indicating a potential role in tumor progression and aggressiveness. CD64-targeted therapies, including CD64-based CAR-T cells,^22^ synthetic antibody mimics,^23^ and various antibody-drug conjugates (ADCs) and bispecific engagers,^24–32^ are being explored in oncology and autoimmune disease.^24,33^ Notably, some anti-CD64 ADCs have shown limited cytotoxicity toward macrophages^29^ or preferentially eliminated pro-inflammatory M1 macrophages while sparing M2 macrophages,^32^ highlighting the importance of context-dependent targeting strategies.

To develop an alternative to antibody-based CD64 immunotherapy, we designed cytotoxic CD64-TPDCs, a class of modular polymer-drug conjugates comprising of synthetic *N*-(2-hydroxypropyl)-methacrylamide (HPMA) copolymers serving as a biocompatible and fully soluble polymer carrier.^34,35^ The TPDCs are decorated with a small cyclic peptide binder cp33 ^36^ and carry potent cytotoxic payloads such as monomethyl auristatin E (MMAE) or mertansine (DM1) connected to the polymer carrier backbone via cathepsin-cleavable peptide linkers. The HPMA copolymer scaffold enables simultaneous conjugation of multiple ligands and functional moieties and allows control of copolymer size and structure, which improves pharmacokinetic and pharmacodynamic properties.^37,38^ Notably, HPMA copolymers have been shown to passively accumulate in tumors via the enhanced permeability and retention (EPR) effect, supporting their use as highly efficient carriers in macromolecular anticancer therapies.^38,39^

In this study, we present the selective cytotoxic activity of CD64-TPDCs *in vitro* toward M2-like MDMs while sparing M1-like cells. To elucidate the mechanism behind this macrophage subset-specific selectivity, we investigated internalization and trafficking of CD64-TPDCs, their processing within cellular compartments, and cathepsin-mediated cleavage of the peptide linkers, with particular emphasis on the role of cathepsin K. Our results provide mechanistic insight into macrophage subset targeting and demonstrate that CD64-directed, enzyme-responsive TPDCs can be engineered to preferentially eliminate pro-tumorigenic M2-like macrophages to boost myeloid cancer immunotherapy toward TAMs.

## 2 Materials and Methods

### 2.1 Synthesis of Monomers and of the Chain Transfer Agent

Monomers HPMA and 3-(3-methacrylamidopropanoyl)­thiazolidine-2-thione (Ma-β-Ala-TT) were synthesized as previously described.^40,41^ The chain transfer agents S-2-cyano-2-propyl S′-ethyl trithiocarbonate (CTA) and *N*-(3-azidopropyl)-4-cyano-4-ethylsulfanylcarbothioylsulfanyl-pentanamide (CTA-N_3_) were also synthesized as previously described.^42,43^

### 2.2 Synthesis of Polymer Precursors

HPMA-based copolymer precursors P1, P2, and P3 (poly­(HPMA-*co*-Ma-β-Ala-TT)) were prepared by reversible addition–fragmentation chain transfer (RAFT) copolymerization of HPMA with Ma-β-Ala-TT. Briefly, the example for the synthesis of precursor P2, 1.2 g of HPMA (88 % mol), 0.295 g of Ma-β-Ala-TT (12%mol) were dissolved in 2.0 mL *N*,*N*-dimethylacetamide (DMA). To this solution, 3.0 mg of the chain transfer agent CTA, 2.26 mg of the low-temperature initiator 2,2′-azobis­(4-methoxy-2,4-dimethylvaleronitrile) (V-70), and 11.5 mL of *tert*-butanol were added. The polymerization mixture was introduced into a polymerization ampule, bubbled with argon for 10 min, and then sealed. Polymerization was carried out at 40 °C for 18 h. The polymer precursor was isolated by precipitation in a mixture of acetone/diethyl ether (3:1), filtered off, washed with acetone and diethyl ether and dried under vacuum. The terminating trithiocarbonate group was removed as described by Perrier et al.^44^ Precursors P1 and P3 were prepared by the same procedure.

Semitelechelic HPMA-based copolymer precursor P4 (poly­(HPMA-*co*-Ma-β-Ala-TT)-N_3_) was prepared by RAFT copolymerization. 1.0 g of HPMA (90 % mol), 0.20 g of Ma-β-Ala-TT (10 % mol) were dissolved in 1.0 mL DMA. To this solution, 4.47 mg of the chain transfer agent CTA-N_3_, 1.99 mg of the low-temperature initiator V-70, and 8.2 mL of *tert*-butanol were added. The polymerization mixture was introduced into a polymerization ampule, bubbled with argon for 10 min, and then sealed. Polymerization was carried out at 40 °C for 18 h. The polymer precursor was isolated by precipitation in a mixture of acetone/diethyl ether (3:1), filtered off, washed with acetone and diethyl ether and dried under vacuum. The terminating trithiocarbonate group was removed.^44^ The characterization of polymer precursors is summarized in Table S1.

### 2.3 Synthesis of TPDCs

Copolymer conjugates **fC1–fC3** and **C4–C12** were prepared, isolated, and purified using the same procedure as described elsewhere.^38^ Briefly, the conjugates were synthesized through a polymer-analogous reaction involving the copolymer precursor with thiazoline-2-thione (TT) reactive groups and amino-terminated low-molecular-weight compounds. The compounds included an anti-CD64 ligand (cp33 peptide), a fluorescent dye (ATTO488, ATTO647N), biotin, and linkers with MMAE and DM1 derivatives (Figure S1). The reaction was carried out in DMSO in the presence of *N*,*N*-diisopropylethylamine (DIPEA) at RT for 4 h. Residual TT reactive groups were removed by the addition of 2 μL of 1-amino-propan-2-ol (2.59 × 10^–2^ mmol), and the reaction was stirred for 10 min. The TPDCs-containing reaction mixture was diluted with 1 mL of methanol, and the TPDCs were separated on a 1.5 × 18 cm chromatography column with Sephadex LH-20 in methanol, equipped with the UV–vis detector Azura UVD 2.1S (Knauer). Methanol was evaporated, the TPDCs were dissolved in Milli-Q water and purified on a PD10 column and lyophilized. Detailed preparation of each individual conjugate is described in Supporting Information.

### 2.4 Characterization of Polymer Precursors and TPDCs

The weight-average molecular weights (*M*_w_), number-average molecular weights (*M*_n_), and dispersity (*D̵*) of the polymer precursors P1, P2, P3 and P4 as well as conjugates **fC1**–**fC3** and **C4**–**C12** were determined using a Shimadzu HPLC system equipped with a UV detector, an Optilab rEX differential refractometer, a DAWN 8 multiangle light-scattering detector (Waters/Wyatt Technology), and a TSKgel G4000SWXL size-exclusion chromatography column. *M*_w_, *M*_n_ and *D̵* were calculated using the Astra V software with a refractive index increment d*n*/d*c* = 0.167 mL g^–1^ utilized for the calculation. These experiments were carried out in a solvent containing 300 mM sodium acetate buffer (pH 6.5) and methanol (20%/80% v/v), with a flow rate of 0.5 mL min^–1^. *M*_w_, *M*_n_ and *D̵* were calculated using the Astra 8.1.2 software with a refractive index increment d*n*/d*c* = 0.167 mL g^–1^ utilized for the calculation. The estimated molecular weight *M*_w_’ was calculated as the sum of the molecular weight of polymer precursor and bound ligands.

The content of TT reactive groups in the polymer precursors was determined spectrophotometrically (ε_302 nm_ = 10,600 L mol^–1^ cm^–1^, methanol). The content of ATTO488 in the conjugate **fC1** (ε_646 nm_ = 90,000 L mol^–1^ cm^–1^, water), the ATTO647N in the TPDCs **fC2** and **fC3** (ε_646 nm_ = 150,000 L mol^–1^ cm^–1^, water), were determined using spectrophotometer Specord 205.

The content of NH_2_–PEG_11_-biotin in **fC2** and **fC3** was determined using the HABA/Avidin Reagent kit (Merck) for spectrophotometric determination at 500 nm according to the manufacturer’s instructions (Sigma, H 2135); the results were corrected for the effect of ATTO647N absorbance at 646 nm. The content of targeting ligands, such as cp33 peptide, NH_2_–Val-Cit-PAB-MMAE, NH_2_-Gly-Val-Cit-Gly-DM1, NH_2_-Gly-Phe-Leu-Gly-DM1 and NH_2_-TrisNTA in the TPDCs was determined by amino acid analysis with precolumn 2,3-naphthalenedicarboxaldehyde (NDA)/NaCN derivatization on a Shimadzu HPLC system with an RF-20A fluorescence detector (Ex = 229 nm/Em = 490 nm) using a reverse-phase Chromolith RP18e column (100 × 4.6 mm). A gradient of solvent sodium acetate buffer in methanol was used with a flow rate of 1 mL min^–1^. Prior to analysis, the sample was hydrolyzed with 6 M HCl at 115 °C for 16 h. The hydrolysate was dried in a vacuum and dissolved in water. The characterization and content of various moieties bound to the TPDCs’ HPMA polymer scaffold are summarized in Table S2.

### 2.5 List of CD64-TPDCs

Twelve different types of CD64-TPDCs, encompassing both fluorescent and cytotoxic variants, were synthesized. The fluorescent TPDCs include the specific **fC1** and **fC2** variants, which contain the targeting ligand cp33 and fluorophores (ATTO488 and ATTO647N), and the control fluorescent conjugate **fC3**, which lacks cp33 and has only the fluorophore (ATTO647N). The cytotoxic variants of CD64-TPDCs **C4**, **C6**, and **C8** incorporate cp33 targeting ligands and cytotoxic payloads (MMAE or DM1) conjugated to the HPMA copolymer backbone via cathepsin-cleavable linkers (Val-Cit-PAB, Gly-Val-Cit-Gly or Gly-Phe-Leu-Gly). Control cytotoxic PDCs, **C5**, **C7**, and **C9** contain the cytotoxic payload without cp33. Additional controls include **C10**, a polymeric conjugate with cp33 only, and **C11**, the HPMA copolymer without any ligands. Lastly, compound **C12** contains biotin and TrisNTA ligands.

### 2.6 Human Cell Lines

HEK cell lines were maintained in IMDM complete medium (IMDM medium supplemented with 4 mM l-glutamine, 10% (v/v) fetal bovine serum (FBS) and the antibiotics puromycin dihydrochloride (5 μg/mL) and Geneticin (400 μg/mL), if added). All cell lines were grown at 37 °C in a humidified 5% CO_2_ atmosphere.

### 2.7 Preparation of HEK 293 Cells with Inducible CD64 Expression

HEK 293 cells were stably transfected with the pTetOff Advanced vector using the FuGENE HD transfection reagent following the manufacturer’s protocol and grown in the presence of Geneticin (400 μg/mL) for selection. Stable transfectant colonies were selected and grown to confluence. The best clone, HEK 293-pTetOff-A2 (HEK A2 cells^45^), was selected for further stable transfection. HEK A2 cells were stably cotransfected with the pTRETight-CD64 plasmid and the pPUR vector using the FuGENE HD transfection reagent following the manufacturer’s protocol. Stable transfectant colonies, HEK 293-pTetOff-A2 CD64-expressing cells (HEK A2 CD64 cells), were grown to confluence in the presence or absence of 100 nM doxycycline hyclate in IMDM complete medium containing puromycin dihydrochloride (0.5 μg/mL) and Geneticin (400 μg/mL). The expression level and its regulation by doxycycline hyclate addition were analyzed using flow cytometry.

### 2.8 Production and Purification of CD64 Protein

The extracellular part of hCD64 (residues 16–289) was expressed as a recombinant protein with a TEV protease cleavage site, Strep-tag, and histidine tag. For expression, *Drosophila* S2 cells were transfected with the pMT-BiP_CD64aEC-StrepII-His6 plasmid, and expression was induced with 1 mM CuSO_4_. Supernatant was collected, and recombinant hCD64 was purified using HIS-Select Nickel Affinity Gel. Following size-exclusion chromatography, selected fractions were pooled and concentrated using an Amicon ultracentrifugal filter with a 10 kDa MWCO.

### 2.9 Surface Plasmon Resonance

Gold-coated glass-based SPR chips were functionalized with alkanethiol molecules carrying terminal carboxylic groups by a mixture of 200 μM HS-(CH_2_)_11_-(CH_2_CH_2_O)_6_–OCH_2_–COOH and HS­(CH_2_)_11_-(CH_2_CH_2_O)_4_–OH (3/7 ratio of COOH/OH) for 60 min at 37 °C. The chips were then washed with deionized H_2_O and UV ethanol, dried, and placed into the Plasmon-IV SPR (Institute of Photonics and Electronics, Czech Academy of Sciences). Carboxylic groups were activated with *N*-hydroxysuccinimide/(1-ethyl-3-(3-(dimethylamino)­propyl)­carboiimide (NHS/EDC), providing the means for attachment of 1 mg/mL neutravidin in 1 mM sodium acetate buffer. To prevent nonspecific binding and block unreacted carboxyl groups, the chips were treated with high-salt PBS (0.33 M NaCl, 2.5 mM KCl, 8.1 mM Na_2_HPO_4_, 1.5 mM KH_2_PO_4_, pH 7.4) and 1 M ethanolamine. Subsequently, 100 nM of Ni^2+^-charged HPMA copolymer **C12** in TBS (20 mM Tris, 150 mM NaCl, pH 7.4) containing TrisNTA and biotin ligands, or TBS as a negative control, was bound to the neutravidin layer. Then, 400 nM of His-tagged hCD64 protein was bound (with one channel left as a negative control with TBS). Finally, the chip was treated with various concentrations of **fC1**–**C11**. In the last step, TBS was run through all channels to observe the dissociation of the TPDCs. K_d_ values were analyzed using SPR UP and TraceDrawer software.

### 2.10 DNA-Linked Inhibitor Antibody Assay (DIANA)

DIANA was optimized for the detection of CD64-TPDCs based on protocols from Navrátil et al.^46^ Briefly, 10 μL of 10 ng/μL neutravidin in TBS was immobilized in a FrameStar 96 multiwell plate, centrifuged at 1000*g* (Allegra X-15R, Beckman Coulter) for 5 min and incubated for 60 min at room temperature (RT). Plates were covered with adhesive seals during all incubations to prevent evaporation. Next, 150 μL of 10 × diluted SuperBlock solution in TBS was added to the wells, followed by overnight incubation at RT in the dark. Wells were then washed three times with TBST (0.05% Tween20 in TBS). Subsequently, 10 μL of 100 nM of

Ni^2+^-charged **C12** in TBST was added to each well, incubated for 60 min at RT in the dark, and washed three times with TBST. Next, 10 μL of a solution containing 40 ng of CD64 protein in TBST was added to each well, followed by centrifugation and incubation at RT for 60 min in the dark, and another three washes with TBST. Then, 10 μL of test compound at various concentrations dissolved in TBST’ (0.1% Tween20 in TBS) with 0.01% casein blocker was added to each well, centrifuged, and incubated for 30 min at RT in the dark. Following this, 500 pM CD64-probe in TBST’ with 0.01% casein blocker was added to the wells. The CD64 monovalent probe was prepared by reacting a DNA oligonucleotide with the sequence CCT GCC AGT TGA GCA TTT TTA TCT GCC ACC TTC TCC ACC AGA CAA AAG CTG GAA A, terminally modified with a 6-amino-2-(hydroxymethyl)­hexyl group, with a 20-fold excess of the cp33 ligand at RT overnight, followed by washing using an Amicon ultracentrifugal filter with a 30 kDa MWCO. After a 60 min incubation at RT, the plate was washed three times with TBST. Finally, 5 μL of a qPCR reaction mixture containing LightCycler 480 SYBR Green I Master by Roche and forward and reverse primers (CCA GCT TTT GTC TGG TGG AG and CCT GCC AGT TGA GCA TTT TT; final concentration 1 μM each), and fluorescent hydrolysis probe #87 from the Roche Universal Probe Library (LNA octamer sequence CTG CCA CC, final concentration 100 nmol/L) was added to each well. The plate was sealed with adhesive optical film, and the detection probe quantified by qPCR: 3 min at 95 °C; then 45 cycles of 10 s at 95 °C, 30 s at 66 °C, and 30 s at 72 °C; and 2 min at 37 °C. Fluorescence was measured using a Light Cycler 480 II qPCR instrument (Roche) with excitation and emission filters adjusted to 465 and 510 nm, respectively. Threshold cycles (Cq) were obtained from the measured fluorescence curves using the second derivative maxima method in the Light Cycler 480 II Software.

### 2.11 *In Vitro* Cell Cytotoxic Assays

HEK cells were seeded at 1 × 10^3^ cells/well in a 384-well plate. Cells were suspended in 20 μL of IMDM complete medium without phenol red. Subsequently, 5 μL of TPDCs diluted in PBS in a dilution series were added to the cells in the appropriate wells, reaching a final volume of 25 μL. To avoid evaporation, peripheral wells were filled with 25 μL of PBS. The samples in a 384-well plate were incubated at 37 °C in a humidified 5% CO_2_ atmosphere without further washing. Following the incubation period, either resazurin or CellTiter-Glo solution was added to the cells following the manufacturer’s protocol and measured on a TECAN Spark instrument using the Spark control Magellan 3.1 software for measurement. Viability of cells was calculated as a percentage of untreated control. EC_50_ was calculated with the GraphPad Prism program using the log­(inhibitor) vs normalized response fit.

### 2.12 Isolation of Peripheral Blood Mononuclear Cells (PBMCs) and Human Immune Cells

Buffy coats were obtained from the Military University Hospital (ÚVN) Prague. Informed written consent was obtained from each individual enrolled. The PBMCs were isolated from the buffy coats of healthy individuals with SepMate-50 using the Ficoll density gradient centrifugation following the manufacturer’s protocol. The purity of isolated PBMCs was assessed by Panel 1 (Figure S2) staining using flow cytometry. Subsequently, RosetteSep cocktails were used to isolate primary human immune cells of interest from the same buffy coats. These included RosetteSep Human Monocyte Enrichment cocktail, RosetteSep Human T Cell Enrichment cocktail, RosetteSep Human B Cell Enrichment cocktail, and EasySep Direct Human Neutrophil isolation kit. The purity of isolated cells was assessed using flow cytometry by antibodies listed in either Panel 1 (CD3 BUV395, CD56 PE, CD19 APC, CD14 BV421, CD16 BV650, and viability dye) or Panel 2 (CD3 BUV395, CD56 BUV395, CD19 BUV395, CD14 BV421, CD16 BV650, HLA-DR PE-CF594, CD64 AF647 and viability dye).

### 2.13 Preparation of Polarized MDMs

Primary human monocytes were seeded at 1 × 10^6^ cells/well in 1 mL RPMI 1640 complete medium (RPMI medium supplemented with 10% FBS and Glutamax) supplemented with 50 ng/mL M-CSF in a 12-well plate and incubated for 8 days at 37 °C in an incubator with 5% CO_2_ to differentiate them into mature nonpolarized M0-like MDMs. Afterward, the MDMs were polarized into either M1-like MDMs by incubation with 50 ng/mL IFN-γ and 100 ng/mL LPS or M2-like MDMs by incubation with 20 ng/mL IL-4 for 48 h. The purity and phenotype of polarized MDMs were assessed using flow cytometry by antibodies in Panel 3 (CD206 BUV395, CD80 BV650, CD163 BV510, CD14 BV421, HLA-DR PE-CF594, CD68 PE, CD64 AF647, and viability dye).

### 2.14 Treatment of MDMs and Immune Cells with CD64-TPDCs

Polarized MDMs or isolated T cells, B cells, and neutrophils were incubated with the desired concentration of CD64-TPDCs for up to 72 h at 37 °C with 5% CO_2_. Consequently, cells were harvested into microtubes using acutase prior to FACS buffer wash (PBS supplemented with 0.5% bovine serum albumin). The cells were then used for caspase determination, confocal microscopy, and flow cytometry analysis.

### 2.15 Determination of Regulated Cell Death

Harvested cells were diluted in PBS and incubated with the selected Caspase-Glo assay mixture (Caspase-Glo 3/7 Assay or Caspase-Glo 8 Assay) following the manufacturer’s protocol. The assays were measured on a TECAN Spark instrument using the Spark control Magellan 3.1 software for data acquisition and analysis.

### 2.16 Flow Cytometry Analysis

Harvested cells were initially stained with Zombie NIR viability dyes for 30 min at RT in the dark following washing with PBS with 0.5% bovine serum albumin (BSA). Subsequently, cells were stained with either selected antibodies or fluorescent CD64-TPDCs at the desired concentration for 30 min under the same conditions. Finally, cells were washed with FACS buffer, and analysis was carried out using a BD LSRFortessa flow cytometer. Data were acquired using FACS Diva software and analyzed with FlowJo 10.1 software, with specific gating strategies described in Figure S2. For some experiments, cells were treated with Human TruStain FcX blocking solution following the manufacturer’s protocol. Control samples included unstained cells, a positive control for dead cells (cells in PBS with 0.5% BSA incubated for 10 min at 65 °C), FMO, and isotype controls.

### 2.17 Time-Lapse Microscopy and Cytotoxicity Detection

M2-like polarized MDMs were prepared as described above. Cell media were replaced with phenol red-free RPMI1640 medium supplemented with 10% FCS, the tested TPDCs (1 nmol/L), propidium iodide (5 ng/L), and Apotracker Green (400 nmol/L). Images were acquired every 6 h using BioTek Cytation 5 Multi-Mode Reader and Imager (Agilent Technologies) with the 4× objective. The number of apoptotic (Apotracker-positive) and dead (propidium iodide-positive) cells was determined using the Gen5 software (Agilent Technologies) and is expressed as a percentage of the total cell count at the beginning of the experiment.

### 2.18 Confocal Microscopy

Approximately 2 × 10^4^ polarized MDMs were seeded in 200 μL of RPMI complete medium in 96-well glass-bottom plates. The CellLight Late Endosomes-RFP reagent was added to the wells following the manufacturer’s protocol. After overnight incubation at 37 °C in 5% CO_2_, MDMs were stained either with **fC2** or anti-CD64 10.1 AlexaFluor647 antibody, and LysoTracker Green DND-26 dye for the desired period following the manufacturer’s protocol. Twenty minutes before the end of the incubation, 20 μL of 10 μg/mL Hoechst 34,580 in PBS was added to the wells. Samples were washed with 150 μL PBS. Images were captured using Zeiss LSM 980 confocal microscope with Airyscan2 using a 63× immersion oil objective (NA = 1.20) and 1 Airy unit set as a pinhole for each channel. ZEN 3.5 software (Carl Zeiss Microscopy) was used for image processing and analysis. The Pearson’s overlap coefficient for colocalization of CD64-TPDCs in either lysosomes or endosomes was calculated from the entire image.

### 2.19 qPCR Analysis

qPCR analysis and primer preparation are based on procedures described by Pires et al.^47^ and Taylor et al.^48^ Briefly, approximately 2 × 10^6^ MDMs/sample were used for RNA isolation and purification with the Macherey-Nagel NucleoSpin RNA kit following the manufacturer’s protocol. Of the total RNA, 400 ng was used for cDNA synthesis (LunaScript RT SuperMix) following the manufacturer’s protocol. qPCR was performed using Luna Universal qPCR Master Mix and various sets of primers (Table 1) at a final concentration of 0.5 μM. The PCR reaction proceeded as follows using a Light Cycler 480 II qPCR instrument (Roche): 1 cycle of 95 °C for 1 minute; 45 cycles of 95 °C for 15 seconds and 60 °C for 30 seconds; and 1 cycle of 40 °C for 30 seconds. The qPCR data were obtained using the second derivative maxima method in the Light Cycler 480 II Software. The mRNA expression profiles were normalized to GAPDH (glyceraldehyde 3-phosphate dehydrogenase) levels. Additionally, the mRNA expression profiles of MDMs were normalized to that of M1-like MDMs. The relative gene quantities from technical triplicates for one gene/condition were calculated from the ΔCq (i.e., 2^ΔCq^), where it is assumed that reaction efficiency is 100%, and hence a base of two is applied. For each sample combination, a normalization factor is determined from the geometric mean of the associated reference gene relative quantities. The relative normalized expression for each target gene was then calculated per sample by dividing the relative quantity by the normalization factor, followed by log transformation. The average relative normalized and log-transformed expression for each biological group was then calculated using the geometric mean. The geometric means of five independent biological replicates were used to show cathepsin expression in Figure 5A–E.

**Table 1:** List of qPCR Primers

cathepsin B	forward	CCAGGGAGCAAGACAGAGAC
	reverse	GAGACTGGCGTTCTCCAAAG
cathepsin D	forward	GACACAGGCACTTCCCTCAT
	reverse	CTCTGGGGACAGCTTGTAGC
cathepsin K	forward	TTCTGCTGCTACCTGTGGTG
	reverse	GCCTCAAGGTTATGGATGGA
cathepsin L	forward	AGGAGAGCAGTGTGGGAGAA
	reverse	ATCTGGGGGCCTCATAAAAC
cathepsin S	forward	TCTCTCAGTGCCCAGAACCT
	reverse	GCCACAGCTTCTTTCAGGAC
GAPDH	forward	AAGGTGAAGGTCGGAGTCAA
	reverse	AATGAAGGGGTCATTGATGG

### 2.20 Cathepsin K Quantification by Immunohistochemistry

Approximately 1.2 × 10^7^ polarized MDMs were harvested using acutase and cell suspension was centrifuged at 2000*g* for 5 min. Supernatant was discarded, and the pellet was frozen for 24 h. After that, protein in thawed cell lysates was quantified using Bradford assay following the manufacturer’s protocol. Next, 100 μg of protein detected in cell lysates were mixed with reducing sample buffer (100 mM Tris–HCl, pH 6.8; 10% SDS; 20% glycerol; 180 μM bromophenol blue; and 500 mM β-mercaptoethanol) at a 1:5 ratio. Samples were heated for 5 min at 95 °C, and aliquots were loaded onto precast 12% polyacrylamide gels. Electrophoresis was performed in Tris–glycine–SDS buffer at 170 V for 90 min. After electrophoresis, gels were transferred to nitrocellulose membranes (0.45 μm, Bio-Rad) for antibody detection. Wet electroblotting was performed at 100 V for 1 h in Tris–glycine transfer buffer containing 20% methanol. After blotting, nitrocellulose membranes were blocked with Casein Blocker for 1 h at RT. Subsequently, primary Anti-Tubulin α (1:1,000) and Anticathepsin K (1:1,000) antibodies were added and incubated overnight at 4 °C. The next day, membranes were washed three times for 8 min with TBS containing 0.1% Tween-20. Secondary antibody (1:15,000) was then applied for 1 h at 4 °C. Final washes were performed as described above, followed by a single wash with TBS without Tween-20. Blots were visualized using the LI-COR Odyssey CLx imager.

### 2.21 Linker Cleavage by Cathepsins

To assess cleavage of the linkers attaching toxins to HPMA copolymers and to assess the selectivity of cathepsins B, K, L, and S toward these peptide sequences, UPLC-MS analysis was performed on samples after 24 h incubation with these cathepsins. Initially, the reagent with final concentrations of 20 μM TPDCs, 100 μM linker with cytotoxic moiety, 100 nM cathepsins (B, K, L, or S), and 20 μM inner standard (Z-Phe-OH) were mixed in 100 μL of cathepsin assay buffer (100 mM NaAc, pH 5.5, containing 100 mM NaCl, 0.5 mM Na_2_EDTA, and 5 mM DTT) and incubated for 24 h at 37 °C. After the incubation, the reaction mixtures were filtered through the Amicon tubes (10 kDa cutoff), which had been prewashed with 100 μL Milli-Q water by 15- minutes centrifugation at 14,000*g*. The reaction solutions were transferred to the Amicon tubes and centrifuged for 20 min at 14,000*g*. Then, 100 μL Milli-Q water was added, and the filtration columns were centrifuged again. Filtrates were then pipetted into HPLC tubes with inserts and stored at −20 °C before UPLC-MS analysis on a Waters UPLC H-Class Core System [Waters Acquity UPLC BEH C18 column (1.7 μm, 2.1 × 100 mm), Waters Acquity PDA diode array 190–800 nm detector, Waters SQD mass spectrometer, MassLynx software] with a gradient from 0.1% formic acid in water to 0.1% formic acid in acetonitrile. Analysis of chromatograms and spectra was performed with MestReNova software, integrating the area of peaks of free toxins and Z-Phe-OH in the chromatogram using the automated Detect peaks function. From these values, cleavage was calculated according to eq 1

1 $$cleavage\;\left(\%\right)=\frac{\left(\frac{\frac{A_{toxin,sample}}{A_{Z‐Phe}}}{\frac{A_{toxin,standard}}{A_{Z‐Phe}}}\right)\cdot c_{toxin,standard}}{c_{sample}\cdot N_{toxin}}\times 100$$

where *A*_toxin,sample_ is the area of the peak of cleaved toxin in the reaction with a cathepsin, *A*_toxin,standard_ is the area of the peak of toxin in a sample containing only the toxin moiety standard at the concentration of *c*_toxin,standard_, *A*_Z‑Phe_ is the area of the peak of Z-Phe-OH, *c*_sample_ is the concentration of the substrate (TPDCs or linker with toxin moiety), and *N*_toxin_ is the number of possible cleavage sites where either MMAE or Gly-DM1 toxins can be released per molecule of substrates (11.0 for **C4**, 8.6 for **C5**, 10.3 for **C8**, 9.4 for **C9**, and 1 for linker with toxin moiety only).

### 2.22 Statistical Analysis

The binding potency of CD64-TPDCs, evaluated using SPR and DIANA, was determined by one and by two independent biological replicates, respectively. Data obtained by confocal microscopy were representative of three independent experiments. Determination of cell viability detected by flow cytometry was performed with a minimum of three independent biological replicates. Statistical analysis for cell viability assays (CellTiter-Glo Luminescent Cell Viability Assay) was conducted with a minimum of three independent biological replicates using the Shapiro-Wilk test of normality and one-way ANOVA followed by Šídák’s multiple comparison, with a 95% confident interval. Data showing expression of different markers in MDMs are presented as mean ± SD from at least *n* = 5 independent experiments. Statistical significance was determined using the Shapiro-Wilk test of normality following either Welch’s *t*-test or Mann–Whitney *t*-test, depending on normality, both *t*-tests with a 95% confidence interval. Statistical analysis for the determination of cytotoxic potency against MDMs was performed using the Shapiro–Wilk test of normality and 2-way ANOVA, with a 95% confidence interval. Data are presented as mean ± SEM from at least *n* = 4 MDMs samples. Data showing CD64 expression are presented as mean ± SD from at least *n* = 5 MDMs samples. Statistical significance was determined using the Shapiro-Wilk test of normality following 2-way ANOVA followed by Dunnett’s multiple comparisons test. Data showing determination of caspases are presented as mean ± SD from at least *n* = 4 MDMs samples. Statistical significance was determined using the Shapiro-Wilk test of normality following 2-way ANOVA followed by Fisher’s LSD test, with 95% confidence interval. Data of Pearson’s coefficient are presented as mean ± SD from at least eight independent experiments, and data showing relative caspase expression are presented as mean ± SD from six experiments. Statistical significance was determined using the Shapiro–Wilk test of normality and *t*-test, with a 95% confidence interval. Data of cathepsin gene expression are depicted as average normalized and log2-transformed gene expression normalized to M1-like MDMs and presented from at least *n* = 4 MDMs samples. Statistical significance was determined using the Shapiro-Wilk test of normality and the unpaired *t*-test, with a 95% confidence interval. Efficiency of linker cleavage is presented as mean ± SD from at least triplicate. Statistical significance was determined using the Shapiro–Wilk test of normality and one-way ANOVA followed by Tukey multiple comparison test, with a 95% confidence interval. Asterisks are displayed for those with P values less than 0.05. Data with CatK inhibitor are presented as mean ± SD from *n* = 5 MDMs samples. Statistical significance was determined using the Shapiro–Wilk test of normality and *t*-test, with a 95% confidence interval. All statistical analyzes were performed using GraphPad Prism 10.3.0 software.

### 2.23 Chemistry Methods

See Supporting Information for complete synthetic methods and characterization.

Chemicals, reagents, and antibodies used in this study are listed in Table S3.

## 3 Results

### 3.1 A HPMA-Based Copolymer Carrier Scaffold Enables the Development of CD64-TPDCs

To determine whether peptide-mediated targeting provides sufficiently specific and high-affinity binding of TPDCs, and to assess how variations in the conjugated cytotoxic cargo and linker architecture modulate payload release profiles and cytotoxic potency, we prepared a set of CD64-TPDCs, including fluorescent and cytotoxic variants (Figure 1).

**Figure 1:**
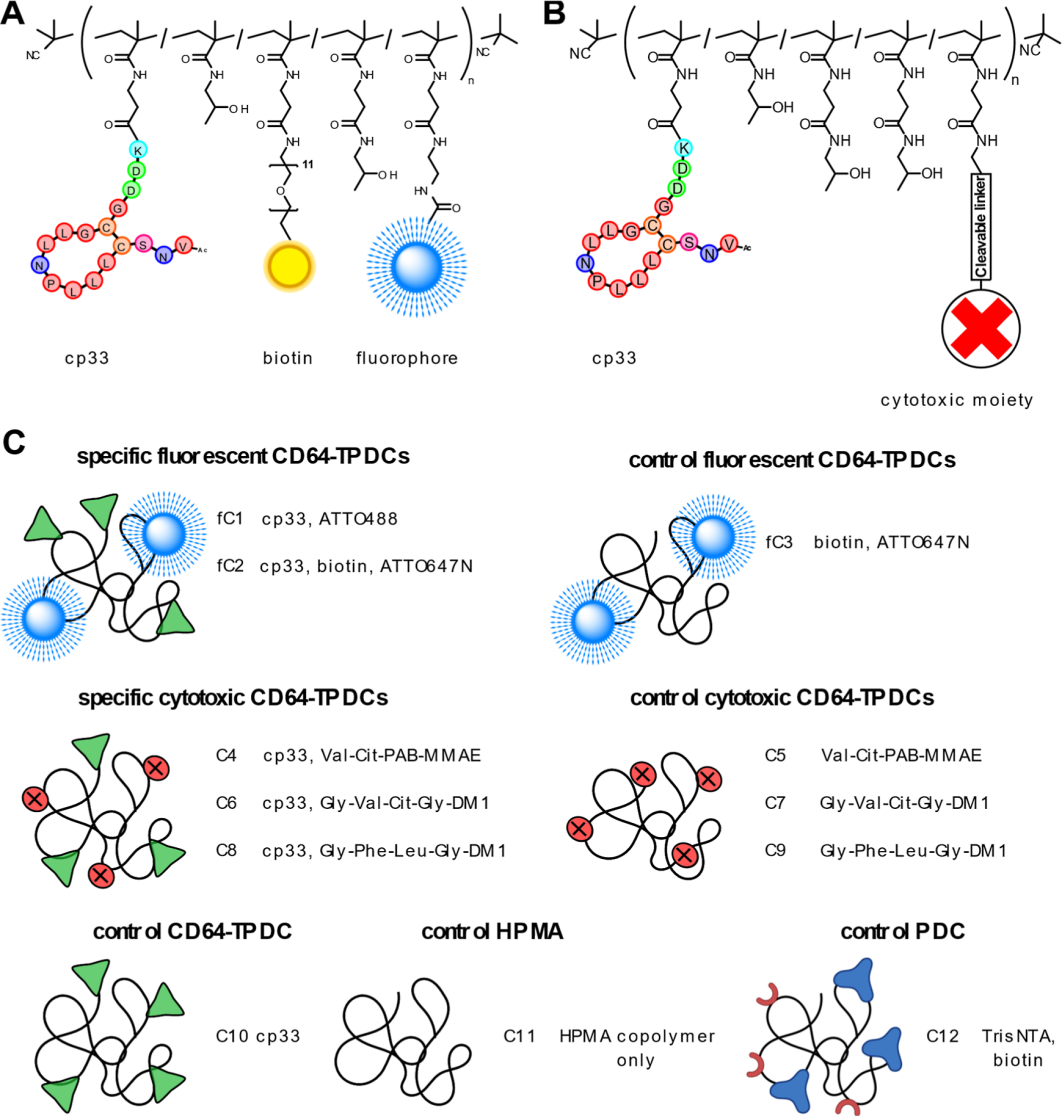
Schematic representation of CD64-specific and control TPDCs. (A) Fluorescent CD64-TPDCs were decorated with biotin and fluorophores (ATTO488 or ATTO647N). (B) Cytotoxic CD64-TPDCs contained a cytotoxic moiety conjugated to the copolymer backbone through a cathepsin-cleavable linker (valine-citrulline-*p*-aminobenzyl alcohol-MMAE, glycine-valine-citrulline-glycine-DM1, or glycine-phenylalanine-leucine-glycine-DM1). (C) List of CD64-TPDCs used in this study, including controls (green mark represents cp33 ligand, red mark represents cytotoxic payload).

Briefly, TPDCs were assembled using aminolytic reactions between the thiazoline-2-thione (TT) groups of copolymer precursors (P1–P4, Table S1) and the amino group of fluorophores, biotins, cytotoxic payloads, and/or cp33 peptide ligands. These aminolytic reactions proceeded nearly quantitatively, as confirmed by compositional analysis (Table S2).

The fluorescent copolymers included **fC1** and **fC2**, functionalized with cp33 and a fluorophore (ATTO488 or ATTO647N), and **fC3**, which lacks the peptide binder and serves as a nontargeted control. For cytotoxicity studies, CD64-targeted TPDCs include **C4**, **C6**, and **C8**, with their corresponding nontargeted controls **C5**, **C7**, and **C9**. **C4** and **C5** contain an MMAE payload linked via Val–Cit-PAB, **C6** and **C7** contain DM1 linked via Gly–Val–Cit-Gly, and **C8** and **C9** contain DM1 linked via Gly–Phe–Leu–Gly. Together, these conjugates were designed to probe how cytotoxic cargo, linker-controlled release, and cp33-mediated targeting contribute to CD64-specific binding and activity, using appropriate nontargeted and noncytotoxic controls. Additional controls included polymeric conjugates with cp33 alone (**C10**) or without any ligands (**C11**). Compound **C12**, containing biotin and TrisNTA was used as a compound linker for binding potency determination. Detailed descriptions of the chemical structures and preparation of CD64-TPDCs are provided in the Supporting Information (Table S1, and chapter Preparation of TPDCs).

### 3.2 Incorporating Multiple CD64-Targeting Ligands into the Single Polymer Chain Significantly Improves the Binding Affinity of the Resulting TPDCs

To assess whether conjugation to the HPMA copolymer enhances cp33 peptide binding to CD64, we established a stable *in vitro* model based on HEK 293 cells with tetracycline-inducible CD64 expression (HEK A2 CD64). Both the small molecule of cp33 ligand conjugated with 6-carboxyfluorescein (**cp33-FAM**) and conjugate **fC1** demonstrated increased binding to HEK A2 CD64 cells, compared to HEK A2 cells (Figure 2A,B). However, **fC1** displayed substantially higher avidity and specificity, with approximately 2 orders of magnitude improvement in concentration–response relationship compared to cp33-FAM (Figure 2B). These data indicated that multivalent presentation of cp33 on the HPMA copolymer greatly enhanced the effective binding of the peptide ligand and supported specific recognition of CD64-expressing cells.

**Figure 2:**
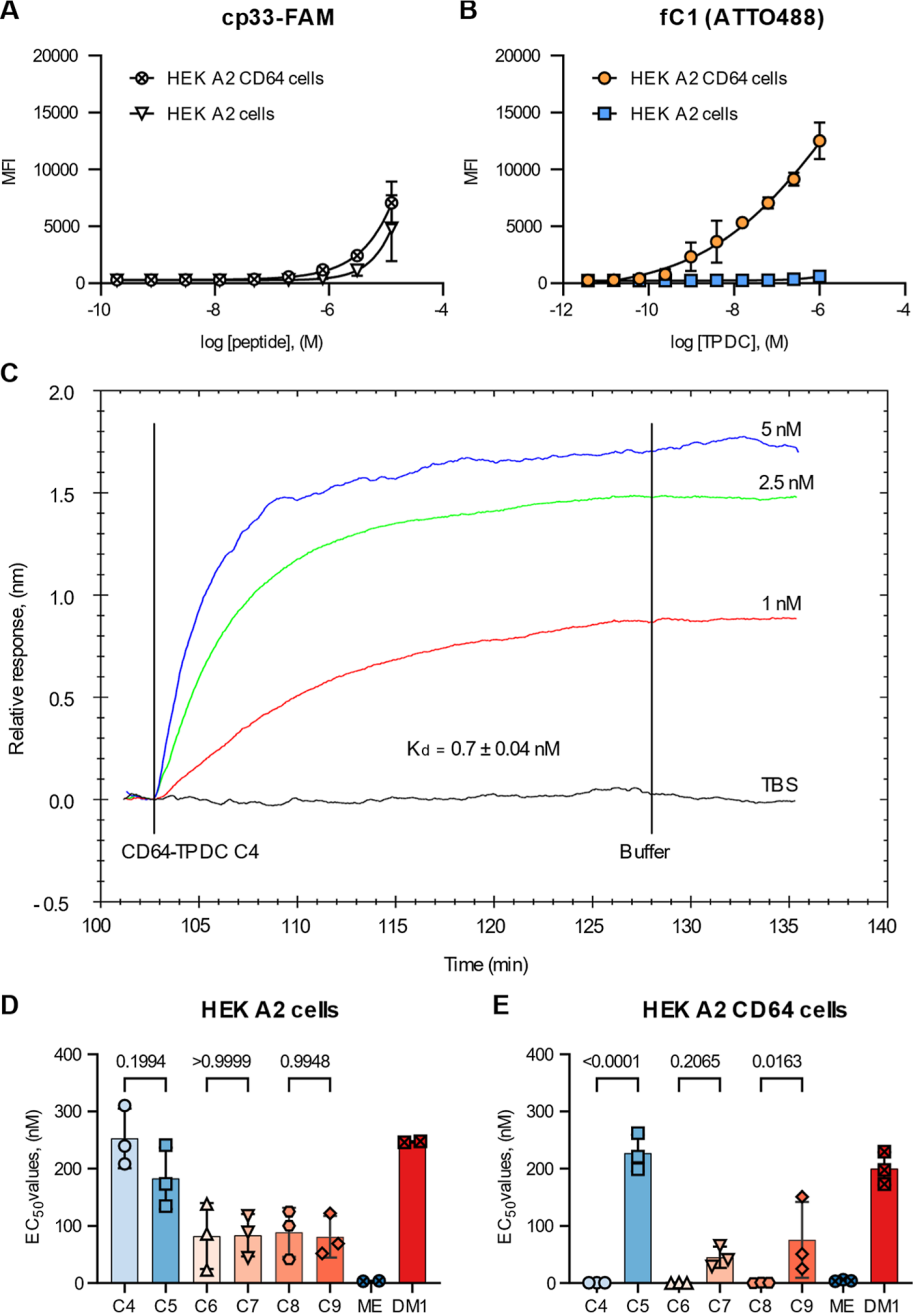
Binding efficacy and cytotoxic potency of CD64-TPDCs. (A) Flow cytometry staining of HEK A2 CD64 and HEK A2 cell lines treated with a dilution series of the cp33-FAM peptide or (B) conjugate **fC1**. Data are displayed as the median fluorescence intensity (MFI) ± SD of at least *n* = 3 independent experiments. The associated gating strategy is provided in Figure S2. (C) Representative example of SPR analysis of **C4** (cp33, Val-Cit-PAB-MMAE) binding to hCD64 protein at three different concentrations. The TBS buffer was used as a negative control. The relative response is proportional to the amount of bound analyte. (D) HEK A2 and (E) HEK A2 CD64 cells were treated with a dilution series of TPDCs **C4** (cp33, Val-Cit-PAB-MMAE), **C5** (Val-Cit-PAB-MMAE), **C6** (cp33, Gly-Val-Cit-Gly-DM1), **C7** (Gly-Val-Cit-Gly-DM1), **C8** (cp33, Gly-Phe-Leu-Gly-DM1), **C9** (Gly-Phe-Leu-Gly-DM1), and the cytotoxic moieties MMAE (ME) and DM1 for 5 days, using the CellTiter-Glo viability assay. Results are presented as mean ± SD of at least *n* = 3 independent experiments. The EC_50_ values were determined with GraphPad Prism using the log­(inhibitor) vs normalized response fit. Statistical analyzes for cell viability assays were conducted using the Shapiro–Wilk test of normality and one-way ANOVA followed by Šídák’s multiple comparison, with a 95% confidence interval.

We further observed that conjugate **fC2** (cp33, biotin, ATTO647N) bound to and was internalized by HEK A2 CD64 cells to a similar extent as a fluorescently labeled anti-CD64 monoclonal antibody (Figure S3). In contrast, we detected no binding of these CD64-TPDCs to CD64-negative cells under these conditions. Control **fC3**, lacking the cp33 targeting ligand, did not bind to CD64-expressing cells, confirming the requirement for the cp33-CD64 interaction (Figure S3).

### 3.3 CD64-TPDCs Recognize the Human CD64 Protein

To further characterize the interaction of CD64-TPDCs with human CD64 protein (hCD64), we used surface plasmon resonance spectroscopy (SPR) and the DNA-linked inhibitory antibody assay (DIANA^46^). Both methods were used to precisely determine the apparent dissociation constant (*K*_d_) and to provide evidence of cp33-containing TPDCs to recombinant hCD64 binding via the peptide ligand.

Both assays consistently demonstrated high-affinity binding of CD64-specific TPDCs to hCD64, with apparent *K*_d_ values in the subnanomolar range (Table 2), whereas the untargeted copolymers lacking cp33 showed only weak or undetectable binding (apparent *K*_d_ > 1 μM or not determined). For SPR, precise kinetic *K*_d_ determination was challenging for some TPDCs due to very slow dissociation rates, resulting in incomplete return to baseline within the measurement window. Therefore, we present copolymer **C4** (cp33, Val-Cit-PAB-MMAE) as a representative example illustrating specific, concentration-dependent binding to an immobilized hCD64 by SPR (Figure 2C). Together with the DIANA data, these results confirm that multivalent cp33-functionalized HPMA copolymers engage hCD64 with high apparent affinity and that this binding is strictly dependent on the presence of the cp33 ligand.

**Table 2:** Overview of Kinetic Parameters of all TPDCs Used in This Study^a^

TPDCs	ligands	*K*_d_ [pM]—SPR	*K*_d_ [pM]—DIANA
**fC1**	cp33, ATTO488	300 ± 260	280 ± 100
**fC2**	cp33, biotin, ATTO647N	1800 ± 180	220 ± 120
**fC3**	biotin, ATTO647N	n.d.	>10^6^
**C4**	cp33, Val-Cit-PAB-MMAE	700 ± 40	60 ± 30
**C5**	Val-Cit-PAB-MMAE	n.d.	>10^6^
**C6**	cp33, Gly-Val-Cit-Gly-DM1	50 ± 50	10 ± 10
**C7**	Gly-Val-Cit-Gly-DM1	n.d.	>10^6^
**C8**	cp33, Gly-Phe-Leu-Gly-DM1	20 ± 160	20 ± 10
**C9**	Gly-Phe-Leu-Gly-DM1	n.d.	>10^6^
**C10**	cp33	290 ± 80	520 ± 350
**C11**	HPMA copolymer only	n.d.	>10^6^
**C12**	TrisNTA, biotin	n.d.	n.d.

a The kinetic parameters of TPDCs (**fC1**–**C12**) were examined using SPR and DIANA. The column “ligands” shows the targeted and functional moieties conjugated to TPDCs **fC1**–**C12**. The approximate *K*_d_ values for SPR and DIANA represent the binding association constant value in pM, as determined by SPR or DIANA. n.d. stands for not determined; “>10^6^’’ indicates that the *K*_d_ is greater than 1 μM or not detectable.

### 3.4 Cytotoxic CD64-TPDCs Eliminate CD64-Expressing Cells *In Vitro*

To evaluate the cytotoxic activity and specificity of cytotoxic CD64-TPDCs, we compared several copolymer designs that differed in cytotoxic payloads and cathepsin-cleavable linkers. TPDCs carried MMAE or DM1 attached to the HPMA backbone via Val-Cit-PAB, Gly-Val-Cit-Gly, or Gly-Phe-Leu-Gly linkers.

All cytotoxic CD64-specific TPDCs (**C4**, **C6**, and **C8**) exerted cytotoxic effects on HEK A2 CD64 cells *in vitro*, while having minimal effect on parental HEK A2 cells lacking CD64 expression (Figure 2D, E). In contrast, control conjugates **C5**, **C7**, and **C9** did not affect the viability of CD64-expressing cells as EC_50_ values detected as subnanomolar (Figure 2E). Using the CellTiter-Glo viability assay, we determined subnanomolar EC_50_ values for TPDCs **C4**, **C6**, and **C8**, representing approximately 2 orders of magnitude higher potency than the corresponding nontargeted controls (Figure 2D, E). These data confirm that CD64-directed copolymers function as potent, target-dependent cytotoxic PDCs.

Conjugation of MMAE and DM1 to the HPMA copolymer backbone led to enhanced toxicity and selectivity of the free toxins, particularly for DM1 (Figure 2E). Thus, both the choice of cytotoxic payload and the design of the cathepsin-cleavable linker are critical parameters in optimizing CD64-TPDCs. Based on its favorable binding potency and cytotoxicity profile, we selected compounds **C8** and its corresponding control **C9** for subsequent mechanistic studies.

### 3.5 CD64-TPDCs do Not Have Cytotoxic Effects on Immune Cells Lacking CD64-Expression

To assess the potential selectivity of cytotoxic CD64-TPDCs toward other immune cell populations, we exposed primary T cells, B cells, and neutrophils to 1 nM CD64-TPDCs for 24, 48, and 72 h (Figure S4A–C). Under these conditions, treatment with copolymers, including **C4** and **C8**, did not affect the viability of CD64-negative cells. Increasing the TPDCs’ concentration to 1 μM likewise did not induce cytotoxicity in these cell types (Figure S4D,E). These data provide evidence that CD64-TPDCs do not bind to and induce apoptosis in immune cells lacking CD64 expression.

### 3.6 Cytotoxic CD64-TPDCs Selectively Trigger Apoptosis in M2-like MDMs

To evaluate the activity of CD64-targeted cytotoxic conjugates in primary myeloid cells, we generated M1-like and M2-like MDMs *in vitro* from the blood of healthy human donors.

Effective polarization was confirmed by flow cytometry-based immunophenotyping of M1-like and M2-like MDMs. M1-like cells expressed higher levels of CD64, HLA-DR, and CD80, whereas M2-like MDMs exhibited upregulation of CD163 and CD206 (Figure S5A). Bright-field microscopy showed distinct morphological differences. M1-like MDMs displayed a round and flattened appearance, while M2-like MDMs exhibited an elongated, “spindle-shaped” appearance (Figure S5B, C), consistent with their respective polarization states.^49^

Following phenotypic validation, M1-like and M2-like MDMs were treated with 1 nM CD64-TPDCs in a time-dependent manner. These cytotoxic conjugates bearing DM1 (**C8**) selectively eliminated M2-like MDMs, a subpopulation phenotypically resembling TAMs (Figure 3A), whereas M1-like MDMs were largely unaffected under the same conditions (Figure 3B). The cytotoxic potency of **C8** was about three times higher on M2-like MDMs compared to M1-like counterparts (Figure 3A, 3B). Control conjugates (**C9**, **C10**, and **C11**) had no detectable impact on MDMs viability, confirming the requirement for CD64-mediated targeting. In contrast to **C8**, the Val-Cit-PAB-MMAE conjugate **C4** did not elicit cytotoxicity in either M1-like or M2-like MDMs (Figure S6), indicating that both the nature of the cytotoxic payload and the linker design influence TPDCs efficacy and that MDM subsets differ in their sensitivity to specific toxins. To exclude differences in CD64 expression or CD64-TPDCs binding as the basis for M2-selective cytotoxicity, we compared the binding of the CD64-targeted fluorescent copolymer **fC2** with that of a commercial anti-CD64 antibody across both MDM subsets (Figure S7). We confirmed that M1-like cells express higher levels of CD64 than M2-like MDMs (Figure 3C), in agreement with published data. Despite that, **fC2** is bound specifically to both subsets, yet with lower signal intensity on M2-like cells. No significant binding was observed with the control copolymer **fC3**, which lacks cp33, confirming ligand-specific interaction (Figure 3C).

**Figure 3:**
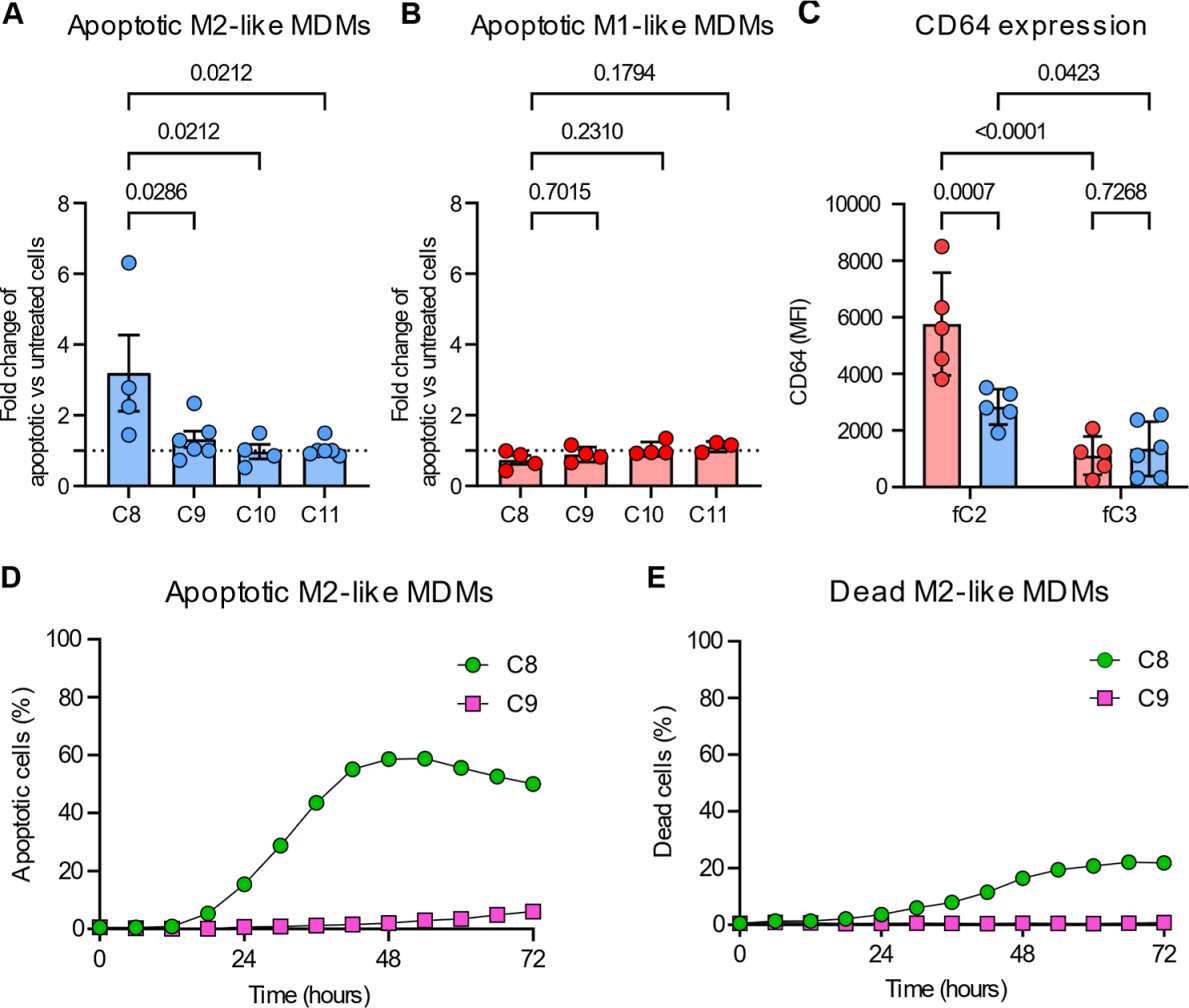
Cytotoxic CD64-TPDCs eliminate MDMs. (A) M2-like and (B) M1-like MDMs after 24 h incubation with 1 nM CD64-targeted cytotoxic conjugate **C8** (cp33, Gly-Phe-Leu-Gly-DM1) or controls **C9** (Gly-Phe-Leu-Gly-DM1), **C10** (cp33), and **C11** (HPMA copolymer). The plotted values represent the relative fold change in apoptotic macrophages in CD64-TPDCs-treated samples normalized to the corresponding untreated control within the same macrophage condition, indicated by the dotted line. Data are presented as mean ± SEM from at least *n* = 4 MDMs samples. Statistical significance was determined using the Shapiro–Wilk test of normality and 2-way ANOVA, with a 95% confidence interval. (C) Binding of TPDCs **fC2** (cp33) and **fC3** to M1-like and M2-like MDMs. Data are presented as mean ± SD at least *n* = 5 MDMs samples. Statistical significance was determined using the Shapiro–Wilk test of normality and 2-way ANOVA, with a 95% confidence interval. The gating strategy is provided in Figure S2. (D) Quantification of apoptotic, and (E) dead M2-like MDMs treated with **C8** (cp33, Gly-Phe-Leu-Gly-DM1; 1 nM) and the control **C9** (Gly-Phe-Leu-Gly-DM1; 1 nM) from Figure S9.

To define the death pathway engaged by **C8**, we assessed caspase-3/7 and caspase-8 activation in MDMs, as DM1 is classically associated with mitotic arrest and apoptosis. Caspase activities were measured after 8, 12, and 24 h of exposure to **C8**. Across all time points, caspase-3/7 and caspase-8 activities remained low and did not differ significantly from untreated or PDCs-treated controls, although a modest upward trend in caspase-3/7 activity was observed at 24 h (Figure S8). These results suggest that, under these conditions, biochemical caspase assays do not capture a robust apoptotic signal.

We therefore performed time-lapse microscopy of apoptosis and cell death in M2-like MDMs over 72 h using Apotracker and propidium iodide (Figures 3D,E; S9). Treatment with the control conjugate **C9** did not induce relevant apoptotic response. In contrast, **C8** caused a clear, time-dependent increase in apoptotic and dead cells, detectable as early as 6 h after treatment and progressively increasing thereafter (Figure 3D,E), consistent with apoptosis as the predominant mode of **C8**-induced cell death in M2-like MDMs.

Collectively, these data demonstrate that CD64-TPDCs bearing DM1 selectively eliminate M2-like MDMs while largely sparing M1-like counterparts. This M2-restricted activity supports the concept that CD64-targeted, enzyme-responsive PDCs can be engineered for preferential depletion of immunosuppressive macrophages in the tumor microenvironment.

### 3.7 CD64-TPDCs Undergo Rapid and Efficient Lysosomal Accumulation in M2-Like MDMs, but Not M1-Like Cells

To investigate why the cytotoxic CD64-TPDC **C8** selectively elicits cytotoxicity in M2-like MDMs while sparing their M1-like counterparts despite their higher CD64 expression, we examined TPDCs underlying mechanism of action, focusing on differences in internalization and intracellular trafficking distribution. We used the fluorescently labeled conjugate **fC2**, which carries cp33 and a fluorophore but no cytotoxic payload, as a tracer of CD64-TPDCs trafficking. M1-like and M2-like MDMs were treated with **fC2** or anti-CD64 antibody and, at defined time points, stained for lysosomes, endosomes, and nuclei to assess colocalization of the **fC2** with these compartments (Figures 4; S10–S12).

**Figure 4:**
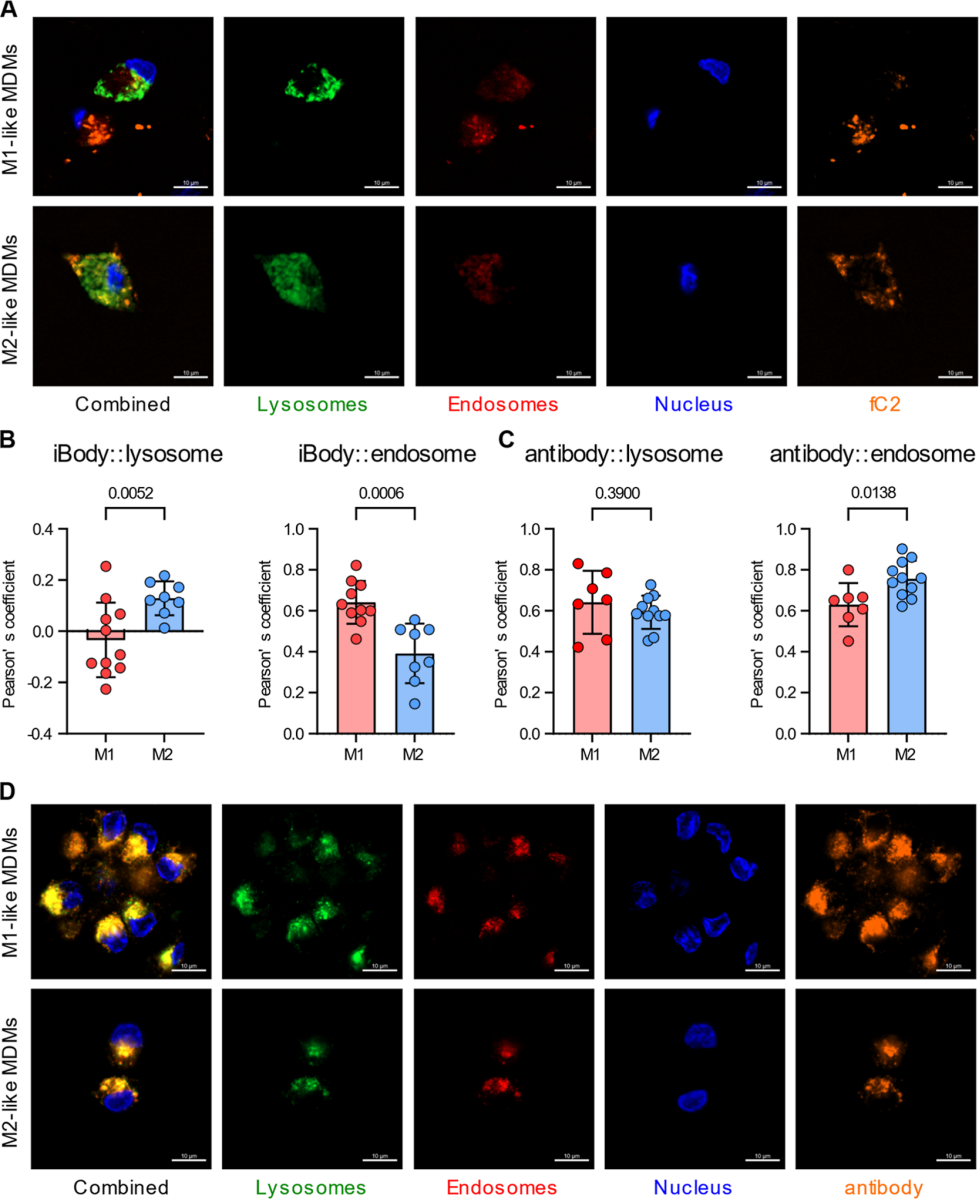
Internalization and localization of fluorescent CD64-TPDCs compared to anti-CD64 antibody within MDMs. (A) M1-like and M2-like MDMs were stained either with 200 nM conjugate **fC2** or, (D) anti-CD64 antibody conjugated with Alexa Fluor 647 (oranget) for 24 h with subsequent staining of lysosomes (green), endosomes (red), and nuclei (blue). Scale bars correspond to 10 μm. Data are representative of three independent experiments from *n* = 3 MDMs samples. (B) Comparison of Pearson’s coefficient for both M1-like and M2-like MDMs for colocalization of TPDCs, or (C) anti-CD64 antibody conjugated with Alexa Fluor 647 in lysosomes and endosomes. Statistical significance was determined using the Shapiro–Wilk test of normality and the *t*-test, with a 95% confidence interval.

Conjugate **fC2** underwent efficient lysosomal internalization in M2-like MDMs, with prominent lysosomal localization detectable within the first 60 min. In contrast, in M1-like MDMs, **fC2** remained largely at the cell surface or within endosomal compartments during this period. After 24 h, M2-like MDMs displayed a markedly higher degree of lysosomal localization of **fC2**, whereas M1-like MDMs retained the **fC2** copolymer predominantly within endosomal structures (Figure 4A). Quantitative colocalization analysis using Pearson’s correlation coefficients confirmed significantly greater colocalization of **fC2** with lysosomal markers in M2-like cells (*r* ∼ 0.3 vs ∼0 in M1-like MDMs), whereas M1-like MDMs displayed higher colocalization with endosomal markers (*r* ∼ 0.7 vs ∼0.4 in M2-like cells; Figure 4B), consistent with preferential lysosomal routing of **fC2** in M2-like macrophages and retention in endosomal compartments in M1-like macrophages.

We next compared the trafficking of **fC2** with a fluorescently labeled anti-CD64 monoclonal antibody. The antibody bound and internalized efficiently into both MDM subsets within 60 min. After 24 h, it showed robust colocalization with both early/late endosomal and lysosomal markers in M1-like and M2-like MDMs (Figures 4C,D; S12). Pearson’s colocalization analysis revealed similar lysosomal association of the antibody in M1-like and M2-like cells (*r* ∼ 0.6 in both subsets), whereas endosomal colocalization was modestly higher in M2-like MDMs (*r* ∼ 0.8 vs 0.6 in M1-like cells).

In contrast, **fC2** displayed virtually no lysosomal colocalization in M1-like MDMs (*r* ∼ 0), but substantial lysosomal association in M2-like MDMs (*r* ∼ 0.3), together with an increase in endosomal colocalization (*r* ∼ 0.7 vs 0.4) preferentially in M1-like cells (Figure 4).

Thus, although both CD64-TPDCs and anti-CD64 internalize more efficiently into M2-like macrophages, only the multivalent polymer conjugate is rerouted to lysosomes in an M2-selective manner, whereas the antibody traffics to lysosomes in both subsets without detectable polarization-dependent bias.

These findings reveal distinct internalization and trafficking pathways for CD64-TPDCs and anti-CD64 antibody in macrophage subsets. Preferential lysosomal delivery of **fC2** in M2-like MDMs, together with limited lysosomal routing in M1-like cells, provides a plausible mechanistic basis for the selective cytotoxicity of DM1-bearing TPDCs toward M2-like macrophages and may help rationalize the variable activities reported for CD64-directed ADCs.

### 3.8 Design of Cathepsin-Cleavable Linkers Determines Their Intracellular Cleavage Efficiency

We then hypothesized that the differential lysosomal accumulation of CD64-TPDCs in M1-like and M2-like MDMs could be affected by subset-specific expression of lysosomal cathepsins and, therefore, by differences in linker processing and payload release. Because both MMAE- and DM1-based TPDCs require proteolytic cleavage of their peptide linkers to release the cytotoxic payload, we focused on cathepsins reported to cleave the Val-Cit-PAB (used in **C4** and **C5**), and Gly-Phe-Leu-Gly (used in **C8** and **C9**) linkers. We first quantified mRNA expression of cathepsins B (CatB), K (CatK), L (CatL), S (CatS), and D (CatD) in M1-like and M2-like MDMs by qPCR (Figure 5A–E). Most cathepsins were expressed at comparable levels in the two MDM subsets. However, CatK expression was approximately 10-fold higher in M2-like cells than in M1-like MDMs (Figure 5B). To validate these findings at the protein level, we analyzed CatK expression in cell lysates from both MDM subsets by immunoblotting. As shown in Figure 5F, CatK was detected in both M1-like and M2-like MDMs, but its abundance was consistently higher in M2-like than in M1-like cells from the same donor. This difference was observed across all five donor-derived samples and was quantified relative to the loading controls α-tubulin (Figures 5F; S13).

**Figure 5:**
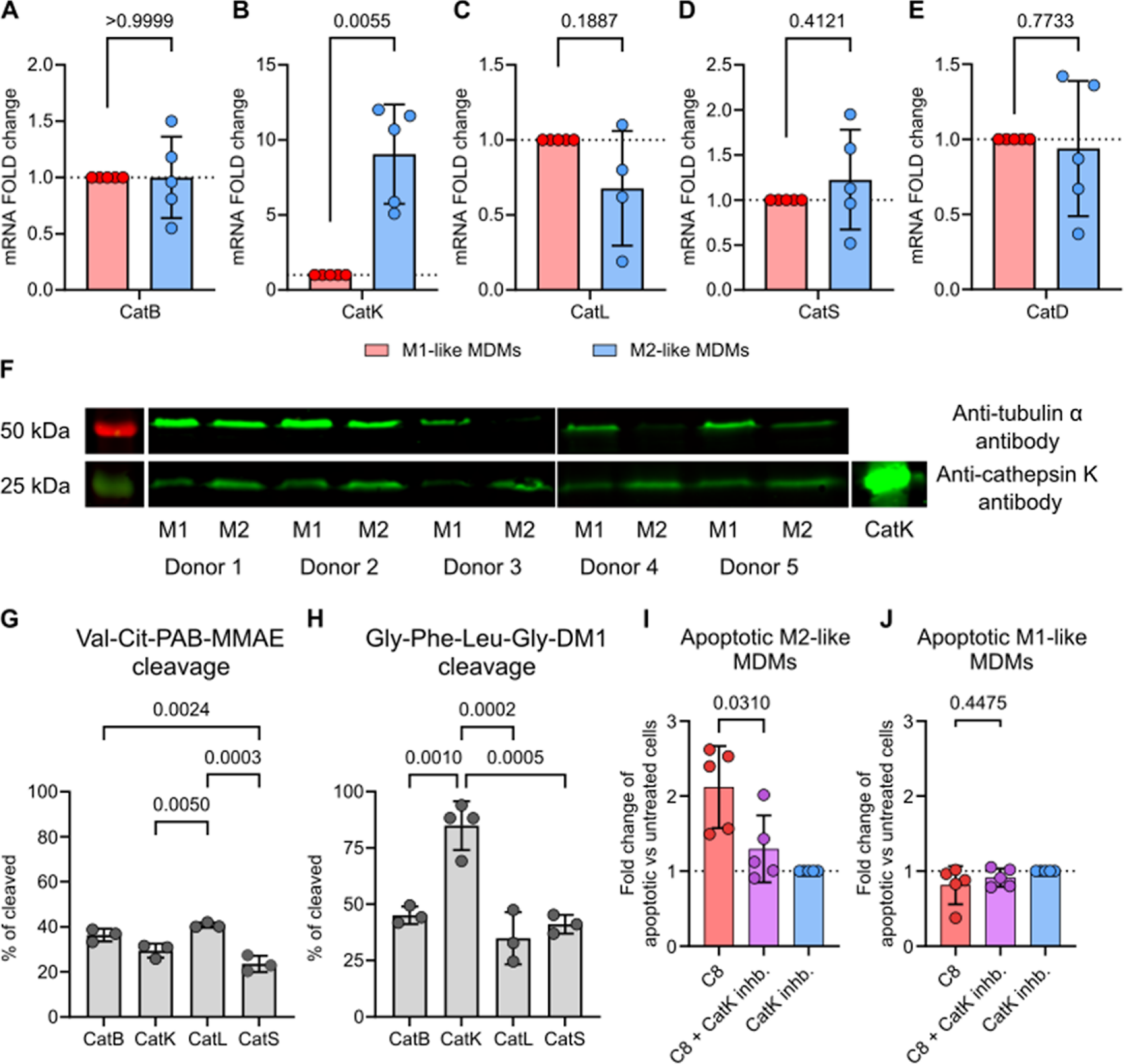
Cathepsin gene expression and efficacy of TPDCs linker cleavages. (A–E) Gene expression of cathepsin B (A), K (B), L (C), S (D), and D (E) in M1-like or M2-like MDMs. Values are depicted as average normalized and log2-transformed gene expression relative to M1-like MDMs. Presented data from at least *n* = 4 MDMs samples. Statistical significance was determined using the Shapiro–Wilk test of normality and the unpaired *t*-test, with a 95% confidence interval. (F) Cathepsin K protein quantification in lysates of MDMs. As a positive control, CatK was used (200 ng). Molecular weights are indicated on the left. 100 ng of lysates were used. Data are presented from *n* = 5 MDMs donor samples. (G) Efficiency of cleavage of Val-Cit-PAB-MMAE and (H) Gly-Phe-Leu-Gly-DM1 linkers conjugated to HPMA-copolymers by individual cathepsins B, K, L, and S. Data are presented as mean ± SD from at least triplicates. Statistical significance was determined using the Shapiro–Wilk test of normality and one-way ANOVA followed by Tukey multiple comparison test, with a 95% confidence interval. Asterisks are displayed for those with *P* values less than 0.05. (I) M2-like MDMs and (J) M1-like MDMs after 24 h incubation with the cytotoxic CD64-TPDCs **C8** (cp33, Gly-Phe-Leu-Gly-DM1; 1 nM), CatK inhibitor odanacatib (100 nM), or both. The number of apoptotic cells induced by **C8** is normalized to cells treated with CatK inhibitor only, indicated by the dotted line. Data are presented as mean ± SD from *n* = 5 MDMs samples. Statistical significance was determined using the Shapiro–Wilk test of normality and *t*-test, with a 95% confidence interval. The associated gating strategy is provided in Figure S2.

Next, to assess the ability of these enzymes to cleave the peptide linkers, we performed end point *in vitro* cleavage assays under conditions mimicking the lysosomal environment and quantified the released payload by UPLC–MS (Figure 5G,H). After 24 h of incubation, used here as the end point of cleavage assays, the Gly-Phe-Leu-Gly linker showed higher overall cleavage efficiency than the Val-Cit-PAB linker when incubated with individual cathepsins. Approximately 40–85% of free DM1 was released from the Gly-Phe-Leu-Gly-DM1 construct, whereas only approximately 40% of MMAE was released from the Val-Cit-PAB-MMAE construct. For the Val-Cit-PAB linker, all tested cathepsins exhibited similar cleavage efficiency (approximately 30–40%; Figure 5G). In contrast, cleavage of the Gly-Phe-Leu-Gly linker was highest with CatK (∼85%), which showed approximately 40% higher cleavage than the other cathepsins (Figure 5H). These data indicate that CatK is a particularly effective protease for processing Gly-Phe-Leu-Gly linkers and to cytotoxic payload release under these assay conditions.

To test the functional relevance of CatK for CD64-TPDC activity in MDMs, we examined the effect of the CatK inhibitor odanacatib on **C8**-induced cytotoxicity in M1-like and M2-like cells. Co-treatment with odanacatib and **C8** significantly reduced the fraction of apoptotic M2-like MDMs compared with **C8** alone, whereas odanacatib by itself had no cytotoxic effect (Figure 5I,J). In contrast, C8, either alone or in combination with odanacatib, did not induce apoptosis in M1-like MDMs, consistent with the lower CatK abundance in these cells relative to their M2-like counterparts (Figure 5J).

Thus, the combination of elevated CatK expression in M2-like MDMs and the higher end point cleavage of the Gly-Phe-Leu-Gly linker observed with CatK supports a model in which CatK contributes to efficient DM1 release and selective cytotoxicity of **C8** in this subset. More broadly, these findings underscore the importance of matching linker chemistry to the protease repertoire of specific target cells when designing next-generation ADCs and TPDCs.

## 4 Discussion

In recent years, TAMs have been increasingly recognized as key regulators of tumor progression and response to therapy, and many myeloid-directed strategies aim to reprogram or deplete these cells in cancer and chronic inflammatory diseases.^3–9^ In many solid tumors, macrophages with an M2-like, immunosuppressive phenotype can comprise a substantial fraction of the tumor mass and are associated with poor clinical outcome.^5,21,50^ Within this context, CD64, a high-affinity FcγRI expressed on myeloid cells and upregulated in several malignancies, has emerged as a potential target for myeloid-directed therapies.

In this study, we exploited CD64 to develop cytotoxic CD64-TPDCs based on biocompatible HPMA copolymers as a fully synthetic, water-soluble alternative to ADCs for myeloid-directed targeting. CD64-TPDCs provide a modular platform for selective targeting and eliminating CD64-expressing myeloid cells, including MDMs. The HPMA scaffold enables simultaneous conjugation of targeted ligands, cytotoxic payloads, and additional functional moieties, thereby allowing preparation of tunable multifunctional constructs. Control over molecular weight, dispersity, and structure may help optimize pharmacokinetic and pharmacodynamic properties to exert sufficient anticancer activity.^38,39^ HPMA copolymers are nonimmunogenic and do not induce proinflammatory cytokine production *in vitro* or *in vivo*,^34,35^ and have been shown to passively accumulate in tumors via the enhanced permeability and retention (EPR) effect in several preclinical models.

On this basis, CD64-targeted, enzyme-responsive HPMA conjugates could access tumor-associated myeloid populations *in vivo* and may provide a route toward selective depletion of immunosuppressive TAMs, a hypothesis that will require direct evaluation in relevant tumor models.

For selective CD64 targeting, we employed previously reported small cyclic peptide cp33, which antagonizes CD64-mediated effector functions and binds to a similar epitope as an anti-CD64 H22 antibody, albeit with lower affinity.^36^ Consistent with our previous work on other receptor ligands,^37,51^ multivalent presentation of cp33 on the HPMA backbone markedly increased its potency and apparent affinity, most likely through avidity effects, and improved solubility due to the hydrophilic nature of the polymer.^39,52,53^ Fluorescent CD64-TPDCs bound and internalized into CD64-expressing cells with similar specificity to a commercial anti-CD64 monoclonal antibody and showed no detectable binding to CD64-negative immune populations, including neutrophils, T cells, and B cells. Control PDCs lacking cp33 did not bind to CD64-expressing cells *in vitro*, as confirmed by biophysical assays (SPR and DIANA) and cell-based analyzes. Furthermore, CD64 is a high-affinity receptor for IgG1, IgG3, and IgG4, with a *K*_d_ on the order of 10^–8^ M. Based on our SPR and DIANA data, CD64-TPDCs engage CD64 with apparent *K*_d_ values in the nanomolar to subnanomolar range, suggesting that they may be capable of competing with endogenous IgG for CD64 binding *in vivo*.^54,55^

In the present study, we synthesized several variants of cytotoxic CD64-TPDCs (**C4**, **C6**, and **C8**) bearing MMAE or DM1 payloads linked via Val-Cit-PAB, Gly-Val-Cit-Gly, or Gly-Phe-Leu-Gly cathepsin-cleavable linkers to assess their ability to eliminate CD64-expressing cells. All CD64-targeted variants efficiently eliminated HEK A2 CD64 cells *in vitro*, with minimal effect on CD64-negative parental HEK A2 cells. Compared with the free toxins, conjugation to the HPMA backbone enhanced the potency and target selectivity, particularly for DM1. As shown in Figure 2D,E, CD64-TPDCs achieved EC_50_ values around 1 nM, whereas HPMA copolymers bearing cytotoxic moieties but lacking any targeting ligands displayed EC_50_ values near 1 μM, consistent with previous reports.^56–58^ This ∼1000-fold increase in potency underscores the impact of incorporating a targeting ligand and supports the utility of HPMA-based PDCs for efficient delivery of highly potent payloads.

Targeting macrophage subpopulations in the TME represents a promising strategy to enhance antitumor immunity. Our findings indicate that the DM1-conjugated CD64-TPDC **C8** selectively eliminates M2-like MDMs while sparing M1-like cells, thereby shifting the balance of the macrophage population toward a more pro-inflammatory phenotype *in vitro*. This M2-selective cytotoxicity likely arises from differential expression of lysosomal proteases, such as cathepsin K, which we and others have shown to be elevated in M2-like macrophages,^59,60^ and from polarization-dependent differences in intracellular trafficking. By preferentially eliminating immunosuppressive M2-like macrophages, **C8** has the potential to reprogram the TME to favor antitumor responses, overcoming a major barrier to effective immunotherapy. Importantly, the inactivity of the control **C9** confirms that this effect is both target- and payload-specific, highlighting the potential of cytotoxic CD64-TPDCs to modulate macrophage subsets with limited off-target effect. At the same time, the clinical use of DM1- or MMAE-based macrophage-targeted agents will require careful safety evaluation, because macrophages are broadly distributed in healthy tissues and contribute to host defense. The approximately 3 orders of magnitude difference in potency between targeted and nontargeted TPDCs in our study suggests that CD64-mediated delivery can substantially improve selectivity *in vivo*.

Although CD64 is not an M2-specific marker, our data show that subset selectivity can emerge from differences in intracellular trafficking and linker processing after receptor engagement. Nevertheless, markers with stronger M2-biased expression, such as CD206 or CD163, represent attractive alternatives for future generations of macrophage-targeted conjugates^61–63^ and warrant direct comparison with CD64-based systems. Conversely, because CD64 is highly expressed on activated inflammatory myeloid cells, the same platform could also be adaptable to settings in which selective targeting of pro-inflammatory macrophages is therapeutically desirable such as autoimmune diseases.

Across donors, analysis of caspase-3/7 and caspase-8 activation at 8, 12, and 24 h after **C8** treatment suggested a trend toward increased caspase activity, although these changes did not reach statistical significance. In contrast, time-lapse imaging with Apotracker and propidium iodide revealed a clear, time-dependent accumulation of apoptotic and dead cells beginning as early as 6 h and progressing over 72 h. Taken together, these findings support apoptosis as a major component of **C8**-induced cell death in CD64-positive macrophages. At the same time, they highlight technical and biological factors, such as donor-to-donor variability, the possibility that caspase activation is transient and may peak before or between the sampled time points, and heterogeneous responses within the macrophage population, that may limit the sensitivity of end point caspase assays in primary MDMs. Nonetheless, the incomplete caspase activation indicates that additional or alternative cell death pathways may also contribute. Future studies using a broader panel of apoptosis and cell death markers will be required to fully delineate the mechanisms underlying **C8**-mediated cytotoxicity.

In addition to apoptotic signaling, our data highlight internalization and intracellular routing of CD64-targeted constructs as one of the critical determinants of macrophage subset selectivity. Notably, previous CD64-directed immunotherapeutics based on antibodies have frequently shown limited efficacy against M2-like macrophages. Several anti-CD64 ADCs derived from the humanized H22 antibody or its single-chain fragment (scFv H22), and conjugated to plant or bacterial toxins such as ricin A, Pseudomonas exotoxin A, granzymes B and M, angiogenin, or tau, predominantly eliminated pro-inflammatory M1 macrophages while sparing M2 macrophages, or exhibited limited cytotoxic effects overall.^26–29,31,32,64–67^ Hristodorov et al. proposed that this M1-restricted activity reflects differential internalization and routing of CD64-ADCs in M1 versus M2 macrophages, with higher intracellular proteolytic activity in M2 cells potentially leading to complete degradation of CD64-ADCs before the payload can exert its effect, whereas partial degradation in M1 macrophages permits cytotoxic activity.^32,68^ This apparent difference from the findings of Hristodorov et al. may reflect differences in construct design rather than a true biological contradiction. Their study used antibody-based CD64 conjugates carrying protein toxins, whereas our system uses a multivalent HPMA scaffold with a small cyclic ligand, a cleavable peptide linker, and a low-molecular-weight tubulin-binding payload. Unlike protein-based toxin constructs, the HPMA backbone is not expected to be readily degraded by intracellular proteases and may therefore remain more stable in the more proteolytically active M2-like macrophages. These differences are likely to affect receptor clustering, endosomal processing, lysosomal delivery, and susceptibility to intracellular degradation.

Consistent with the model proposed by Hristodorov et al.,^32^ our findings show that the intracellular trafficking of CD64-TPDCs differs from that of conventional anti-CD64 antibodies and is strongly polarization-dependent. In M2-like MDMs, CD64-TPDCs are internalized and preferentially routed to lysosomes, where proteases can efficiently cleave the peptide linker and release DM1. In M1-like MDMs, however, CD64-TPDCs showed greater accumulation in endosomal compartments, which likely restricts payload activation and limits cytotoxicity. In contrast, the anti-CD64 antibody displays robust colocalization with both endosomal and lysosomal markers in M1-like and M2-like MDMs, without a detectable polarization-dependent bias in lysosomal targeting. These observations suggest that the multivalent architecture of CD64-TPDCs, through simultaneous engagement of multiple cp33 ligands, may promote immune complex-like clustering of CD64 and favor lysosomal targeting, whereas classical antibody binding favors trafficking through nonselective endosomal pathways.^69^ Such polarization-dependent differences in intracellular fate are biologically plausible, as macrophage polarization influences endocytic activity, vesicle maturation, lysosomal function, and protease composition. As a result, the same receptor-targeted construct may undergo distinct intracellular processing in M1-like and M2-like macrophages after internalization. Together, these data underscore the importance of both the rate and route of internalization as one of critical determinants of payload efficacy in CD64-TPDCs and related polymer-drug conjugates and provide a mechanistic basis for their selective activity toward M2-like macrophages.

To further explain the M2-restricted cytotoxicity of CD64-TPDCs, we also examined to what extent differences in lysosomal protease context influence payload release from the conjugates. We thus investigated the role of proteolysis by profiling cathepsin expression and evaluating linker cleavage by individual cathepsins. qPCR analysis revealed similar expression of cathepsins B, L, S, and D in M1- and M2-like MDMs, whereas CatK was markedly upregulated at the mRNA level in M2-like MDMs. At the protein level, CatK was abundantly detected in M2-like MDMs, compared to M1-like counterparts. *In vitro* cleavage assays showed that the Gly-Phe-Leu-Gly linker was more efficiently processed than the Val-Cit-PAB linker under lysosome-like conditions, with CatK displaying the highest activity toward the Gly-Phe-Leu-Gly linker. These end point *in vitro* cleavage assays were performed at a single enzyme concentration and thus do not provide the full kinetic information between cathepsins. However, approximately 85% of DM1 was released from the Gly-Phe-Leu-Gly-DM1 construct after 24 h, compared with approximately 40% of MMAE from the Val-Cit-PAB-MMAE construct, as CatK showed the highest end point cleavage of the Gly-Phe-Leu-Gly linker among the tested cathepsins. Pharmacological inhibition of CatK with Odanacatib rescued **C8**-induced apoptosis in M2-like MDMs, while odanacatib alone had no cytotoxic effect. Taken together, within the limitations of pharmacological inhibition and *in vitro* cleavage assays, these findings support a model in which CatK is a major contributor to Gly-Phe-Leu-Gly linker processing and DM1 release in M2-like macrophages, with additional cathepsins and elevated lysosomal activity likely providing complementary contributions. Thus, our study highlights the importance of both linker chemistry and payload selection in CD64-targeted therapies. Earlier ADCs bearing bacterial or plant-derived toxins often showed limited or M1-restricted cytotoxicity,^29,31,32,65,66^ whereas a more recent anti-CD64 ADC incorporating a pyrrolobenzodiazepine (dPBD) derivative effectively depleted TAMs *in vivo*,^25^ which is consistent with our observation that CD64-TPDCs can be tuned for M2-like MDMs selectivity by matching linker susceptibility and payload activation to the protease milieu. These comparisons underscore the need for rational design of cytotoxic payloads and enzyme-responsive linkers that are compatible with the trafficking routes and protease repertoire of the intended target cell population and suggest that other toxin classes may offer additional opportunities for macrophage-selective targeting.

While our data support a mechanistic model for M2-selective elimination by CD64-TPDCs and suggest design principles for CD64-targeted payloads, the present study has several limitations. All mechanistic studies were performed in MDMs generated from healthy donor blood *ex vivo*, which are subject to interdonor variability and may not fully recapitulate the phenotype of tissue-resident TAMs in different tumor types.^70^ *In vivo* macrophages, particularly TAMs, may differ not only in CD64 expression but also in cathepsin expression and activity, and in endocytic trafficking pathways, which could influence therapeutic efficacy. Moreover, we did not directly assess *in vivo* biodistribution, tumor accumulation, or TAMs depletion by CD64-TPDCs. In addition, although our confocal imaging supports polarization-dependent differences in intracellular routing, higher-resolution imaging and further quantitative colocalization analyzes will be needed to define these trafficking pathways more precisely. Finally, our analysis of cell death pathways relied on a limited set of apoptosis markers and did not systematically address caspase-independent mechanisms or longer-term consequences for macrophage function. Addressing these points in relevant *in vivo* tumor models and with more comprehensive cell death and safety profiling will be essential to translate CD64-TPDCs toward clinical application.

## 5 Conclusion

Our data show that cytotoxic CD64-TPDCs provide a modular, fully synthetic platform for the preferential elimination of CD64-expressing M2-like MDMs, while sparing pro-inflammatory M1-like MDMs and CD64-negative immune cells. HPMA-based carriers offer favorable physicochemical and immunological properties, and their design integrates CD64 targeting, subset-specific intracellular trafficking, and CatK–associated linker processing to achieve preferential killing of M2-like macrophages. These properties position CD64-TPDCs as promising candidates for the development of myeloid-targeted, enzyme-responsive PDCs aimed at preferentially depleting immunosuppressive macrophages in cancer.

## Supplementary Material







## Data Availability

The data sets used and analyzed during the current study are available from the corresponding author upon reasonable request.

## Notes

The Supporting Information, provided as a separate file, includes additional figures, tables, and experimental procedures related to the chemical synthesis and characterization of CD64-TPDCs and their payloads. Specifically, it contains the chemical structures of the targeted and functional moieties conjugated to CD64-TPDCs (Figure S1); flow cytometry gating strategies (Figure S2); comparison of the binding potency and cellular staining of CD64-TPDCs and anti-CD64 antibodies (Figures S3 and S7); interaction of CD64-TPDCs with immune cells (Figure S4); phenotypic characterization of MDMs (Figure S5); cytotoxic potency of CD64-TPDCs bearing alternative linkers and cytotoxic payloads (Figure S6); detection of caspase activation induced by CD64-TPDCs (Figure S8); time-lapse microscopy of M2-like MDMs treated with CD64-TPDCs (Figure S9); time-dependent internalization and intracellular localization of CD64-TPDCs and anti-CD64 antibody in MDMs (Figures S10–S12); quantification of cathepsin K in MDM lysates (Figure S13); detailed characterization of HPMA copolymer precursors (Table S1) and CD64-TPDCs (Table S2); a list of reagents (Table S3); and detailed synthetic procedures, analytical characterization, and chromatograms of CD64-TPDCs, payloads, and the targeted and functional moieties used in this study (PDF).
CD64-TPDC C8 (MP4)
CD64-TPDC C9 (MP4)
