# Supplementary material for: CD64-Targeted Polymer-Drug Conjugates Exploit Cathepsin K‑Dependent Payload Release for Selective Elimination of Immunosuppressive Macrophages
Source: Mol Pharm. 2026 Apr 21;23(5):3114–30. doi: 10.1021/acs.molpharmaceut.5c01931 (PMC13147327; doi:10.1021/acs.molpharmaceut.5c01931)
Supplement: Supplementary file 1 [file mp5c01931_si_001.pdf]

Supporting Information for:

CD64-targeted polymer-drug conjugates exploit cathepsin K-dependent payload release for selective elimination of immunosuppressive macrophages

Dominik Musil, Markéta Krhutová, Kristýna Blažková, Anežka Kramná, Andrea Brázdová, Barbora Výmolová, Magdalena Houdová Megová, Martin Hadzima, Robin Kryštůfek, Vladimír Šubr, Libor Kostka, Tomáš Etrych, Tereza Ormsby, Pavel Šácha, Jakub Abramson, and Jan Konvalinka.

Supplementary Material

Table of Contents

Supplementary Figure S1. Chemical structures of targeted and functional moieties 3
Supplementary Figure S2. Gating strategy for flow cytometry analysis 4
Supplementary Figure S3. CD64-TPDCs stain cells similarly to anti-CD64 antibodies 7
Supplementary Figure S4. Interaction of CD64-TPDCs with other immune cells 8
Supplementary Figure S5. Phenotypization of MDMs for CD64-TPDCs treatment 9
Supplementary Figure S6. Cytotoxic effect of CD64-TPDCs with Val-Cit-MMAE in MDMs 10
Supplementary Figure S7. Determination of CD64 expression in MDMs using CD64-TPDCs and CD64-mAb 11
Supplementary Figure S8. Detection of caspase-3/7 and -8 in MDMs induced by CD64-TPDCs 12
Supplementary Figure S9. Time-lapse microscopy of M2-like MDMs treated with CD64-TPDCs 13
Supplementary Figure S10. Internalization and colocalization of CD64-TPDCs within M1-like MDMs 14
Supplementary Figure S11. Internalization and colocalization of CD64-TPDCs within M2-like MDMs 15
Supplementary Figure S12. Internalization and colocalization of anti-CD64 antibody within MDMs 16
Supplementary Figure S13. Cathepsin K protein quantification in lysates of MDMs 17
Supplementary Table S1. Characterization of HPMA copolymer precursors 18
Supplementary Table S2. Characterization of CD64-TPDCs used in this study 19
Supplementary Table S3. Reagents table 20
Methods for synthesis of peptides 24
Methods for synthesis of peptide linkers with toxin moieties 27
Preparation of TPDCs 36
Chromatograms 48

Supplementary Figure S1. Chemical structures of targeted and functional moieties. (A) Representation of Val-Cit-PAB-MMAE, (B) Gly-Val-Cit-Gly-DM1 and (C) Gly-Phe-Leu-Gly-DM1 linkers with toxins used for conjugation to CD64-TPDCs. (D) Representation of cp33 cyclic peptide selectively targeting CD64.

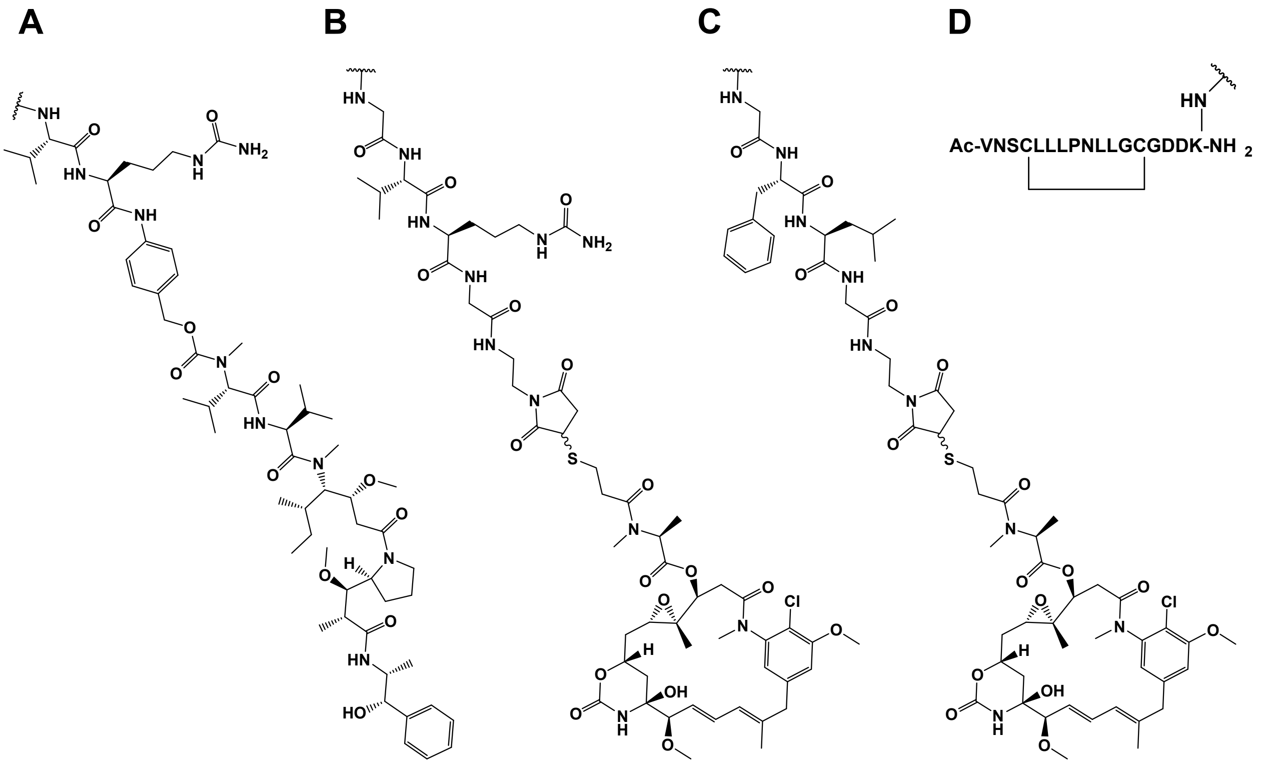

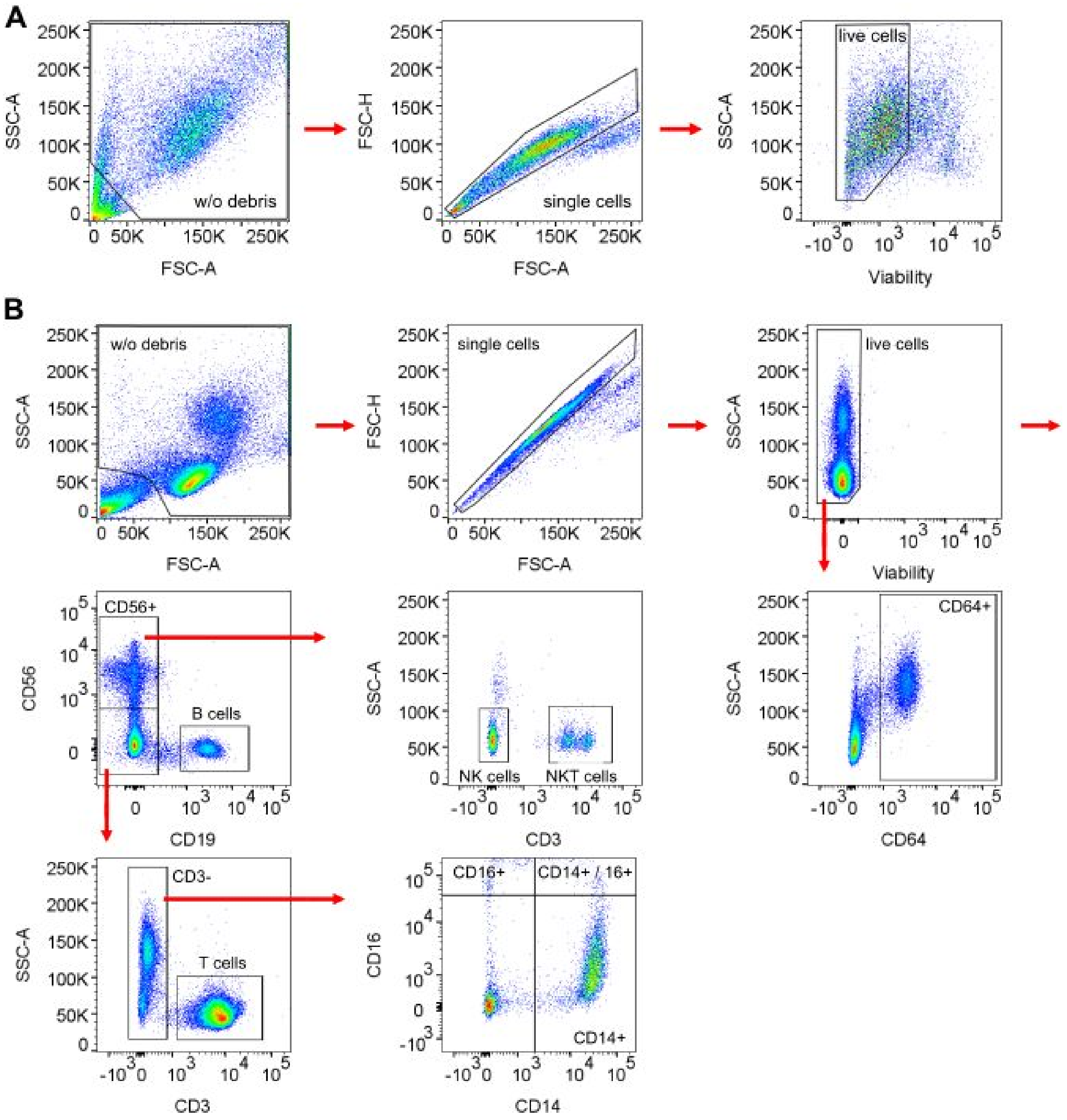

A
SSC-A
FSC-A
w/o debris
FSC-H
single cells
live cells
Viability
B
w/o debris
single cells
live cells
CD56+
B cells
CD56
CD19
NK cells
NKT cells
CD3
CD64+
CD64
CD3-
T cells
CD16+
CD14+ / 16+
CD14+
CD16
CD14

C

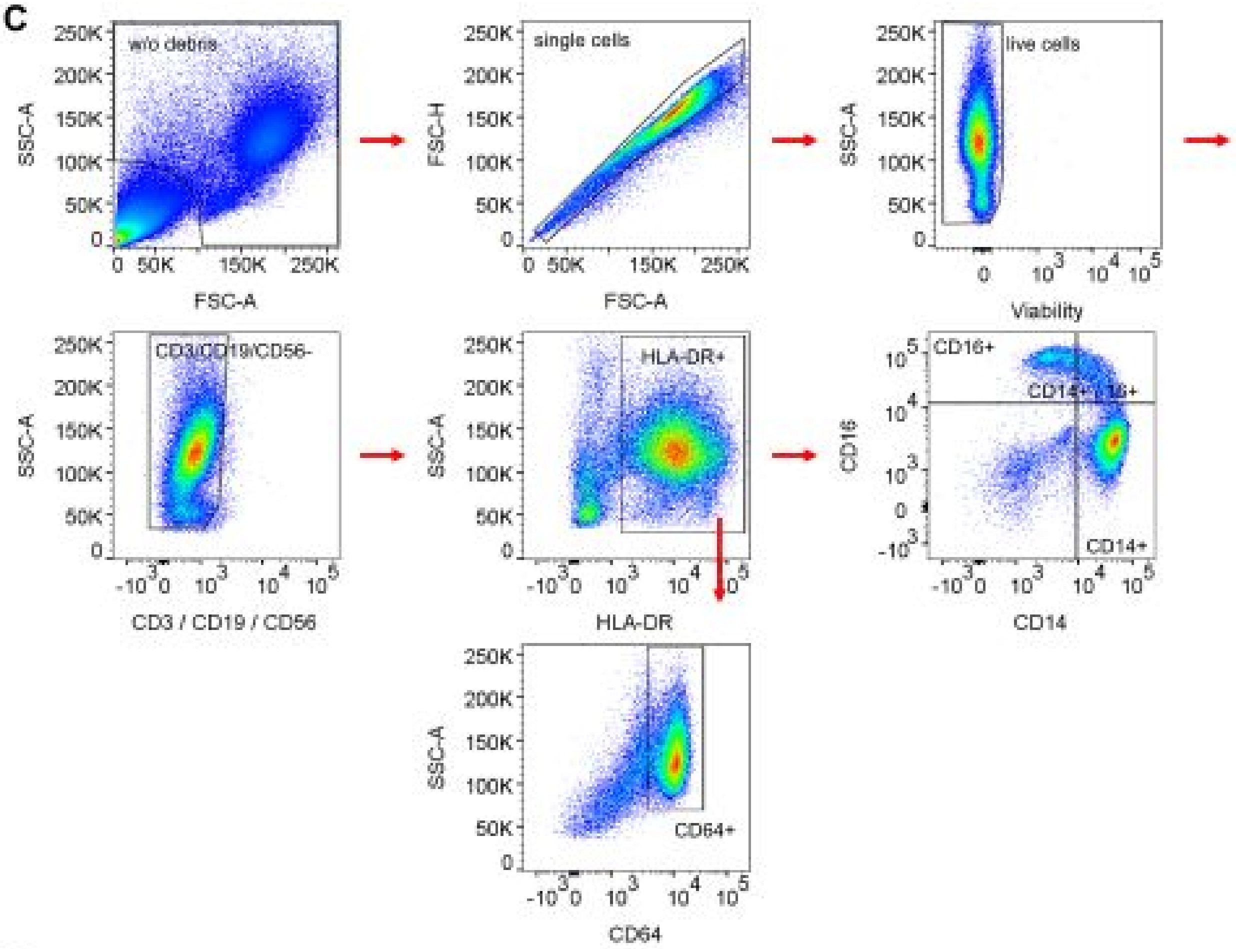

w/o debris
SSC-A
FSC-A
single cells
FSC-H
live cells
Viability
CD3/CD19/CD56-
CD3 / CD19 / CD56
HLA-DR+
HLA-DR
CD16+
CD14+ 16+
CD14+
CD16
CD14
CD64+
CD64

D

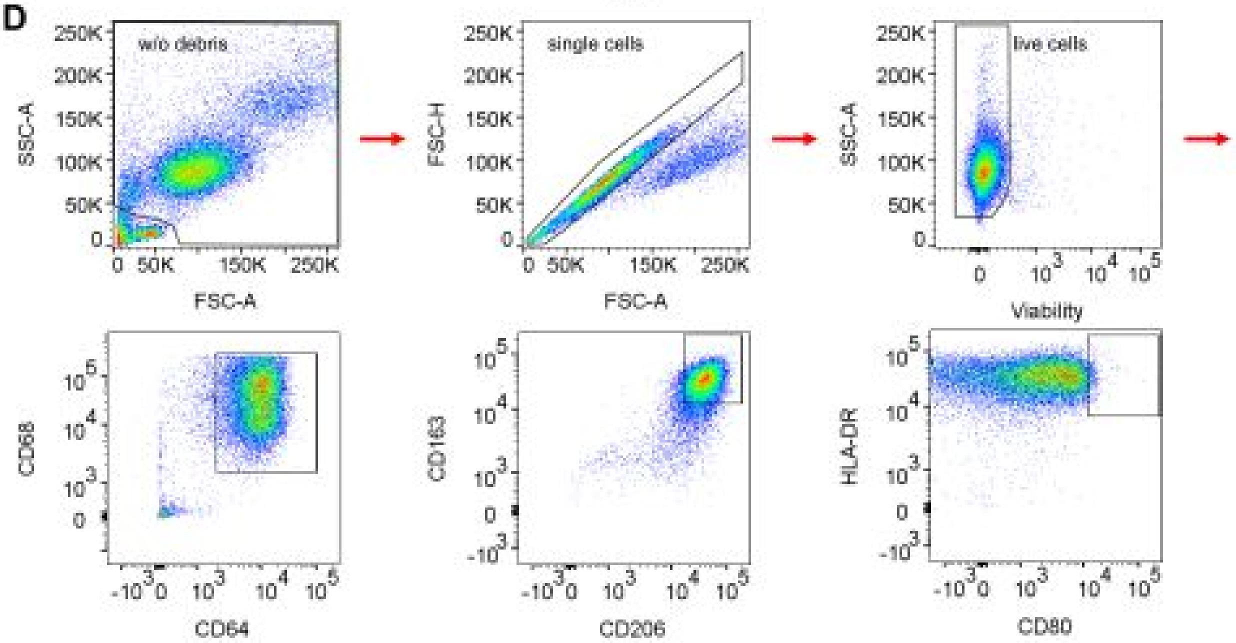

w/o debris
SSC-A
FSC-A
single cells
FSC-H
live cells
Viability
CD68
CD64
CD163
CD206
HLA-DR
CD80

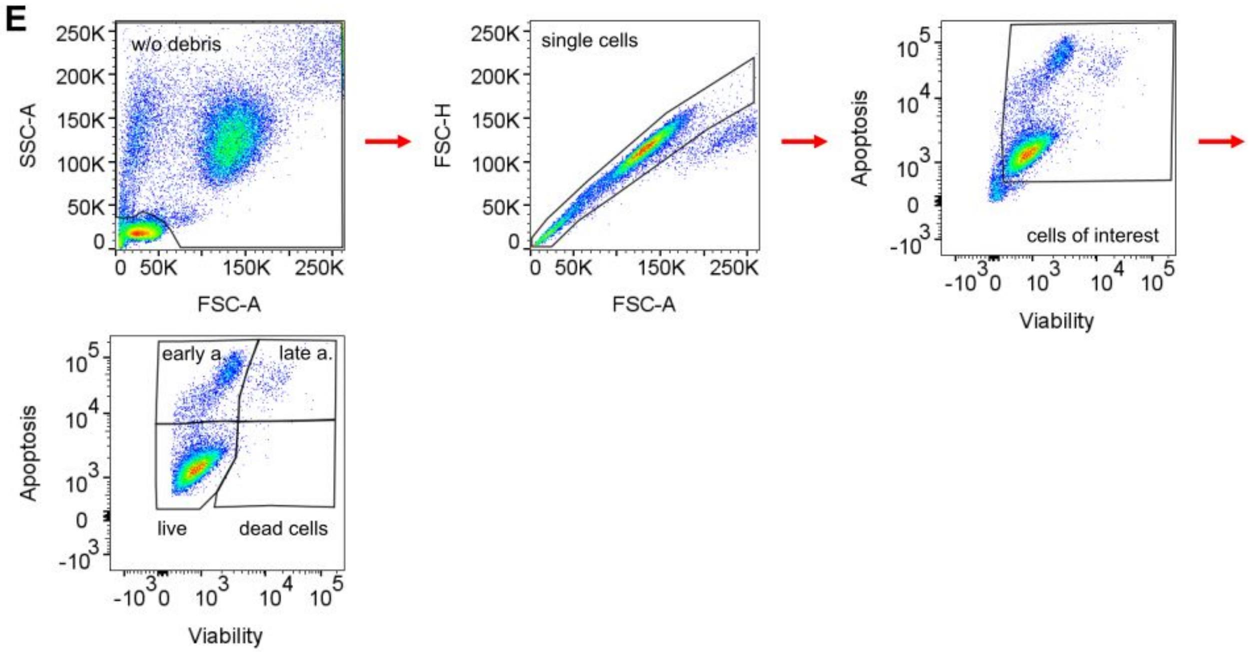

Supplementary Figure S2. Gating strategy for flow cytometry analysis. Samples were immunophenotyped using a BD LSRFortessa analyzer (FACS Diva software). For all gating strategies, debris was discerned using FSC-A/SSC-A followed by doublet exclusion using FSC-A/FSC-H gating parameters. (A) Gating strategy for Fig. 2A and 2B: Within the live-cell population, MFI of HEK cells with bound cp33-FAM or fC1 was identified (B) Panel 1 gating strategy – Immunophenotyping of isolated immune cells from peripheral blood: Within isolated PBMCs, monocytes, B, T, NKT, and NK cells were identified using CD3, CD19, CD56, CD14, CD16, and CD64 mAbs. (C) Panel 2 gating strategy – Immunophenotyping of isolated monocytes from peripheral blood: Within isolated PBMCs, monocytes were identified using HLA-DR, CD14, CD16, and CD64 mAbs. The purity of isolation was evaluated using CD3, CD19, and CD56 mAbs. (D) Panel 3 gating strategy – Immunophenotyping of MDMs: Within the live-cell populations, M1-like and M2-like MDMs were identified using CD14, CD64, CD68, CD80, HLA-DR, CD163, and CD206 mAbs. (E) Panel 4 gating strategy - live, dead, early (early a.) and late apoptotic (late a.) cells were identified within single cell population.

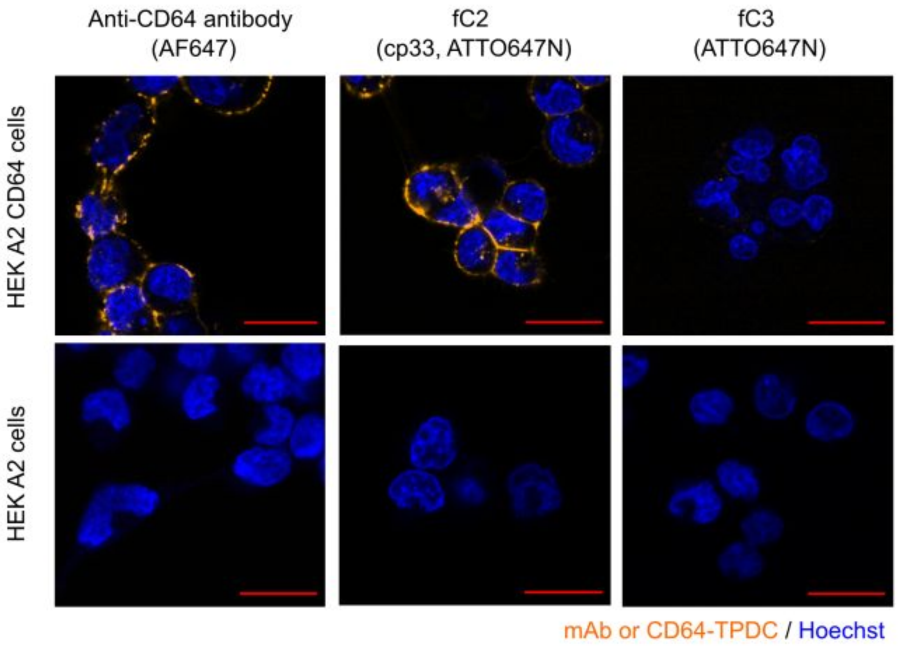

Supplementary Figure S3. CD64-TPDCs stain cells similarly to anti-CD64 antibodies. Confocal microscopy of the distribution of 200 nM **fC2**, **fC3**, or anti-CD64 mAb in PBS after 60 min incubation with HEK cells. Scale bars correspond to 20 µm. Data are representative of two independent experiments.

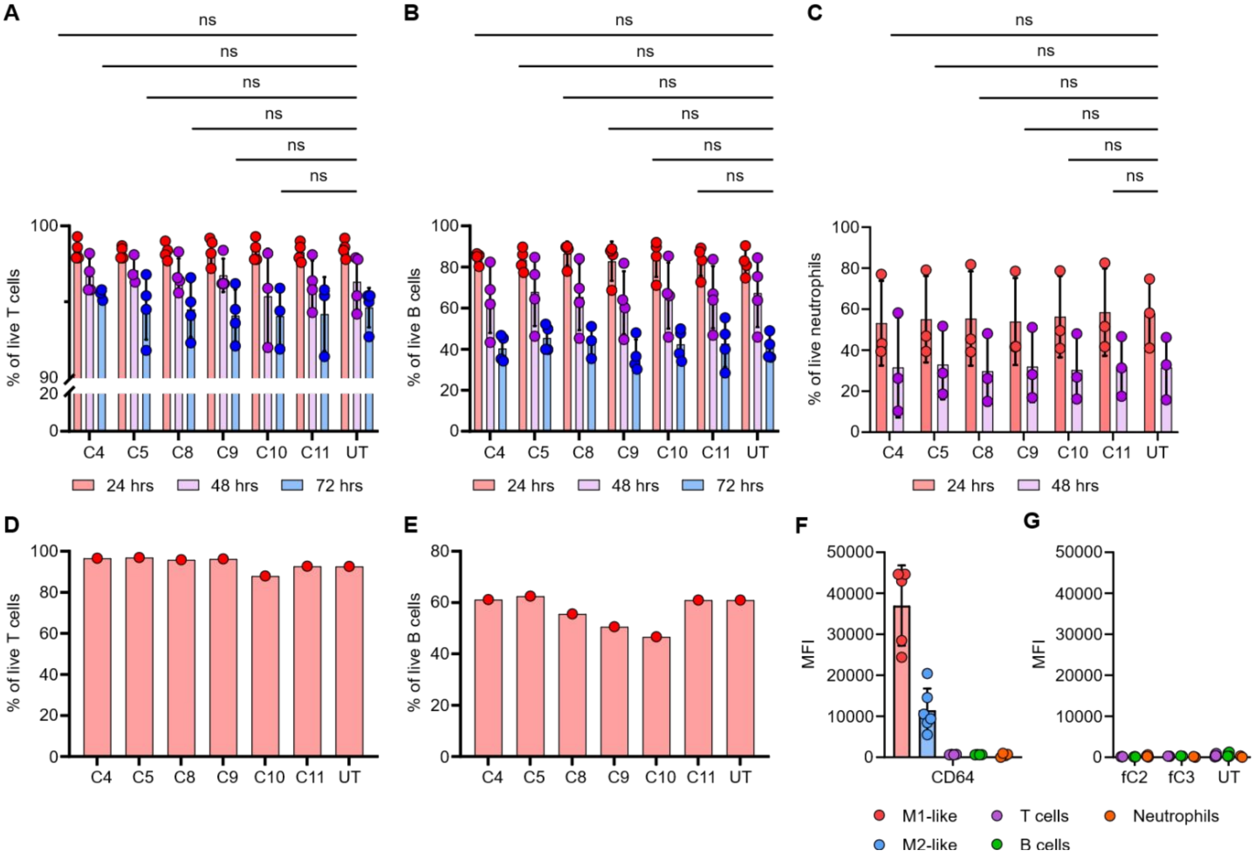

Supplementary Figure S4. Interaction of CD64-TPDCs with other immune cells. (A – E) T cells (A), B cells (B), and neutrophils (C) were incubated with 1 nM, or (D, E) with 1 μM **C4** (cp33, Val-Cit-PAB-MMAE), **C5** (Val-Cit-PAB-MMAE), **C8** (cp33, Gly-Phe-Leu-Gly-DM1), **C9** (Gly-Phe-Leu-Gly-DM1), **C10** (cp33), or **C11** (HPMA copolymer). Data are presented as mean ± SD from n = 4 PBMCs' donors. Statistical significance was determined using the Shapiro-Wilk test of normality and 1-way ANOVA followed by Dunn's multiple comparison test, with a 95% confidence interval. (F, G) CD64 expression detected by anti-CD64 AF647 mAb (F) and fluorescent CD64-TPDCs (G). Data are presented as mean ± SD from at least n = 4 PBMCs' donors. The associated gating strategy is provided in Supplementary Fig. S2.

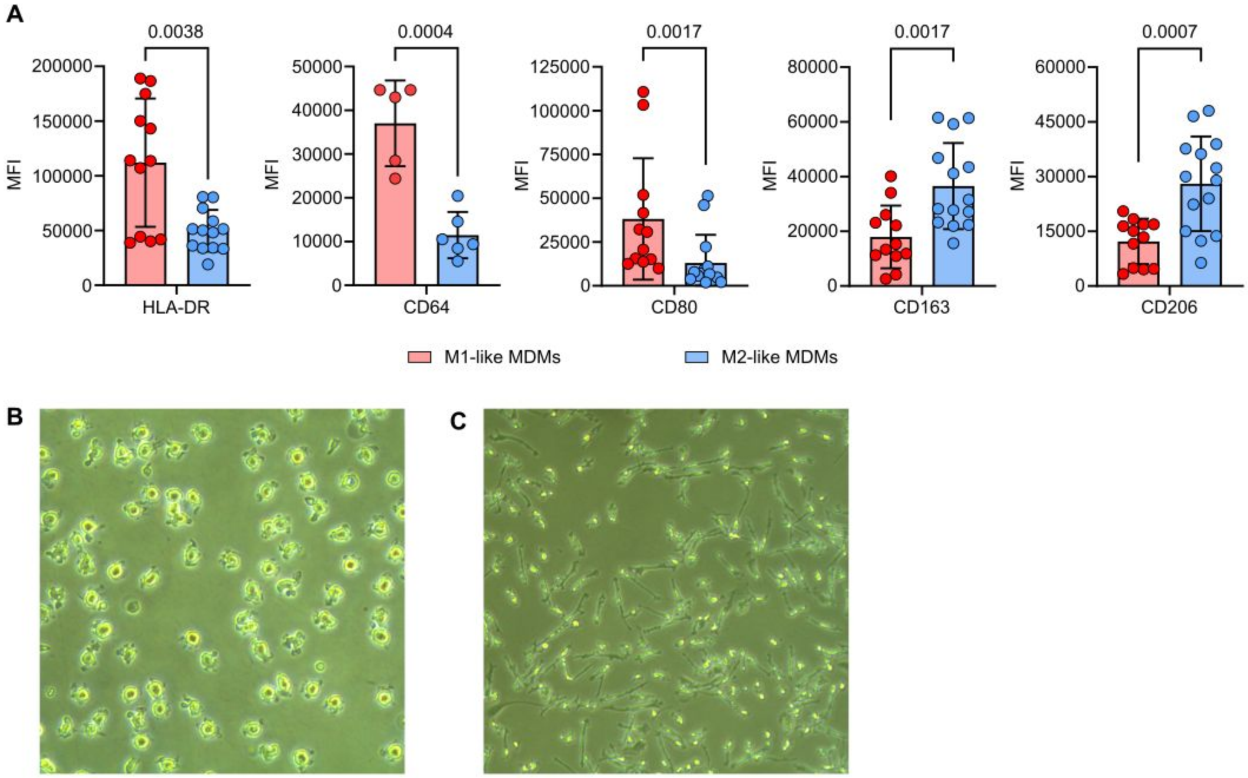

Supplementary Figure S5. Phenotypization of monocyte-derived macrophages for CD64-TPDCs treatment. (A) Expression of HLA-DR, CD64, CD80, CD163, and CD206 markers indicating the polarized state of MDMs. Data are presented as mean ± SD from five to ten independent experiments. Statistical significance was determined using the Welch's *t*-test (HLA-DR, CD206, and CD14) or Mann-Whitney *t*-test (CD80 and CD163). The gating strategy is provided in Supplementary Fig. S2. (B, C) Microscopy images of polarized M1-like (B) and M2-like MDMs (C). The picture was taken by a Nikon Eclipse TS100 light microscope.

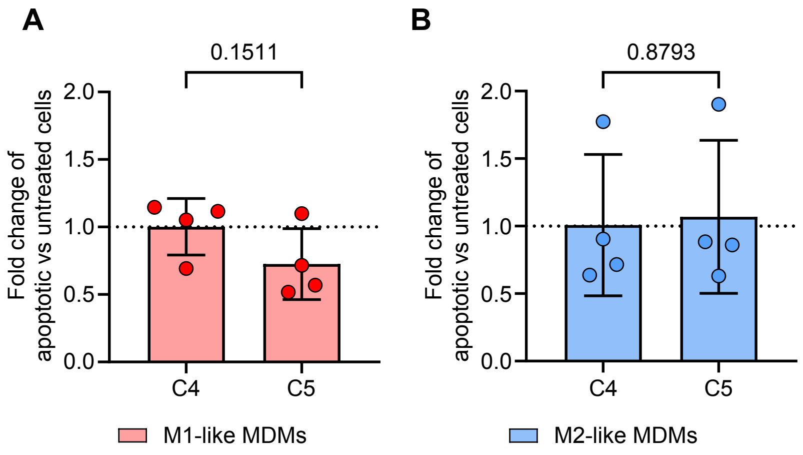

Supplementary Figure S6. Cytotoxic effect of CD64-TPDCs with Val-Cit-MMAE in MDMs. (A, B) Fully polarized M1-like (A) and M2-like (B) MDMs after 24-hour incubation with **C4** (cp33, Val-Cit-PAB-MMAE; 1 nM) and control **C5** (Val-Cit-PAB-MMAE; 1 nM). The plotted values represent the relative fold change in apoptotic macrophages in CD64-TPDCs-treated samples normalized to the corresponding untreated control within the same macrophage condition, indicated by the dotted line. Data are presented as mean ± SD from n = 4 MDMs samples. Statistical significance was determined using the Shapiro-Wilk test of normality and 2-way ANOVA, with a 95% confidence interval.

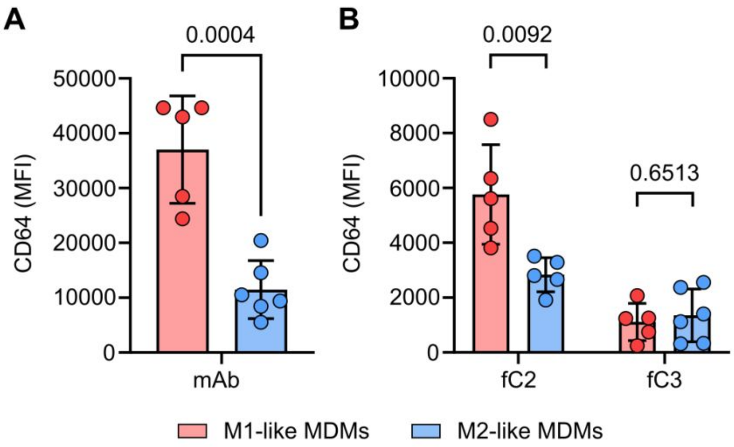

Supplementary Figure S7. Determination of CD64 expression in MDMs using CD64-TPDCs and CD64-mAb. (A) Binding of fluorescent anti-CD64 AF647 mAb and (B) **fC2** (cp33) and **fC3** TPDCs to M1-like and M2-like MDMs. Data are presented as mean ± SD at least *n* = 5 MDMs samples. Statistical significance was determined using the Shapiro-Wilk test of normality and 2-way ANOVA, with a 95% confidence interval. The gating strategy is provided in Supplementary Fig. S2.

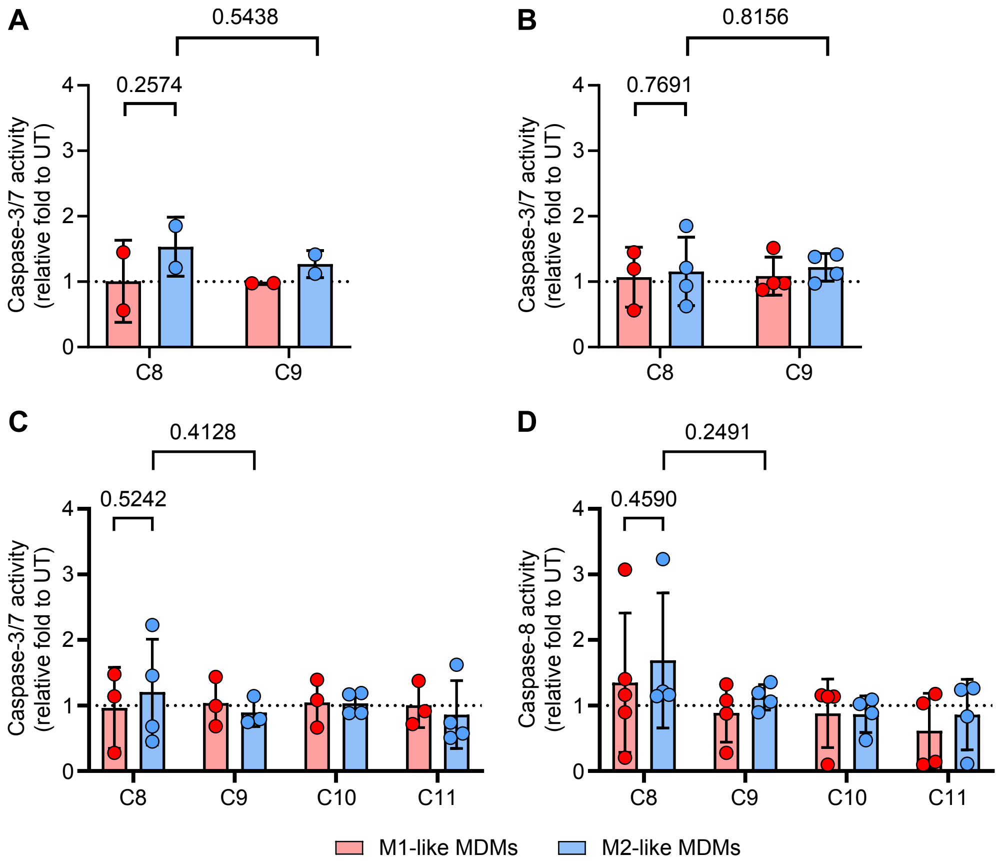

Supplementary Figure S8. Detection of caspase-3/7 and -8 in MDMs induced by CD64-TPDCs. (A) Caspase 3/7 in MDMs upon treatment with cytotoxic CD64-TPDCs (1 nM) for 8 hours, (B) 12 hours, and (C) and (D) caspase-8 for 24 hours, normalized to untreated cells (UT), indicated by the truncated line. Data are presented as mean ± SD from at least $n = 3$ MDMs samples. Statistical significance was determined using the Shapiro-Wilk test of normality and 2-way ANOVA followed by Fisher's LSD test, with a 95% confidence interval.

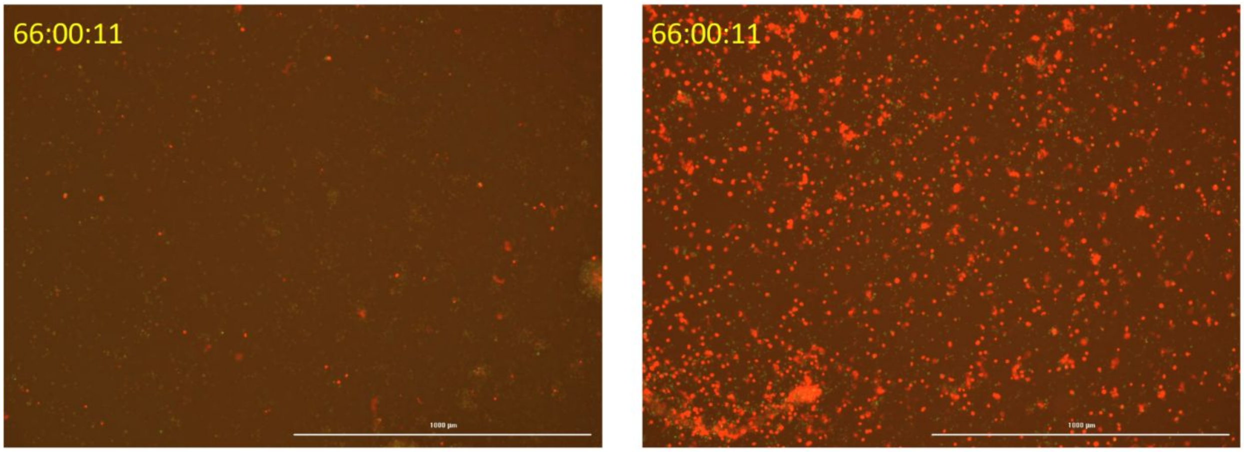

Supplementary Figure S9. Time-lapse microscopy of M2-like MDMs treated with CD64-TPDCs. M2-like MDMs were treated with (LEFT) the control cytotoxic **C9** (Gly-Phe-Leu-Gly-DM1; 1 nM) and (RIGHT) specific cytotoxic conjugate **C8** (cp33, Gly-Phe-Leu-Gly-DM1; 1 nM) for 66 hours. Red signals correspond to dead cells stained with propidium iodide, and green signals correspond to apoptotic cells stained with Apotracker Green. The scale bar represents 1000 µm.

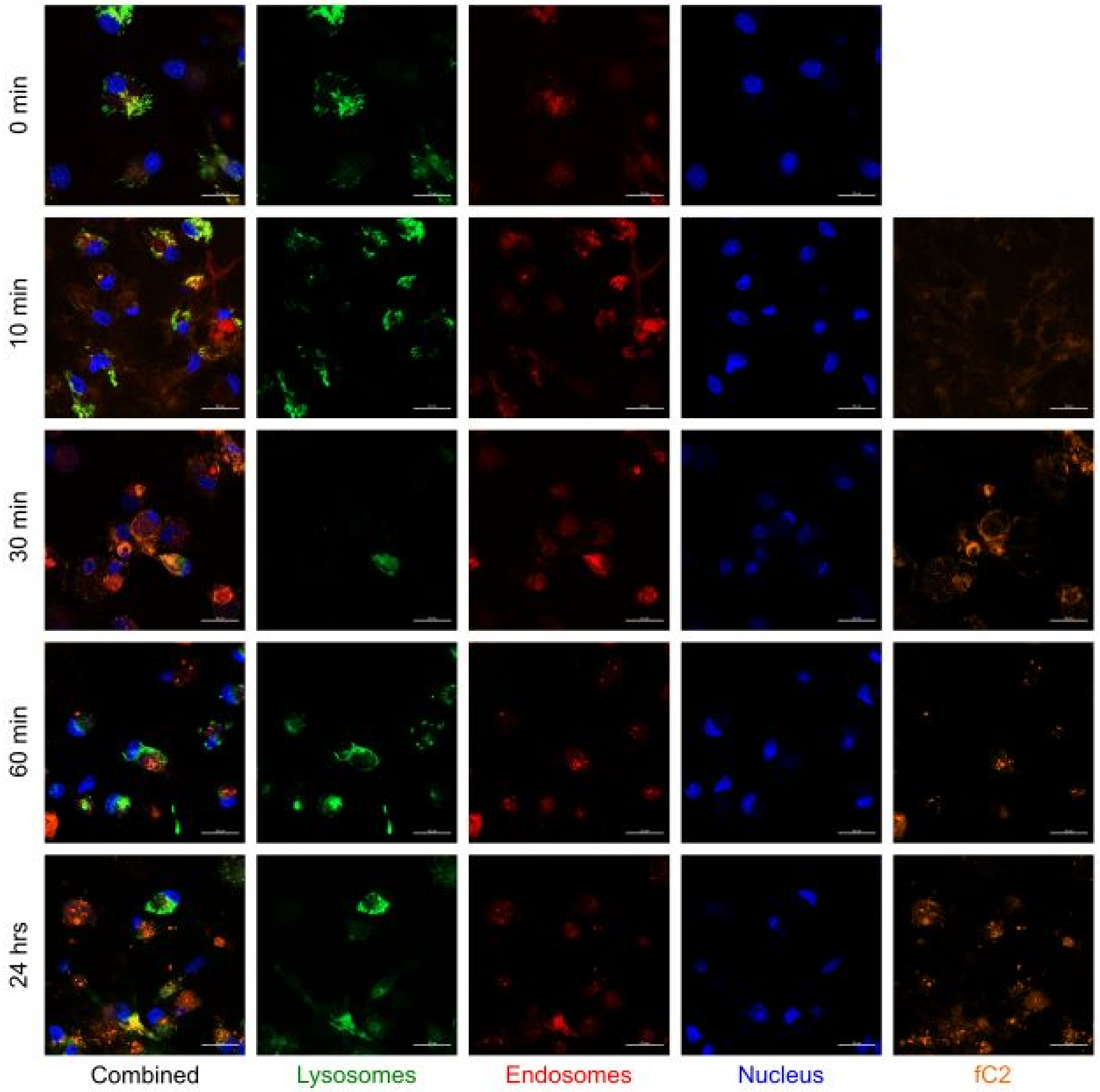

Supplementary Figure S10. Internalization and localization of CD64-TPDCs in M1-like MDMs. M1-like MDMs were stained with 200 nM conjugate **fC2** (oranget) for 10 minutes, 30 minutes, 60 minutes, and 24 hours with subsequent staining of lysosomes (green), late endosomes (red), and nuclei (blue). Scale bars correspond to 20 µm. Data are representative of three independent experiments from n = 3 MDMs samples.

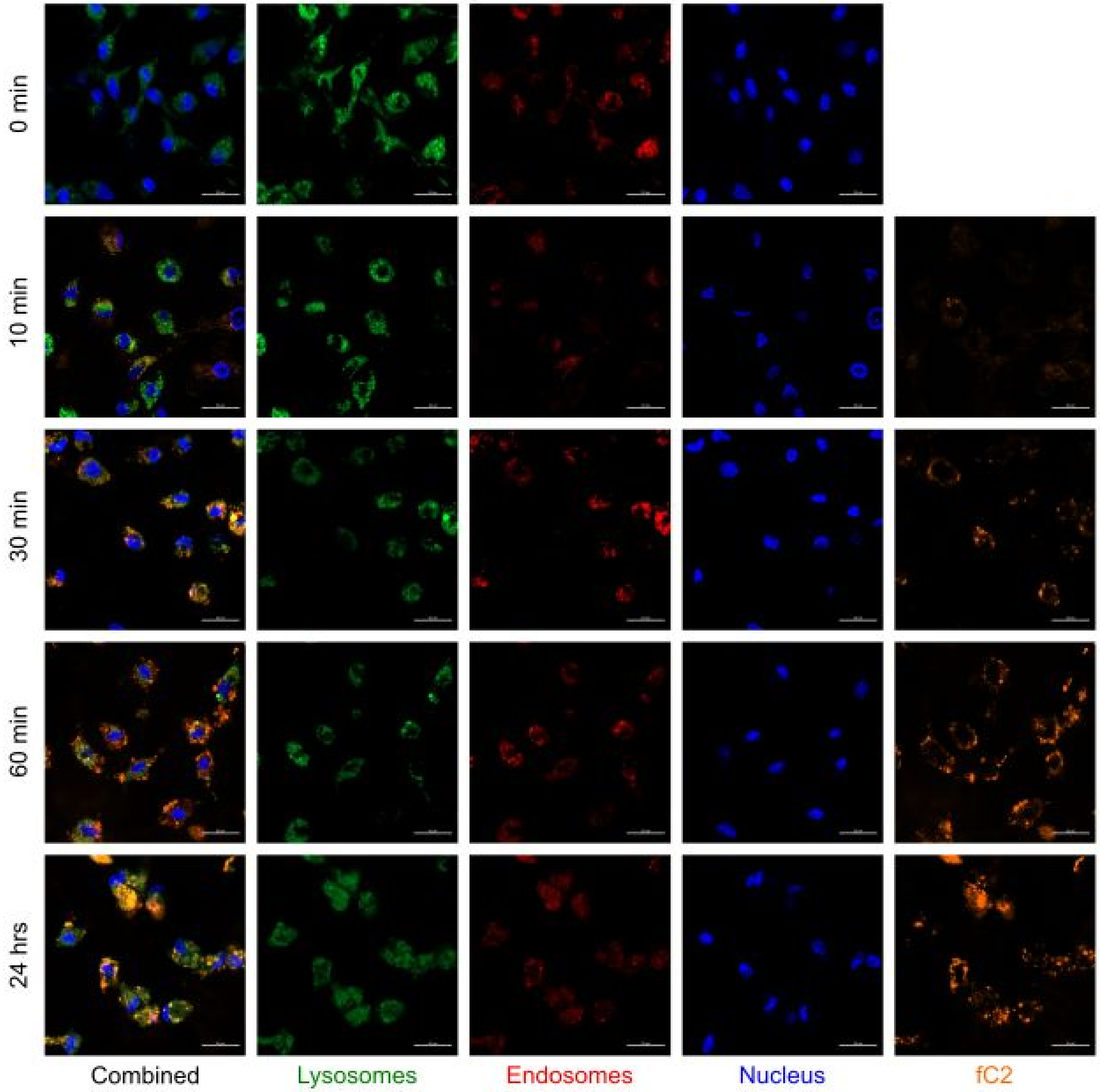

Supplementary Figure S11. Internalization and localization of CD64-TPDCs in M2-like MDMs. M2-like MDMs were stained with 200 nM conjugate **fC2** (oranget) for 10 minutes, 30 minutes, 60 minutes, and 24 hours with subsequent staining of lysosomes (green), late endosomes (red), and nuclei (blue). Scale bars correspond to 20 µm. Data are representative of three independent experiments from *n* = 3 MDMs samples.

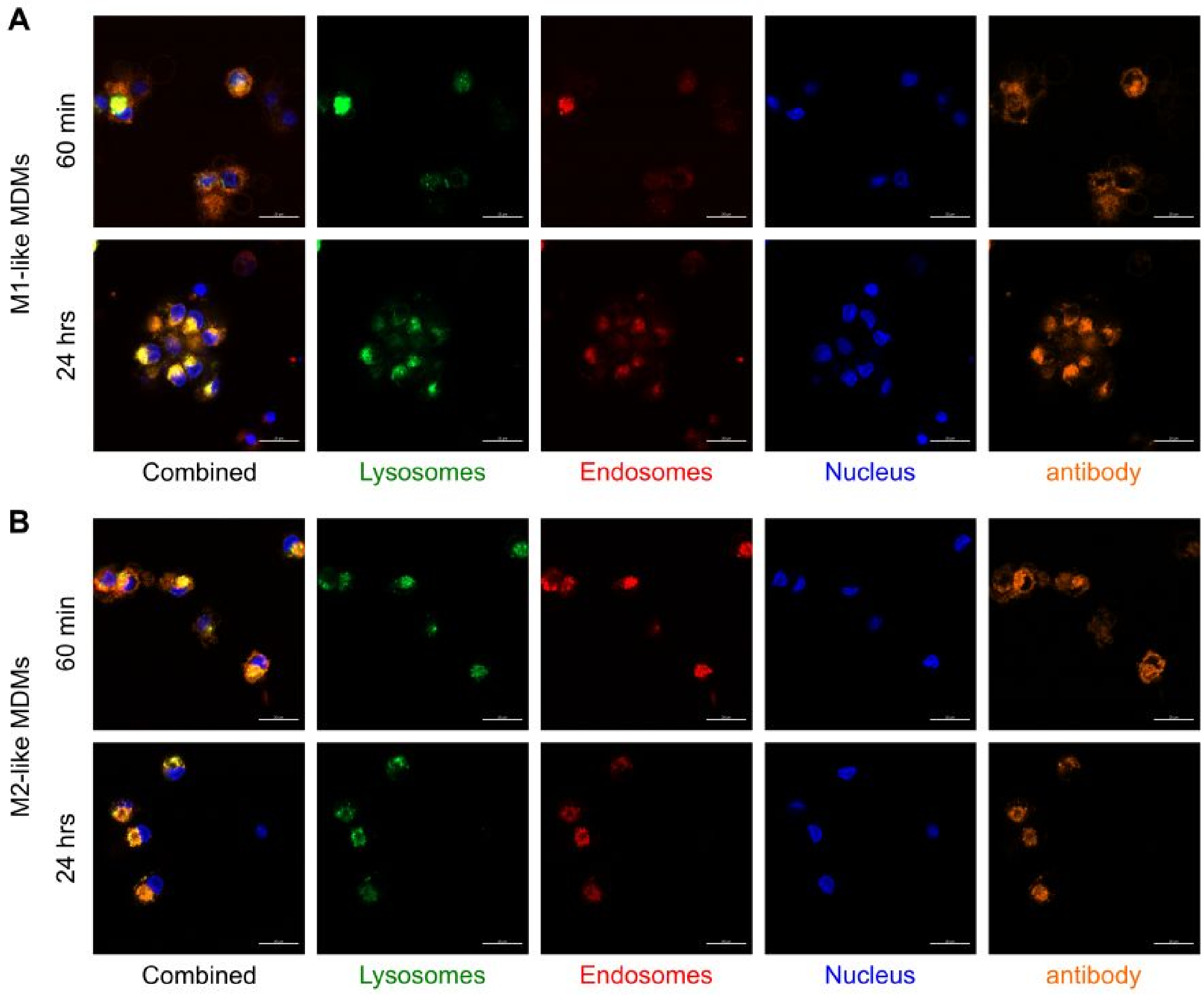

Supplementary Figure S12. Internalization and localization of anti-CD64 antibody in MDMs. (A) M1-like and (B) M2-like MDMs were stained with anti-CD64 monoclonal antibody conjugated with Alexa Fluor 647 (oranget) for 60 minutes, and 24 hours with subsequent staining of lysosomes (green), late endosomes (red), and nuclei (blue). Scale bars correspond to 20 µm. Data are representative of three independent experiments from *n* = 3 MDMs samples.

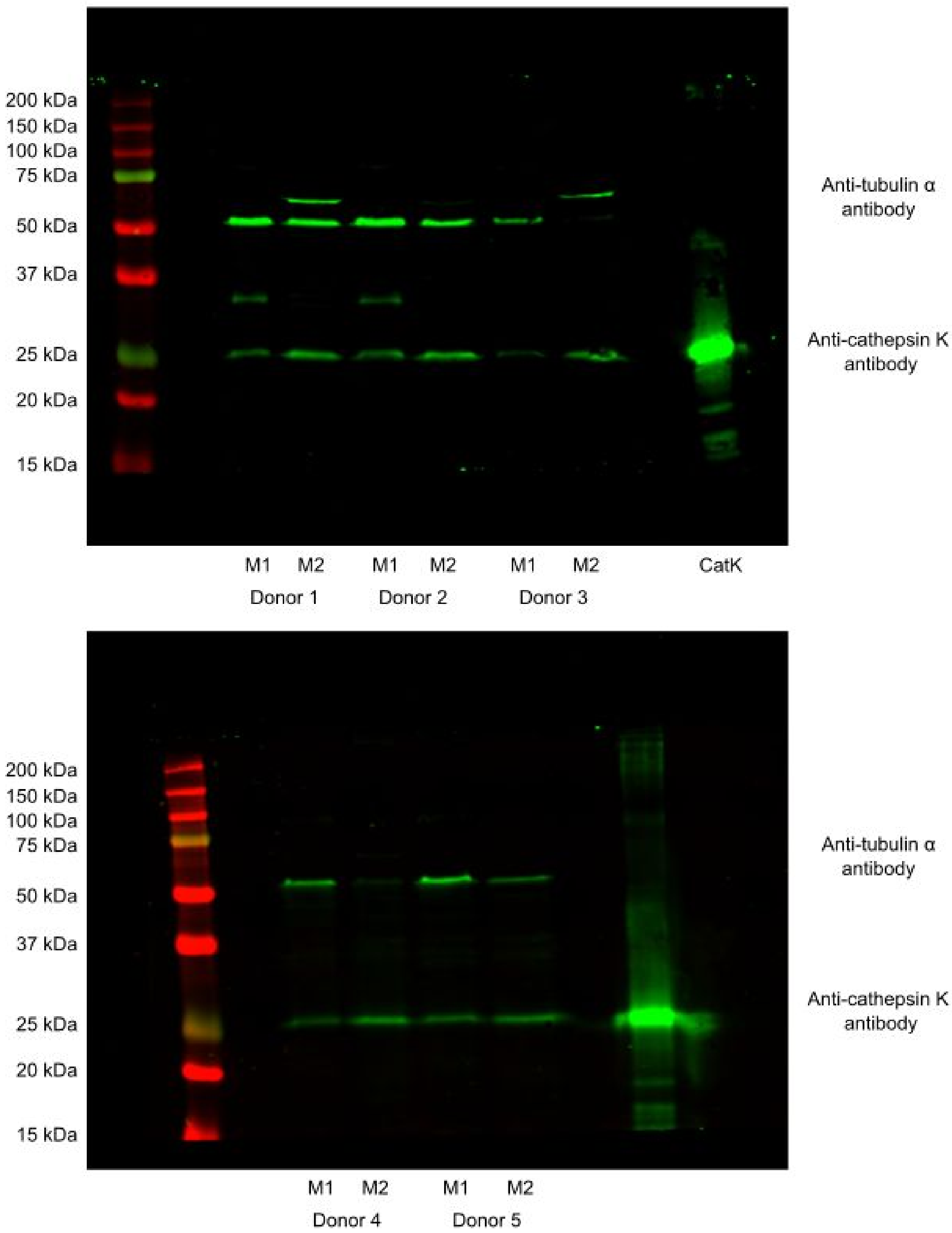

Supplementary Figure S13. Cathepsin K protein quantification in lysates of MDMs. As a positive control, CatK was used (200 ng). Molecular weights are indicated on the left. 100 ng of MDMs lysates were used. Tubulin α was used as a housekeeping protein. Data are presented from *n* = 5 MDMs donor samples.

Supplementary Table S1. Characterization of HPMA copolymer precursors. The table shows the average molecular weight M_w, the number average molecular weight M_n, the dispersity *Đ*, and the content of TT reactive groups.

Precursor	M_w (g.mol^{-1})	M_n (g.mol^{-1})	*Đ*	TT reactive groups (% mol)
P1	66 000	56 000	1.18	13.3
P2	64 000	58 000	1.10	10.3
P3	71 000	63 000	1.13	10.9
P4	72 000	64 000	1.13	10.0

Supplementary Table S2. Characteristics of TPDCs used in this study. The table shows the estimated molecular weight ($M_{w'}$) in kg.mol^{-1} of TPDCs, and the number of different moieties conjugated to the HPMA copolymer. As substituents, cp33 peptide, biotin, fluorophores ATTO488 or ATTO647N, toxins MMAE or DM1 with either Val-Cit-**PAB, Gly-Val-Cit-Gly, or** Gly-Phe-Leu-Gly cathepsins-cleavable linkers, and TrisNTA moiety were used.

**TPDCs**	**$M_{w'}$ (kg.mol^{-1})**	**Substituent 1 (# ligands/copolymer)**	**Substituent 2 (# ligands/copolymer)**	**Substituent 3 (# ligands/copolymer)**
fC1	84.5	cp33 (5.7)	ATTO488 (3.2)	-
fC2	104.2	cp33 (12.2)	ATTO647N (3.0)	biotin (18.1)
fC3	86.9	-	ATTO647N (2.3)	biotin (17.2)
C4	92.3	cp33 (9.8)	Val-Cit-PAB MMAE (7.6)	-
C5	72.1	-	Val-Cit-PAB MMAE (5.5)	-
C6	104.3	cp33 (13.9)	Gly-Val-Cit-Gly DM1 (10.4)	-
C7	79.1	-	Gly-Val-Cit-Gly DM1 (10.5)	-
C8	111.1	cp33 (16.0)	Gly-Phe-Leu-Gly DM1 (12.9)	-
C9	78.3	-	Gly-Phe-Leu-Gly DM1 (9.8)	-
C10	79.2	cp33 (4.8)	-	-
C11	65.0	-	-	-
C12	101.2	-	TrisNTA (17.2)	biotin (11.3)

Supplementary Table S3: Reagents table

REAGENT	SOURCE	IDENTIFIER
**Antibodies**		
Apotracker™ Green (FC 1:100)	Biolegend	#427403
AF647 anti-human CD64 (clone 10.1)(FC 1:100)	Exbio	#A6-644-T100
AF647 Mouse IgG1,κ Isotype control (clone MOPC-21)(FC 1:100)	Exbio	#A6-632-C025
APC anti-human CD19 (clone LT19)(FC 1:100)	Exbio	#1A-305-T100
APC Mouse IgG1,κ Isotype control (clone MOPC-21)(FC 1:100)	Exbio	#1A-632-C025
Anti-Cathepsin K antibody (IHC 1:1000)	Abcam	#ab187647
Anti-Tubulin αantibody (IHC 1:1000)	Merck	#SAB4500087
IRDye®800CW Goat anti-Rabit (IHC 1:15000)	LICORbio	#926-32211
BV421 anti-human CD14 (clone M5E2)(FC 1:100)	Biolegend	#301829
BV421 Mouse IgG1,κ Isotype control (clone MOPC-173)(FC 1:100)	Biolegend	#400259
BV510 anti-human CD163 (clone GHI/61)(FC 1:100)	BD OptiBuild™	#744921
BV510 Mouse IgG1,κ Isotype control (clone X40)(FC 1:100)	BD Horizon™	#562946
BV605 anti-human CD80 (clone L307.4)(FC 1:100)	BD Horizon™	#563315
BV605 Mouse IgG1,κ Isotype control (clone X40)(FC 1:100)	BD Horizon™	#562652
BV650 anti-human CD16 (clone 3G8)(FC 1:100)	Biolegend	#302041
BV650 Mouse IgG1,κ Isotype control (clone MOPC-21)(FC 1:100)	Biolegend	#400163
BUV395 anti-human CD3 (clone UCHT1)(FC 1:100)	BD Horizon™	#563548
BUV395 anti-human CD206 (clone 19.2)(FC 1:100)	BD OptiBuild™	#740309
BUV395 Mouse IgG1,κ Isotype control (clone X40)(FC 1:100)	BD Horizon™	#563547
BUV395 anti-human CD19 (clone SJ25C1)(FC 1:100)	BD Horizon™	#563551
BUV395 anti-human CD56 (clone NCAM16)(FC 1:100)	BD Horizon™	#563555
BUV395 Mouse IgG2b,κ Isotype control (clone 27-35)(FC 1:100)	BD Horizon™	#563558
FITC anti-human CD11b (clone ICRF44) (FC 1:100)	BD Pharmingen™	#562793
FITC Mouse IgG1,κ Isotype control (clone MOPC-21)(FC 1:100)	BD Pharmingen™	#555748
FITC anti-human CD64 (clone 10.1)(FC 1:100)	BD Pharmingen™	#555527
FITC Mouse IgG1,κ Isotype control (clone MOPC-21)(FC 1:100)	BD Pharmingen™	#563547
PE anti-human CD56 (clone B159)(FC 1:100)	BD Pharmingen™	#561903
PE Mouse IgG1,κ Isotype control (clone B159)(FC 1:100)	BD Pharmingen™	#561903
PE anti-human CD68 (clone Y1/82A)(FC 1:100)	Exbio	#1P-749-T025
PE Mouse IgG2b,κ Isotype control (clone MPC-11)(FC 1:100)	Exbio	#1P-692-C100
PE-CF594 anti-human HLA-DR (clone G46-6)(FC 1:100)	BD Horizon™	#562304
PE Mouse IgG2a,κ Isotype control (clone G155-178)(FC 1:100)	BD Horizon™	#562306
Human TruStain FcX block	Biolegend	#422302

Zombie NIR fixable viability kit	Biolegend	#423106
**Critical commercial assays**		
Blocker™ Casein	Thermo Scientific™	#37532
Bradford assay		
CellTiter-Glo Luminescent Cell Viability Assay	Promega	#G7570
Caspase-Glo® 3/7 assay	Promega	#G8091
Caspase-Glo® 8 assay	Promega	#G8201
FuGENE® HD transfection reagent	Promega	#E2311
HIS-Select® Nickel Affinity Gel	Sigma-Aldrich	#P6611
Lymphoprep	StemCell™	#07851
RosetteSep™ Human Monocyte Enrichment cocktail	StemCell™	#15068
RosetteSep™ Human T Cell Enrichment cocktail	StemCell™	#15061
RosetteSep™ Human B Cell Enrichment cocktail	StemCell™	#15064
RosetteSep™ Human NK Cell Enrichment cocktail	StemCell™	#15065
RosetteSep™ Direct Human Neutrophil isolation kit	StemCell™	#19666
**Experimental models: Cell Lines**		
Hek 293	Prof. Hořejší	-
HEK293-pTetOff-A2	in this study	HEK with pTetOff
HEK293-pTetOff-A2 CD64	in this study	HEK with inducible CD64
**Software and algorithms**		
Astra 8.1.2	Waters/Wyatt	-
Astra V	Waters/Wyatt	-
BD FACSDiva™ Software v9.0	BD	-
BioRender	BioRender	-
GraphPad (10.3.0)	Dotmatics	-
FlowJo™ (v10.10.0)	FlowJo	-
CarlZeiss AG, ZEN (v3.5 blue edition)	Zeiss Microscopy	-
ChemDraw (v21.0.0)	Revvity signals	-
Clarity (v8.8)	DataApex	-
Excel (v2310)	Microsoft	-
Inkscape (v1.3.2)	Inkscape	-
LCsolution (v1.25 SP2)	Shimadzu	-
MestReNova (v12.0)	Mestrelab Research	-
Prism (v10.1.2)	GraphPad	-
SPR UP (v1.1.12.56)	UFE, CAS, CZE	-

TraceDrawer (v1.5)	TraceDrawer	-
WinASPECT (v2.5.0.0)	Jena Analytics	-
**Reagents**		
Amicon® ultracentrifugal filter 10kDa MWCO Nickel Affinity Gel	Merck	#UFC5010
Amicon® ultracentrifugal filter 30kDa MWCO Nickel Affinity Gel	Merck	#UFC5030
bovine serum albumin	Sigma	#A7906
DM1	MedChemExpress	#HY-19792
DAPI	Merck	#268298
fetal bovine serum	Sigma-Aldrich	#F7524
Ficoll	Cytiva	#17144002
fluorescent hydrolysis probe #87	Roche Universal Probe Library	#04689127001
G-418 Solution (geneticin)	Roche	#04727878001
Hoechst 34580	Sigma-Aldrich	#63493
IMDM (1x)	Gibco	#21056-023
Immersol Immersion Oil	Carl Zeiss Microscopy	#518 F
L-Glutamine	Merck	#G7513
oligonucleotides	Generi-Biotech	Custom synthesis
Puromycin dihydrochloride	Sigma	#P7255
pPUR vector	Clontech	#631601
pTet-Off® Advanced vector	Clontech	#630934
RPMI medium 1640 (1x)	Gibco	#32404-014
Spherotech fluorescent beads	Spherotech	#ACFP-70-10
SuperBlock® solution	Thermo Scientific™	#37581
**Chemicals**		
All chemicals and solvents were of analytical grade		
Acutase	Merck	#A6964
β-Alanine	Merck	#05159
1-Amino-propan-2-ol	TCI	#A1229
1-(2-aminoethyl)-1*H*-pyrrole-2,5-dione hydrochloride	BroadPharm	#BP-20991
ATTO488-amine	ATTO-TEC	#AD-488-95
ATTO647N-amine	ATTO-TEC	#AD-647N-95
2,2′-azobis(4-methoxy-2,4-dimethylvaleronitrile)	Wako Chemicals	#926-10500
N-Biotinyl-ethylenediamine trifluoroacetate salt	Merck	#08599
Bromophenol blue	Thermo Scientific™	#A18469.09

tert-Butanol	Thermo Scientific™	#447010010
Carbon disulfide	Sigma-Aldrich	#180173
Copper(II) sulfate	Sigma-Aldrich	#451657
N,*N*-Diisopropylethylamine	Sigma-Aldrich	#D125806
N,*N*-Dimethyl acetamide	Thermo Scientific™	#396351000
Dimethyl sulfoxide	VWR Chemicals	#23488.294
doxocycline hyclate	Merck	#D9891-25G
Ethanethiol	Merck	#E3708
Ethanolamine	Merck	#E9508
N-Ethyl-*N′*-(3-dimethylaminopropyl)carbodiimide hydrochloride	Merck	#03450
HABA/Avidin Reagent	Merck	#H2153
HS-(CH2)11-(CH2CH2O)6-OCH2-COOH	Prochimia	#TH 001-m11.n4-0.2
HS-(CH2)11-(CH2CH2O)4-OH HS-C11-(EG)4-OH	Prochimia	#TH 003-m11.n6-0.1
Isopropylmagnesium chloride (2M solution in THF)	Merck	#230111
Methacryloyl chloride	Merck	#523216
Methanol	Merck	#1060351000
β-mercaptoethanol	Merck	#M3148-25ML
NH_2-$(PEG)_{11}$-biotin	BroadPharm	#BP-21623
Neutravidin	Thermo Scientific™	#31000
N-hydroxysuccinimide/(1-ethyl-3-(3dimethylaminopropyl)carboiimide, Amine Coupling Kit	Cytiva	#BR 1000-50
PD10 columns	Cytiva	#17085101
Potassium chloride	Lach-ner	#61012
Potassium phosphate	Lach-ner	#30016
Propidium iodide	Biolegend	#421301
Sephadex LH-20	Cytiva	# 17009003
Sodium acetate	Penta	#22740-31000
SDS	Merck	#8170341000
Sodium chloride	Lach-ner	#61013
Sodium hydride (60% dispersion in mineral oil)	Aldrich	#45291-2
Sodium phosphate	Lach-ner	#30388
2-Thiazoline-2-thiol	Merck	#M6204
Trifluoroacetic acid	IrisBiotech	#SOL-011.0500
Tris	Promega	#H5133
Tween20	Thermo Scientific™	#J20605
UV ethanol	Penta	#70391-11001

Synthesis of small molecules

I. Methods for synthesis of peptides

Synthesis of the cp33 peptide

The anti-CD64 cp33 peptide (Ac-VNSCLLLPNLLGCGDDK-NH_2) was synthesized by solid-phase peptide synthesis (SPPS) using the Liberty Blue peptide synthesizer (CEM, USA) with standard Fmoc chemistry protocols, DIC/Oxyma-Pure coupling reagents and the Rink amide MBHA resin support (0.2 mmol scale, 10 equiv. amino acid excess). Fmoc groups were removed with 20% piperidine. The side chains of peptide amino acids were deprotected, and the peptide was cleaved off the resin with a mixture of TFA/Thioanisole/EDT/Anisole (90:5:3:2) at room temperature (RT) for 90 minutes. The cleaved peptide was then lyophilized and purified by reversed-phase high-performance liquid chromatography (RP HPLC; Vydac 218TP101522 column) using methanol and water with 0.05% TFA as solvents. Purity was assessed by analytical RP HPLC (Vydac 218TP54 column) and LCMS (Agilent Technologies 6230 ToF LCMS): Ac-VNSCLLLPNLLGCGDDK-NH_2 $[M+2H]^{2+}$ 907.9.

The cp33 peptide cyclization

The peptide was dissolved in 0.1 M aqueous ammonium acetate buffer, and the mixture was stirred overnight at RT. Subsequently, the reaction mixture was lyophilized. The cyclized peptide was then purified by RP HPLC, and its purity was assessed as described above: S-S cyclized Ac-VNSCLLLPNLLGCGDDK-NH_2

LRMS (ESI): m/z calculated for $C_{77}H_{130}N_{21}O_{25}S_2$ $[M+H]^+$ 1812.9, found: 1812.9.
HRMS (ESI): m/z calculated for $C_{77}H_{130}N_{21}O_{25}S_2$ $[M+H]^+$ 1812.89827, found: 1812.89886.

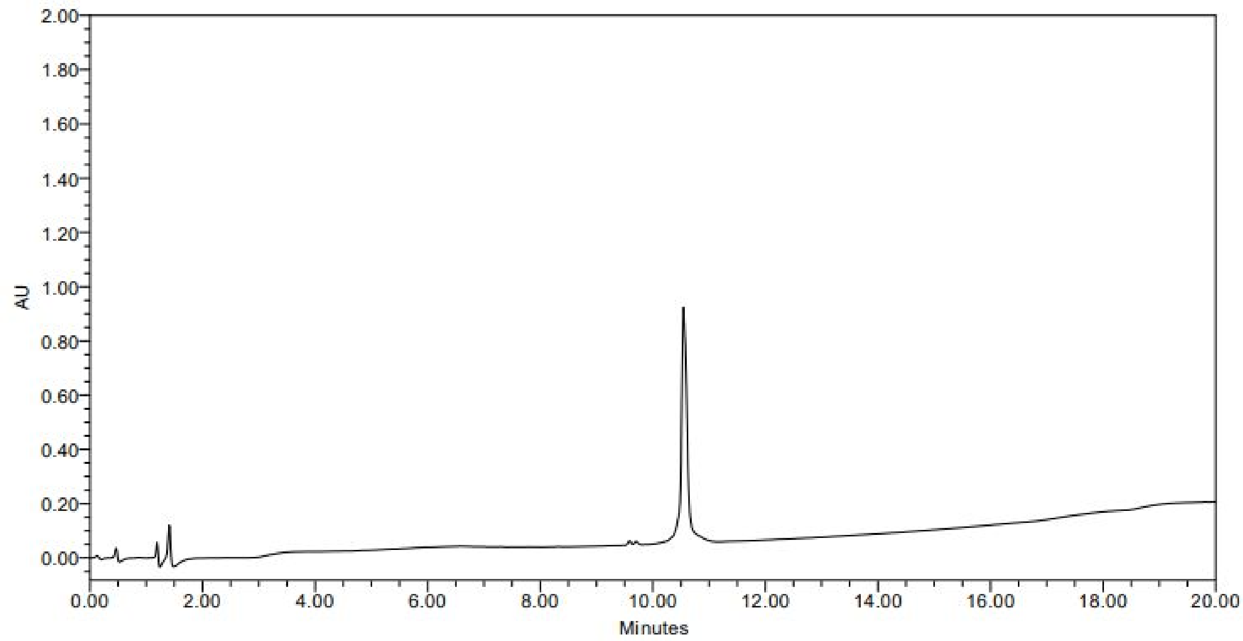

AU
Minutes
2.00
1.80
1.60
1.40
1.20
1.00
0.80
0.60
0.40
0.20
0.00
0.00
2.00
4.00
6.00
8.00
10.00
12.00
14.00
16.00
18.00
20.00

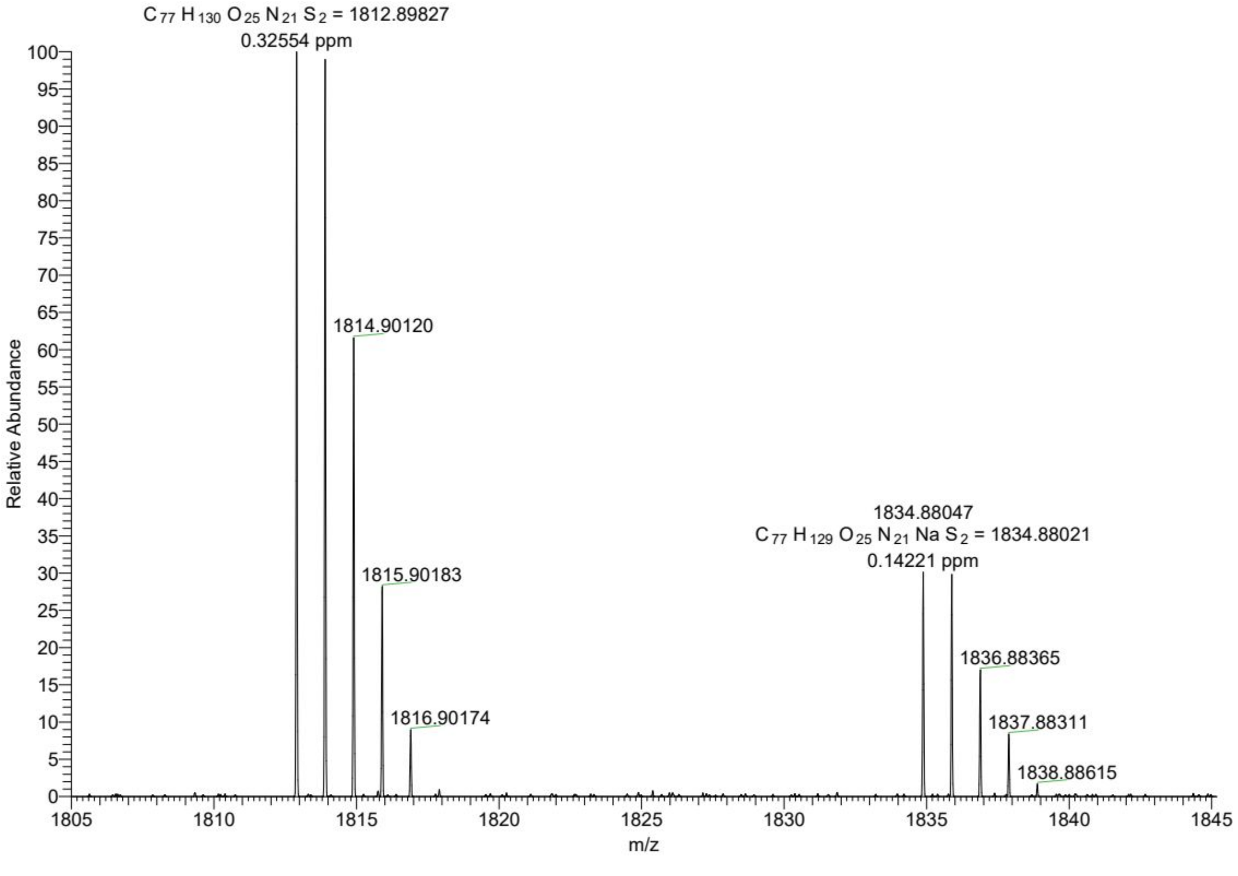

C 77 H 130 O 25 N 21 S 2 = 1812.89827
0.32554 ppm
1814.90120
1815.90183
1816.90174
1834.88047
C 77 H 129 O 25 N 21 Na S 2 = 1834.88021
0.14221 ppm
1836.88365
1837.88311
1838.88615
Relative Abundance
m/z
1805
1810
1815
1820
1825
1830
1835
1840
1845

Labeling of cp33 with 5(6)-Carboxyfluorescein

5(6)-Carboxyfluorescein (FAM) was activated with 1 equiv. HOSu and 5 equiv. DIC in DMF for 2 hours. The solvent and excess DIC were removed using a vacuum oil pump overnight. The cyclic peptide was incubated in DMF with 5 equiv. of 5(6)-Carboxyfluorescein-OSu ester in the presence of 3 equiv. DIPEA overnight. The reaction mixture was evaporated. The fluoresceinylated cyclic peptide was purified by RP HPLC and its purity was assessed as described above. The mass was confirmed by MALDI-ToF MS: S-S cyclized Ac-VNSCLLLPNLLGCGDDK(5(6)-Carboxyfluorescein)-NH_2

LRMS (ESI): m/z calculated for $C_{98}H_{141}N_{21}O_{31}S_2$ $[M+2H]^{2+}$ 1086.0, found: 1086.5.
HRMS (ESI): m/z calculated for $C_{98}H_{141}N_{21}O_{31}S_2$ $[M+2H]^{2+}$ 1085.97664, found: 1085.97711.

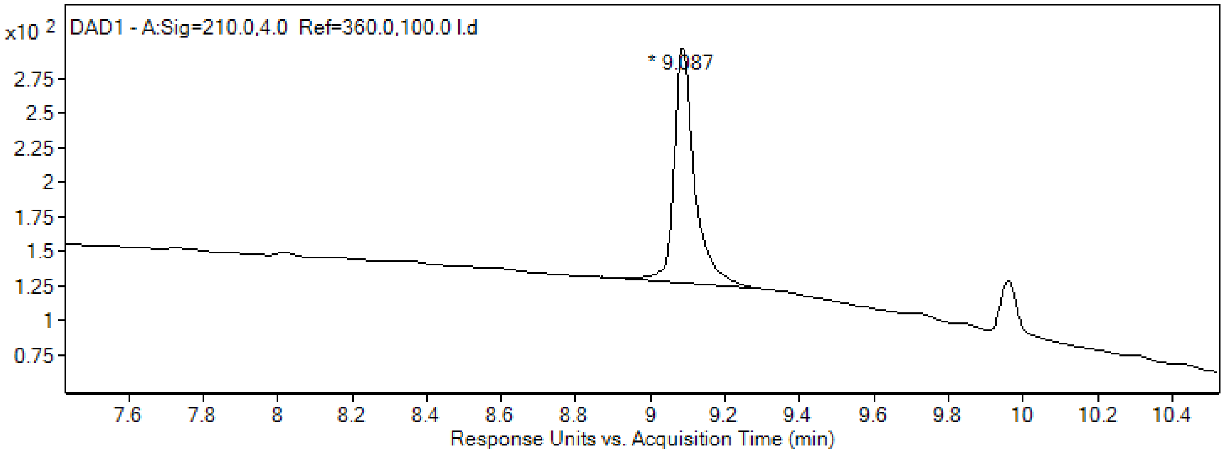

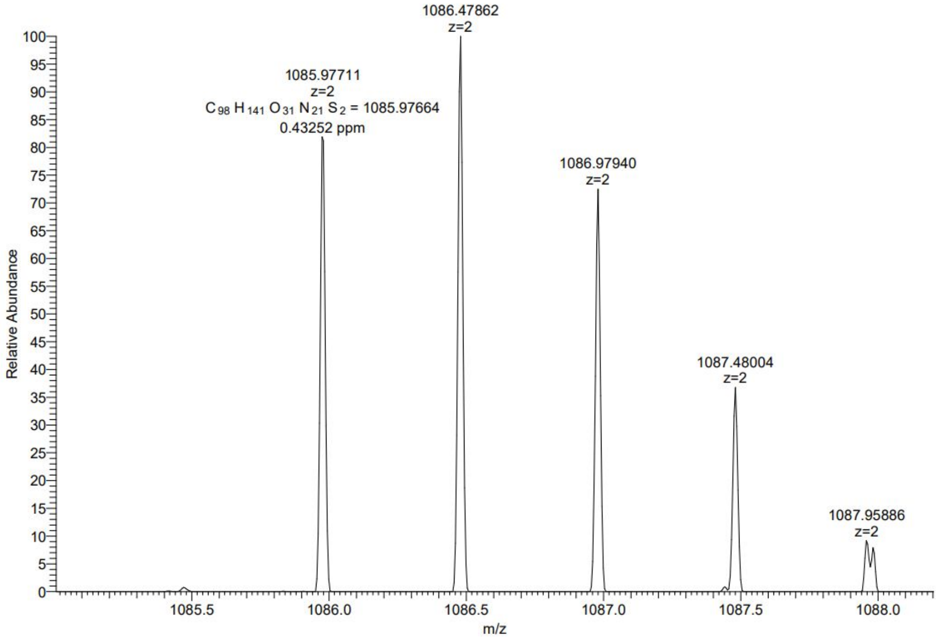

II. Methods for synthesis of peptide linkers with toxin moieties

General procedure for manual solid-phase peptide synthesis

The 2-Chlorotrityl chloride resin (200 – 400 mesh) 1.60 mmol/g was allowed to swell in anhydrous dichloromethane (DCM) for 10 minutes. A solution of Fmoc-Gly-OH (0.7 eq.) and *N*,*N*-diisopropylethylamine (3 eq.) in anhydrous DCM was added to the resin, and the mixture was shaken for 16 hours. Then, the resin was washed with *N*,*N*-dimethylformamide (DMF) (2×), DCM (2×) and DMF (3×). The Fmoc group was removed by treatment with 20% piperidine in DMF (1 × 5 min, 1 × 30 min). The resin was washed with DMF (3×), 2-propanol (2×) and DCM (3×).

The peptide chain was extended following the general procedure for coupling of Fmoc-AA-OH using amino acids in the desired order. The typical procedure involved adding a solution of Fmoc-AA-OH (3 eq.), HATU (3 eq.) and 2,4,6-collidine (6 eq.) in anhydrous DMF to the resin, and shaking the mixture for 5 hours. Subsequently, the resin was washed with DMF (2×), DCM (2×) and DMF (3×). The Fmoc group was removed by treatment with 20% piperidine in DMF (1 × 5 min, 1 × 30 min). The resin was washed with DMF (3×), 2-propanol (2×) and DCM (3×), and the coupling step was repeated. In the last coupling step, Boc-Gly-OH was used instead of the standard Fmoc-AA-OH. Then, the resin was washed with DMF (5×) and DCM (10×). The product was cleaved from the resin by treatment with 2,2,2-trifluoroethanol for 16 hours. The resin was filtered off and washed with DCM (4×). Volatiles were evaporated under reduced pressure, and the residue was triturated with Et_2O (3×) to obtain peptides of sufficient purity.

Synthesis of peptide linkers with toxin moieties

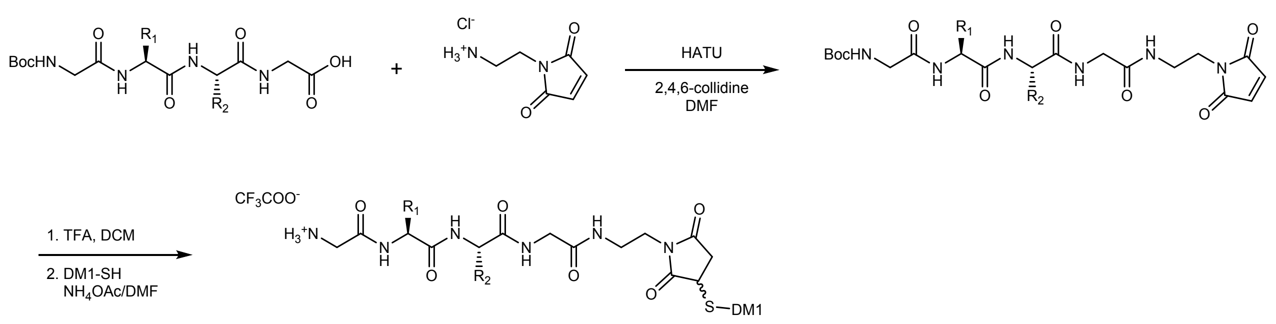

Scheme 1: Synthesis of peptide linkers with toxin moieties

Boc-Gly-Phe-Leu-Gly-OH

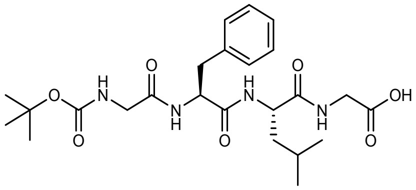

Boc-Gly-Phe-Leu-Gly-OH was prepared according to the general procedure for solid-phase peptide synthesis. (Yield 88%)

^{1}H NMR (400 MHz, CD_3OD) δ 7.32 – 7.13 (m, 5H), 4.70 – 4.60 (m, 1H), 4.48 – 4.38 (m, 1H), 3.89 (d, J = 17.7 Hz, 1H), 3.83 (d, J = 17.7 Hz, 1H), 3.70 (d, J = 16.9 Hz, 1H), 3.63 (d, J = 16.9 Hz, 1H), 3.15 (dd, J = 13.9, 5.7 Hz, 1H), 2.97 (dd, J = 13.9, 8.2 Hz, 1H), 1.71 – 1.54 (m, 3H), 1.43 (s, 9H), 0.94 (d, J = 6.0 Hz, 3H), 0.90 (d, J = 6.0 Hz, 3H).

^{13}C NMR (100 MHz, CD_3OD) δ: 174.7, 173.3, 172.7, 172.6, 138.1, 130.4, 129.6, 127.8, 80.8, 56.0, 53.0, 44.6, 41.9, 41.7, 38.6, 28.7, 25.7, 23.5, 21.9.

LRMS (ESI): m/z calculated for $C_{24}H_{36}N_4O_7Na$ $[M+Na]^+$ 515.2, found: 515.2.
HRMS (ESI): m/z calculated for $C_{24}H_{36}N_4O_7Na$ $[M+Na]^+$ 515.24762, found: 515.24730.

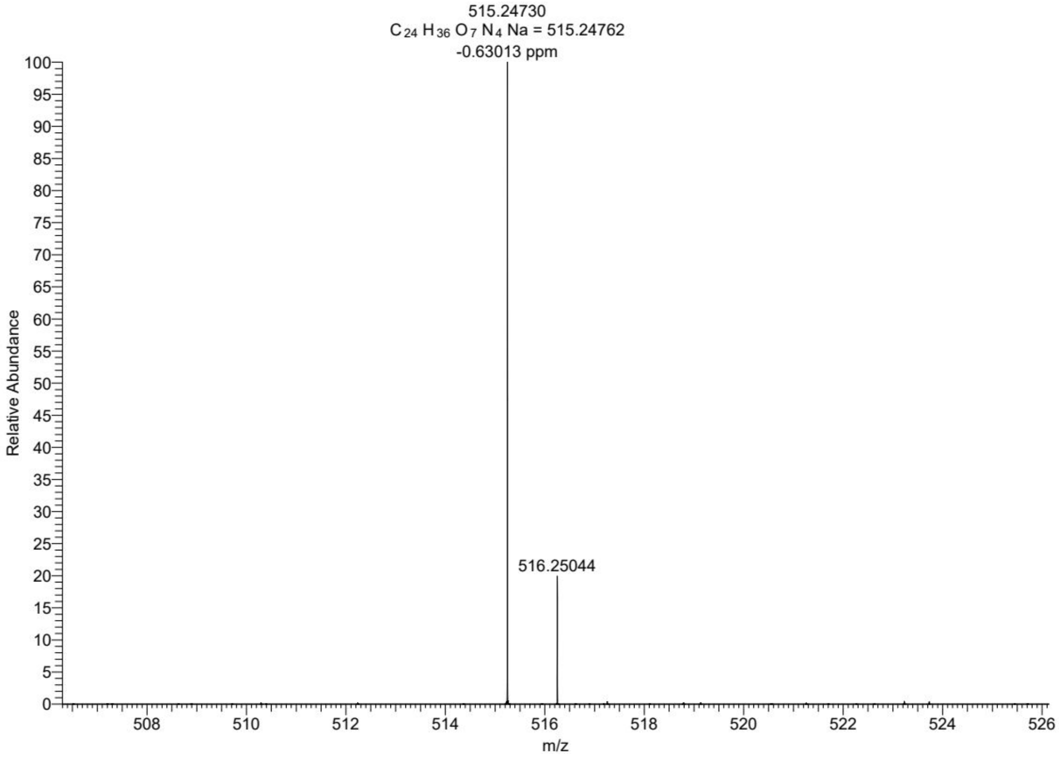

Boc-Gly-Phe-Leu-Gly-maleimide

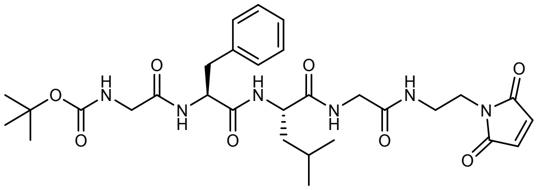

Boc-Gly-Phe-Leu-Gly-OH (50 mg, 100 µmol) and HATU (34 mg, 90 µmol, 0.9 eq.) were dissolved in anhydrous DMF (3 mL). After adding 2,4,6-collidine (40 µL, 300 µmol, 3.0 eq.), the reaction mixture was stirred for 15 minutes at RT. Next, a solution of 1-(2-aminoethyl)-1*H*-pyrrole-2,5-dione hydrochloride (15 mg, 85 µmol, 0.85 eq.) in anhydrous DMF (1 mL) was added via a syringe pump over 1 hour. After 1 hour, the solvent was evaporated, and the residue was dissolved in EtOAc (15 mL). The organic phase was washed with 10% $KHSO_4$ (2 × 15 mL), water (2 × 15 mL), sat. $NaHCO_3$ (2 × 15 mL) and brine (15 mL). The organic phase was then dried with anhydrous $MgSO_4$ and evaporated to obtain 50 mg (94%) of the product as a colorless solid.

^{1}H NMR (400 MHz, $CDCl_3$) δ 7.65 – 7.47 (m, 2H), 7.36 – 7.08 (m, 7H), 6.69 (s, 2H), 5.69 (t, *J* = 5.3 Hz, 1H), 4.95 – 4.84 (m, 1H), 4.67 – 4.57 (m, 1H), 3.94 (dd, *J* = 16.7, 5.9 Hz, 1H), 3.85 (dd, *J* = 16.7, 5.9 Hz, 1H), 3.82 – 3.76 (m, 2H), 3.74 – 3.59 (m, 2H), 3.50 – 3.36 (m, 2H), 3.11 – 3.01 (m, 2H), 1.76 – 1.65 (m, 1H), 1.58 – 1.46 (m, 2H), 1.39 (s, 9H), 0.90 (d, *J* = 6.1 Hz, 7H).

^{13}C NMR (100 MHz, $CDCl_3$) δ 172.5, 171.3, 171.0, 170.3, 169.8, 156.7, 136.1, 134.3, 129.4, 128.8, 127.2, 80.6, 54.6, 52.2, 44.6, 43.2, 41.1, 38.3, 38.2, 37.4, 28.4, 24.8, 23.1, 22.1.

LRMS (ESI): m/z calculated for $C_{30}H_{42}N_6O_8Na$ $[M+Na]^+$ 637.3, found: 637.4.
HRMS (ESI): m/z calculated for $C_{30}H_{42}N_6O_8Na$ $[M+Na]^+$ 637.29563, found: 637.29516.

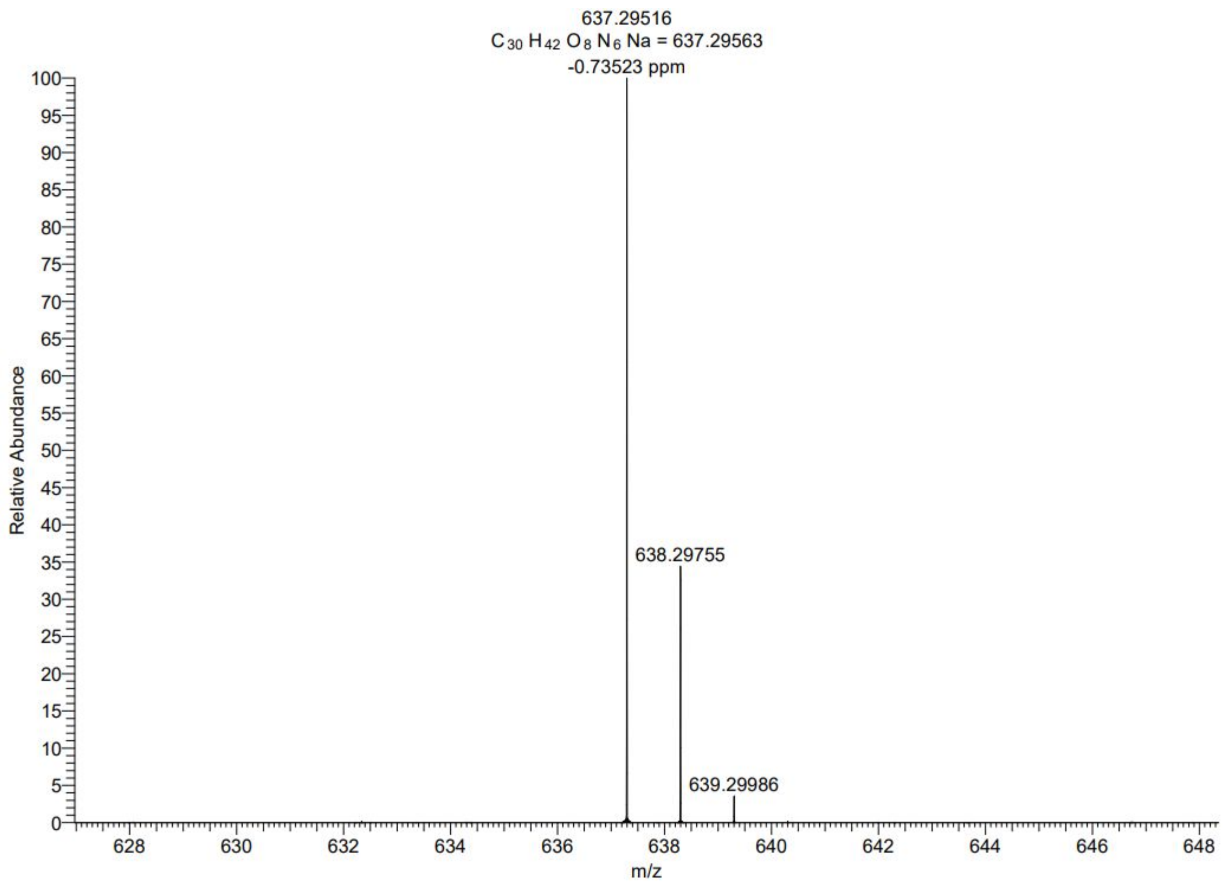

H-Gly-Phe-Leu-Gly-DM1

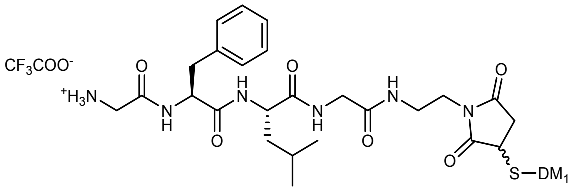

Boc-Gly-Phe-Leu-Gly-maleimide (9 mg, 15 µmol) was dissolved in DCM:TFA (1:1, 1 mL), and the reaction mixture was stirred at RT for 30 min. Then, volatiles were removed, and the residue was redissolved in a mixture of 0.1M aq. NH_4OAc (1 mL) and DMF (0.5 mL). By the addition of aq. $NaHCO_3$ solution, the pH was adjusted to 7.5. Next, a solution of DM1 (11 mg, 18 µmol, 1.0 eq.) in DMF (1 mL) was added. The resulting mixture was stirred at RT for 1 hour. Finally, the product was purified by RP HPLC (gradient 20-60% MeCN in H_2O+0.1% TFA) to obtain 6 mg (30%) of the product as a colorless lyophilizate.

LRMS (MALDI): m/z calculated for $C_{60}H_{82}ClN_9O_{16}SNa$ $[M+Na]^+$ 1274.5, found: 1274.5.
HRMS (MALDI): m/z calculated for $C_{60}H_{82}ClN_9O_{16}SNa$ $[M+Na]^+$ 1274.5181, found: 1274.5164.

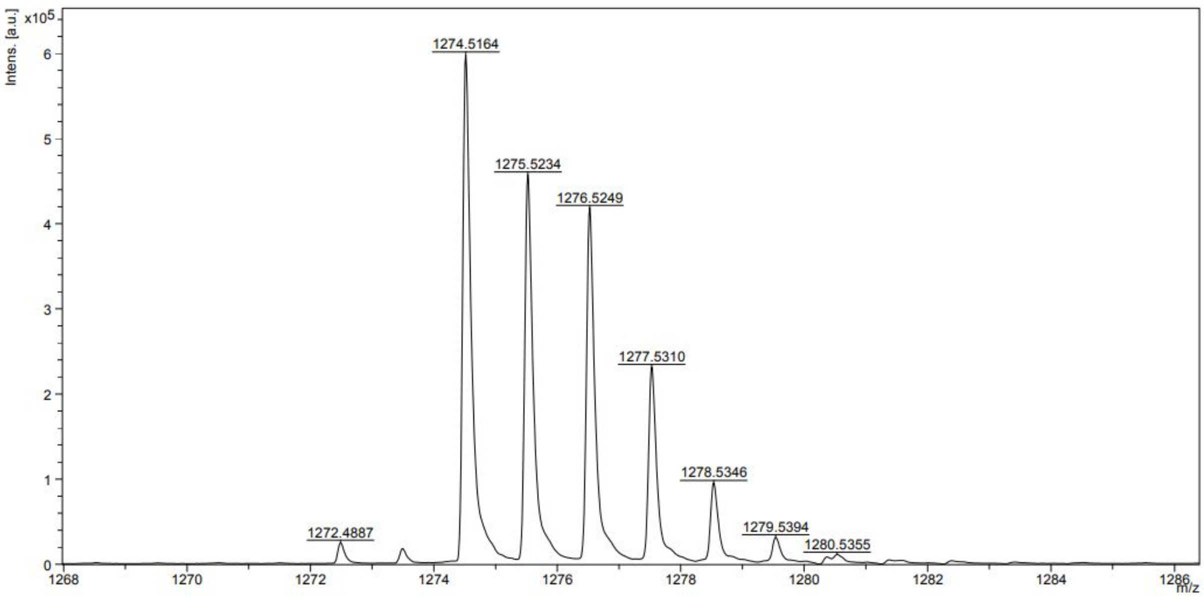

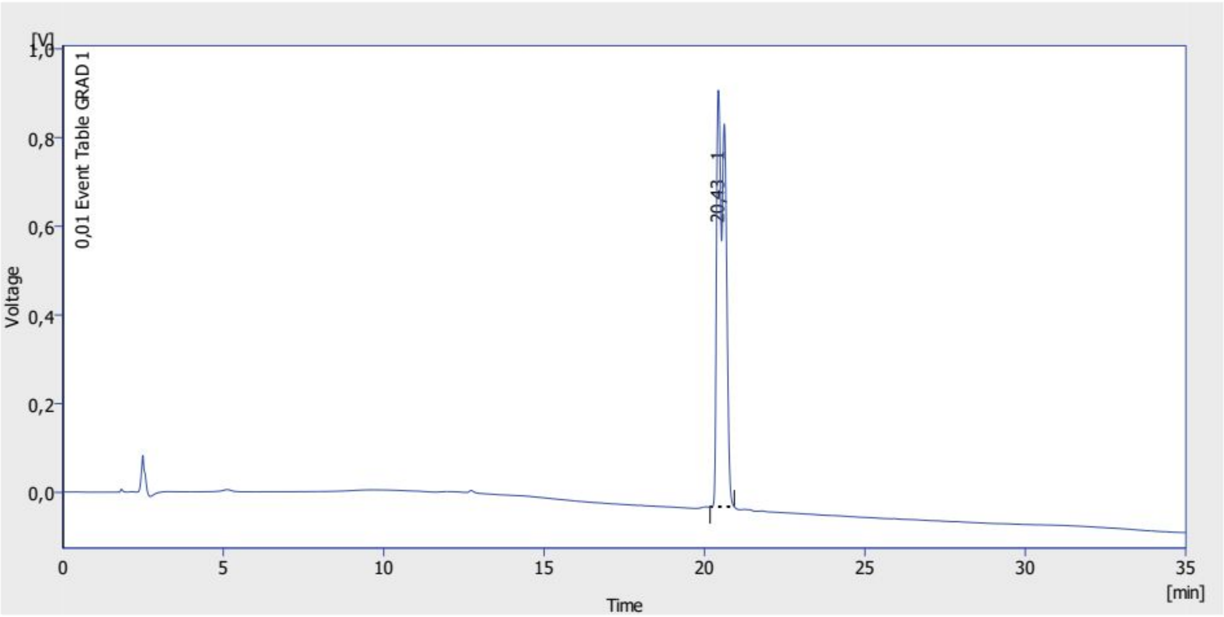

	Reten. Time [min]	Area [mV.s]	Height [mV]	Area [%]	Height [%]	W05 [min]	Compound Name
1	20,432	17352,938	939,284	100,0	100,0	0,34	
	Total	17352,938	939,284	100,0	100,0		

Boc-Gly-Val-Cit-Gly-OH

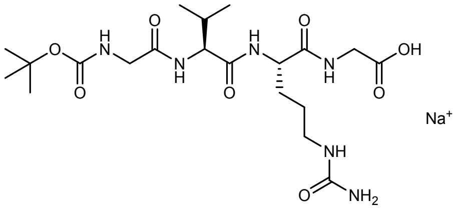

Boc-Gly-Val-Cit-Gly-OH was prepared according to the general procedure for solid-phase peptide synthesis. (Yield 82%)

^{1}H NMR (400 MHz, CD_3OD) δ 4.48 – 4.38 (m, 1H), 4.28 – 4.19 (m, 1H), 4.02 – 3.80 (m, 2H), 3.75 (s, 2H), 3.20 – 3.05 (m, 2H), 2.16 – 2.02 (m, 1H), 1.94 – 1.81 (m, 1H), 1.76 – 1.63 (m, 1H), 1.62 – 1.51 (m, 2H), 1.45 (s, 9H), 0.97 (d, *J* = 6.9 Hz, 3H), 0.94 (d, *J* = 6.9 Hz, 3H).

^{13}C NMR (100 MHz, CD_3OD) δ 174.3, 173.5, 172.8 (2C), 162.3, 158.5, 80.8, 60.1, 54.1, 44.6, 41.8, 40.3, 31.9, 30.3, 28.7, 27.5, 19.8, 18.5.

LRMS (ESI): m/z calculated for $C_{20}H_{36}N_6O_8Na$ $[M+Na]^+$ 511.2, found: 511.3.
HRMS (ESI): m/z calculated for $C_{20}H_{36}N_6O_8Na$ $[M+Na]^+$ 511.24868, found: 511.24845.

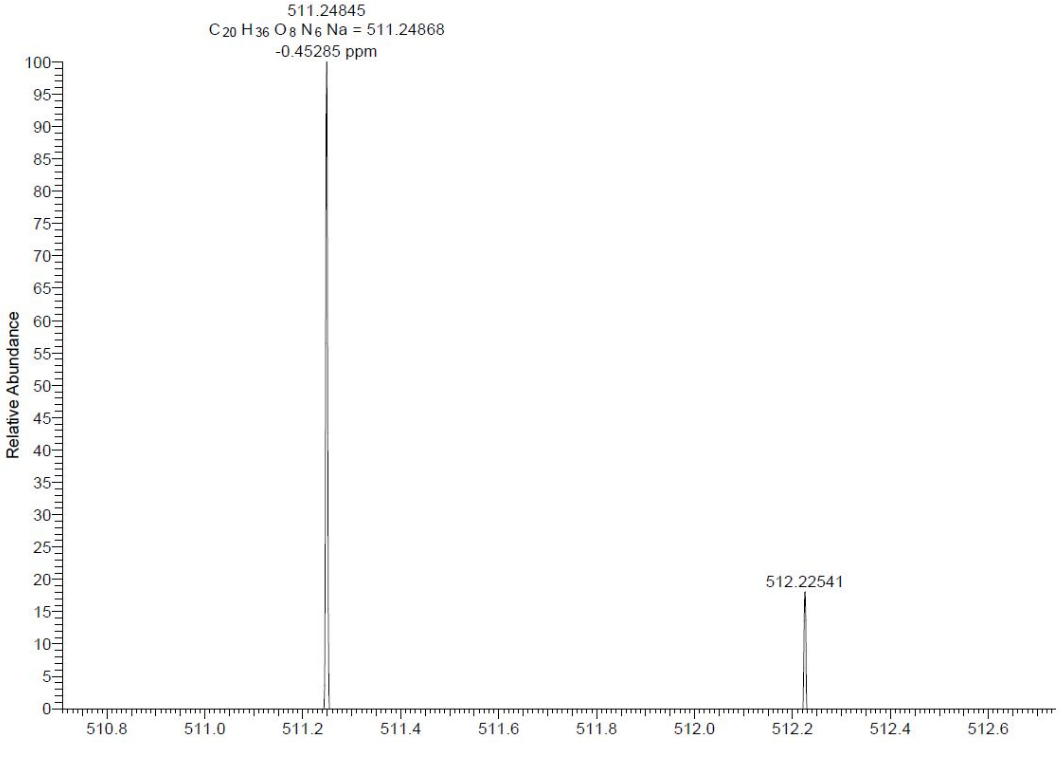

Boc-Gly-Val-Cit-Gly-maleimide

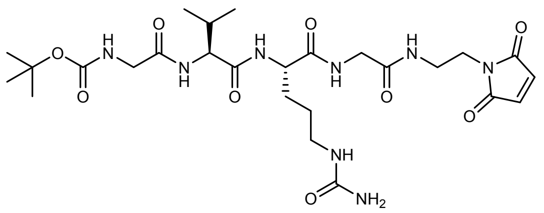

Boc-Gly-Val-Cit-Gly-OH (40 mg, 82 µmol) and HATU (28 mg, 74 µmol, 0.9 eq.) were dissolved in anhydrous DMF (3 mL). After adding 2,4,6-collidine (33 µL, 246 µmol, 3.0 eq.), the reaction mixture was stirred at room temperature for 15 minutes. Next, a solution of 1-(2-aminoethyl)-1*H*-pyrrole-2,5-dione hydrochloride (12 mg, 70 µmol, 0.85 eq.) in anhydrous DMF (1 mL) was added via a syringe pump over 1 hour. After 1 hour, the solvent was evaporated, and the residue was purified by RP HPLC (gradient 5-70% MeCN in H_2O+0.1% TFA) to obtain 45 mg (90%) of the product as a colorless lyophilizate.

^{1}H NMR (400 MHz, CD_3OD) δ 6.82 (s, 2H), 4.27 – 4.19 (m, 2H), 3.90 – 3.56 (m, 6H), 3.52 – 3.41 (m, 1H), 3.37 – 3.25 (m, 1H, overlaps with CD_3OD), 3.21 – 3.04 (m, 2H), 2.17 – 2.04 (m, 1H), 1.91 – 1.78 (m, 1H), 1.77 – 1.64 (m, 1H), 1.64 – 1.46 (m, 2H), 1.44 (s, 9H), 0.96 (d, J = 6.8 Hz, 3H), 0.94 (d, J = 6.8 Hz, 3H).

^{13}C NMR (100 MHz, CD_3OD) δ 174.5, 174.0, 172.9, 172.6, 171.8, 162.2, 158.5, 135.5, 80.8, 60.0, 55.2, 44.7, 43.5, 40.3, 38.7, 38.0, 32.0, 29.4, 28.7, 27.6, 19.7, 18.4.

LRMS (ESI): m/z calculated for $C_{26}H_{42}N_8O_9Na$ $[M+Na]^+$ 633.3, found: 633.3.
HRMS (ESI): m/z calculated for $C_{26}H_{42}N_8O_9Na$ $[M+Na]^+$ 633.29670, found: 633.29642.

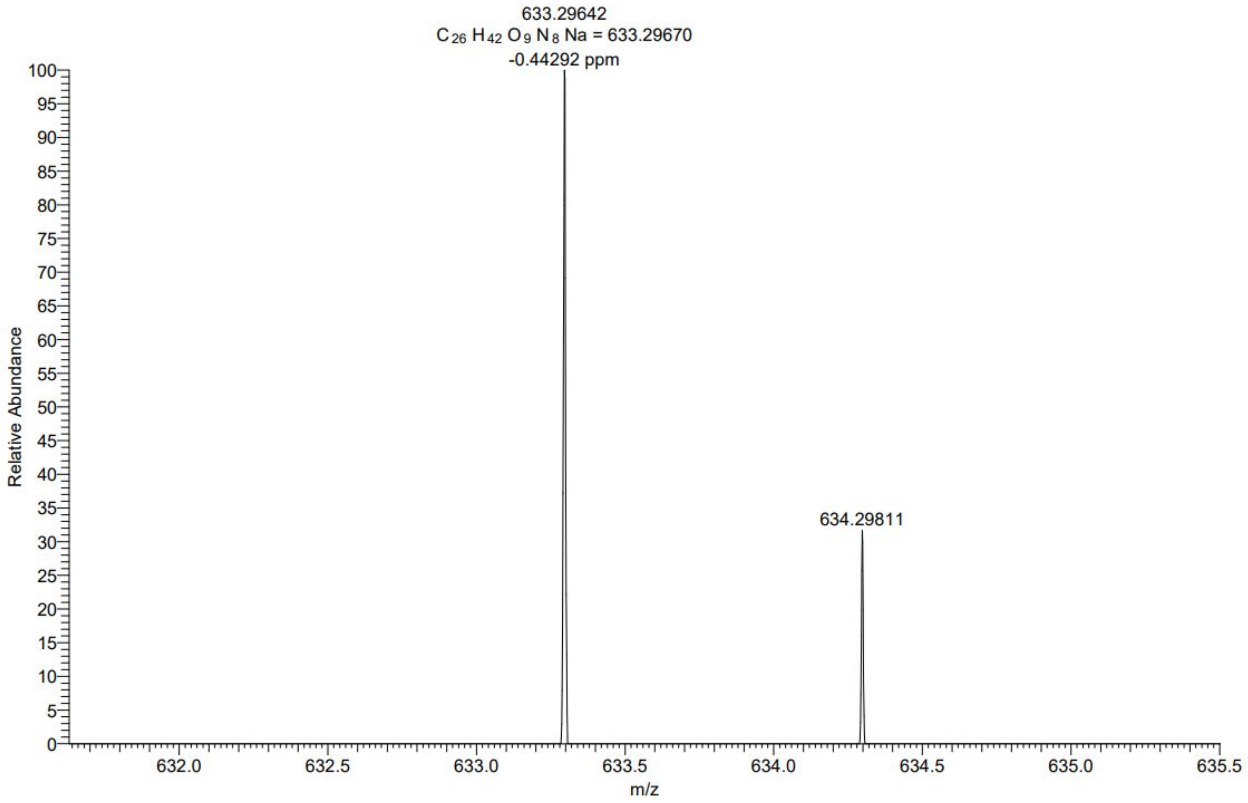

H-Gly-Val-Cit-Gly-DM1

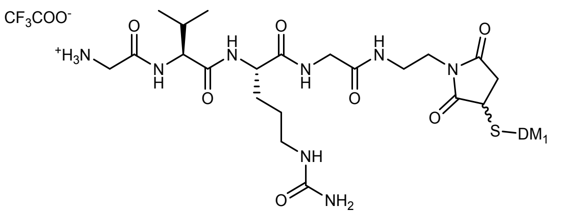

Boc-Gly-Val-Cit-Gly-maleimide (11 mg, 18 μmol) was dissolved in DCM:TFA (1:1, 1 mL), and the reaction mixture stirred at RT for 30 min. Then, volatiles were removed, and the residue was redissolved in a mixture of 0.1M aq. NH_4OAc (1 mL) and DMF (0.5 mL). By the addition of aq. $NaHCO_3$ solution, the pH was adjusted to 7.5. Next, a solution of DM1 (13 mg, 18 μmol, 1.0 eq.) in DMF (1 mL) was added. The resulting mixture was stirred at RT for 1 hour. Finally, the product was purified by RP HPLC (gradient 20-60% MeCN in H_2O+0.1% TFA) to obtain 10 mg (42%) of the product as a colorless lyophilizate.

LRMS (ESI): m/z calculated for $C_{56}H_{82}ClN_{11}O_{17}SNa$ $[M+Na]^+$ 1270.5, found: 1270.5.
HRMS (ESI): m/z calculated for $C_{56}H_{82}ClN_{11}O_{17}SNa$ $[M+Na]^+$ 1270.5192, found: 1270.5172.

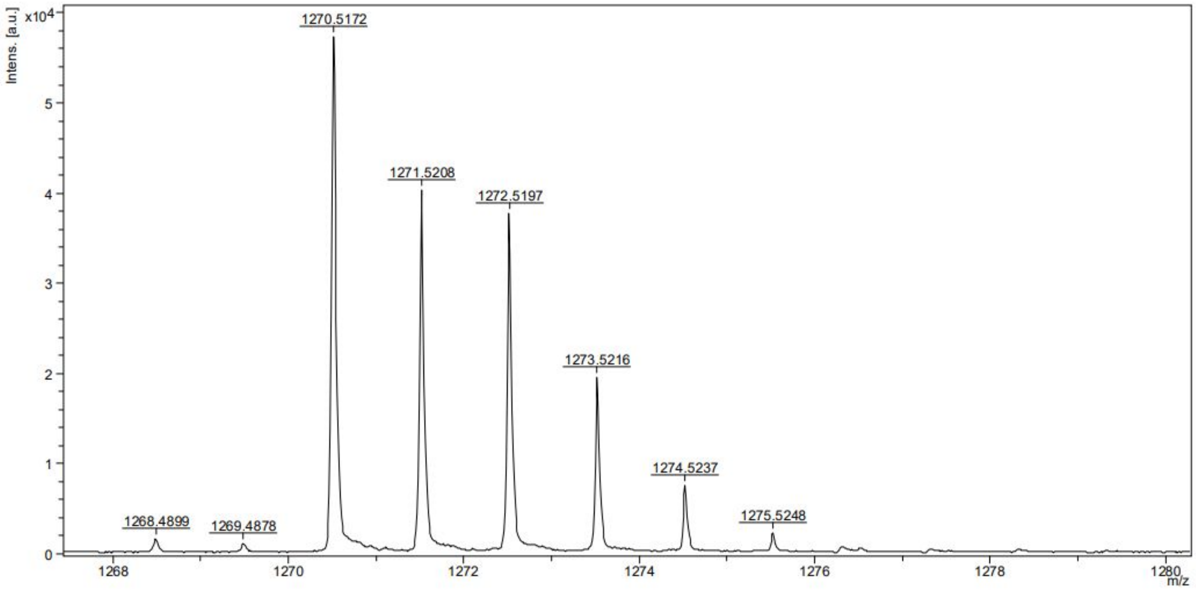

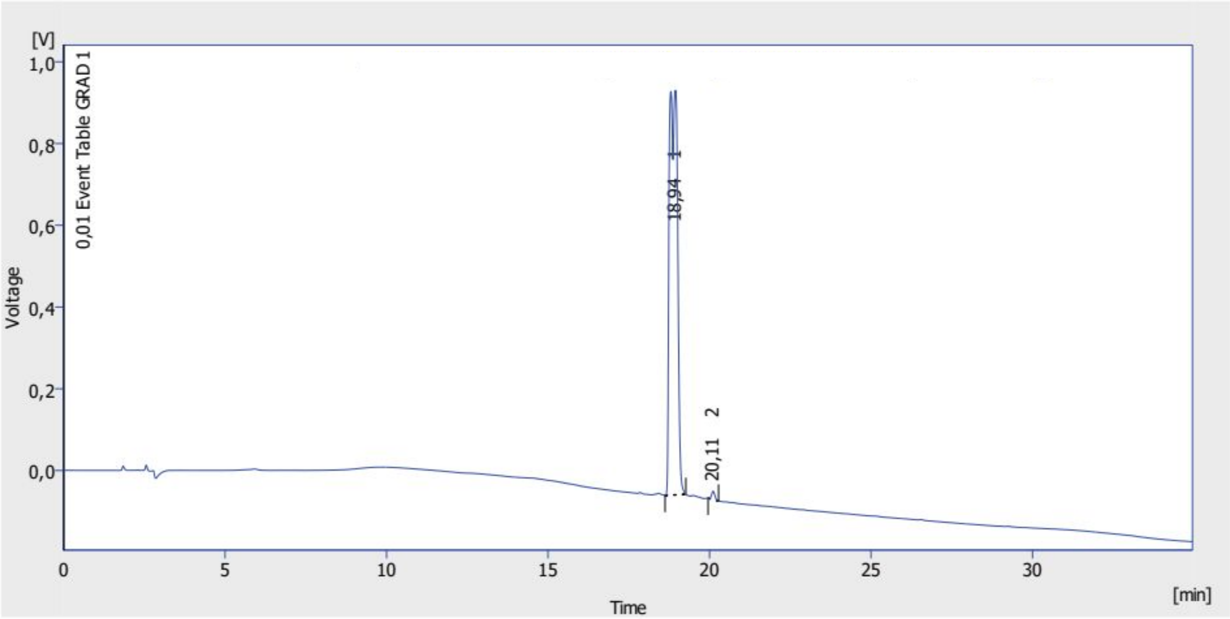

	Reten. Time [min]	Area [mV.s]	Height [mV]	Area [%]	Height [%]	W05 [min]	Compound Name
1	18,944	17624,495	989,966	99,2	98,0	0,30	
2	20,112	144,127	20,566	0,8	2,0	0,11	
	Total	17768,622	1010,532	100,0	100,0		

III. Synthesis of CD64-targeted polymer-drug conjugates

The general procedure for the synthesis of TPDCs is described in chapter Materials and Methods, in section 2.3 Synthesis of TPDCs and 2.4. Characterization of polymer precursors and TPDCs.

Synthesis of fC1

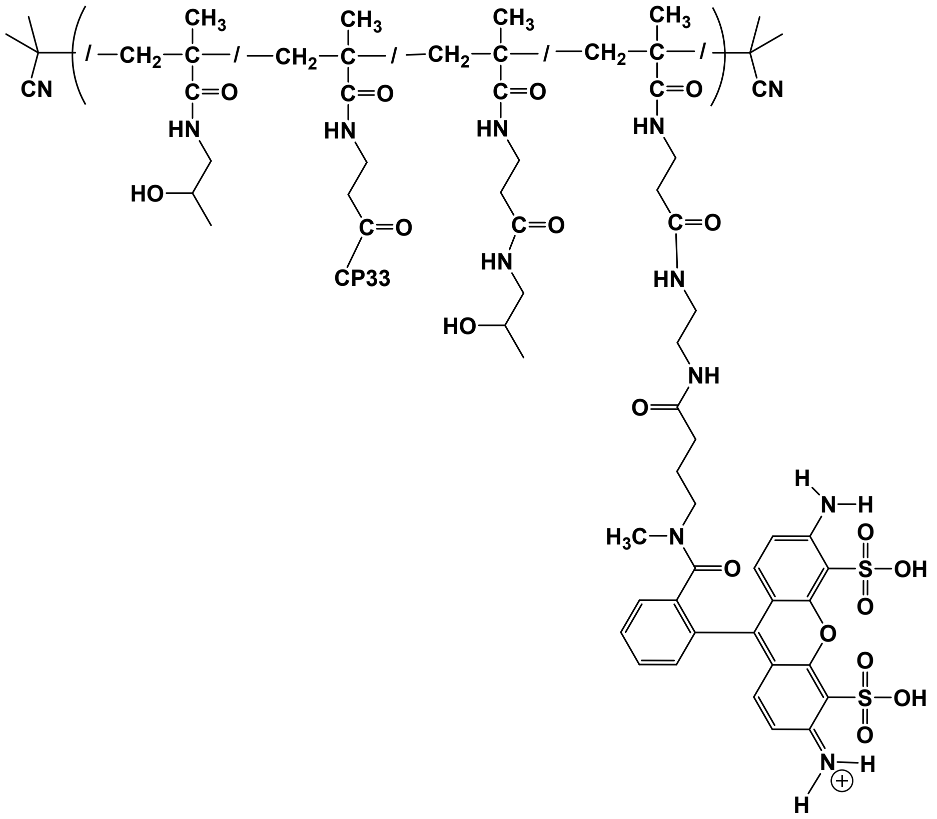

The copolymer precursor P2 (13 mg, 8.7×10^{-3} mmol TT groups), cp33 peptide (3.71 mg, 1.93×10^{-3} mmol) and ATTO488 (0.46 mg, 5.35×10^{-4} mmol) were dissolved in DMSO (300 µL), and then DIPEA (1.7 µL, 9.85×10^{-3} mmol) was added. The reaction was carried out at RT for 4 hours. Residual TT reactive groups were removed by the addition of 2 µL of 1-amino-propan-2-ol (2.59 $\times 10^{-2}$ mmol), and the reaction was stirred for 10 minutes. The TPDC-containing reaction mixture was diluted with 1 mL of methanol, and the TPDC was separated on a 1.5 x 18 cm chromatography column with Sephadex LH-20 in methanol, equipped with the UV-Vis detector Azura UVD 2.1S (Knauer). Methanol was evaporated, the TPDC was dissolved in 1,5 mL of Milli-Q water and purified on a PD10 column and lyophilized. The yield of the **fC1** was 13 mg. The content of anti-CD64 peptide was 12.2 wt%; ATTO488 2.5 wt% and M_w = 121 000 g mol^{-1}and $Đ$ = 1.56.

Synthesis of fC2

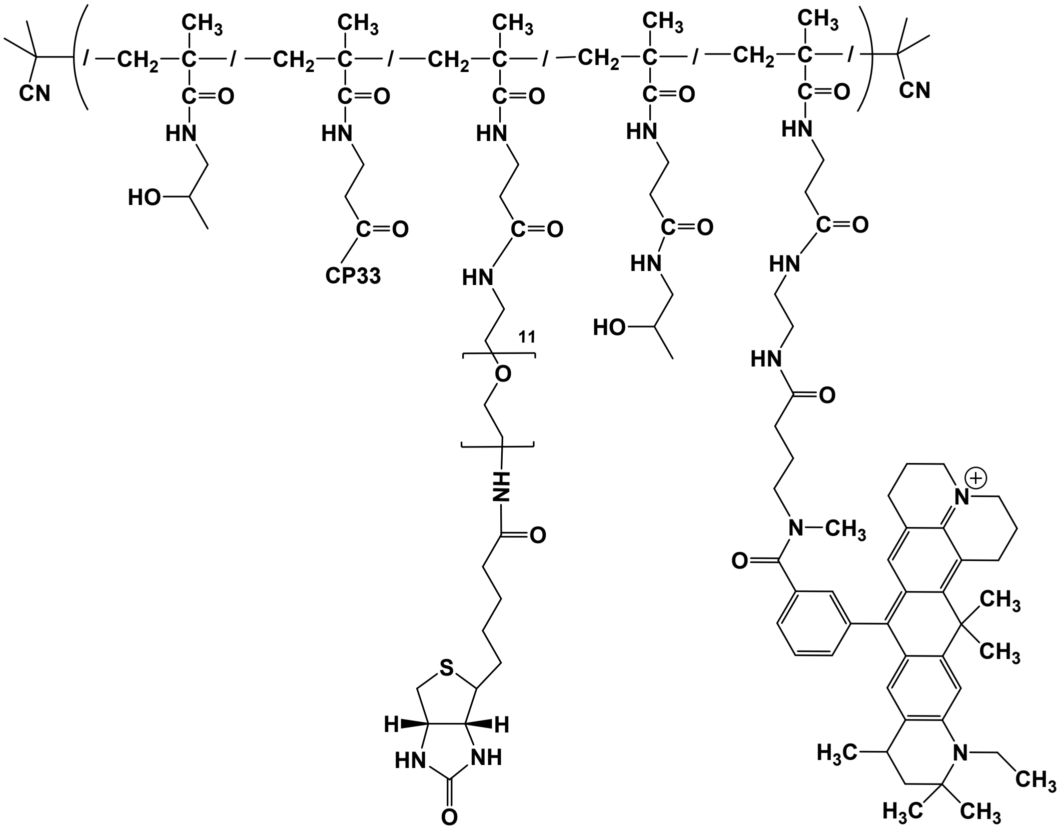

The copolymer precursor P1 (7.1 mg, 5.97×10^{-3} mmol TT groups), cp33 peptide (3.24 mg, 1.68×10^{-3} mmol), ATTO647N (0.40 mg, 4.37×10^{-4} mmol) and NH2-PEG$_{11}$-biotin (2.0 mg, 2.59×10^{-3} mmol) were dissolved in DMSO (300 µL), and then DIPEA (3.7 µL, 2.14×10^{-2} mmol) was added. The synthetic procedure and purification was performed analogously to the synthesis of fC1, which is describe in details above in corresponding paragraph. The yield of the **fC2** was 11 mg. The content of anti-CD64 peptide was 21.3 wt%; ATTO647N 1.96 wt%; NH_2-PEG$_{11}$-biotin = 13.4 wt%. M_w and $Đ$ were not determined due to the interaction of the excitation maximal wavelength of ATTO647N with the wavelength of the laser in a multiangle light-scattering detector DAWN 8.

Synthesis of fC3

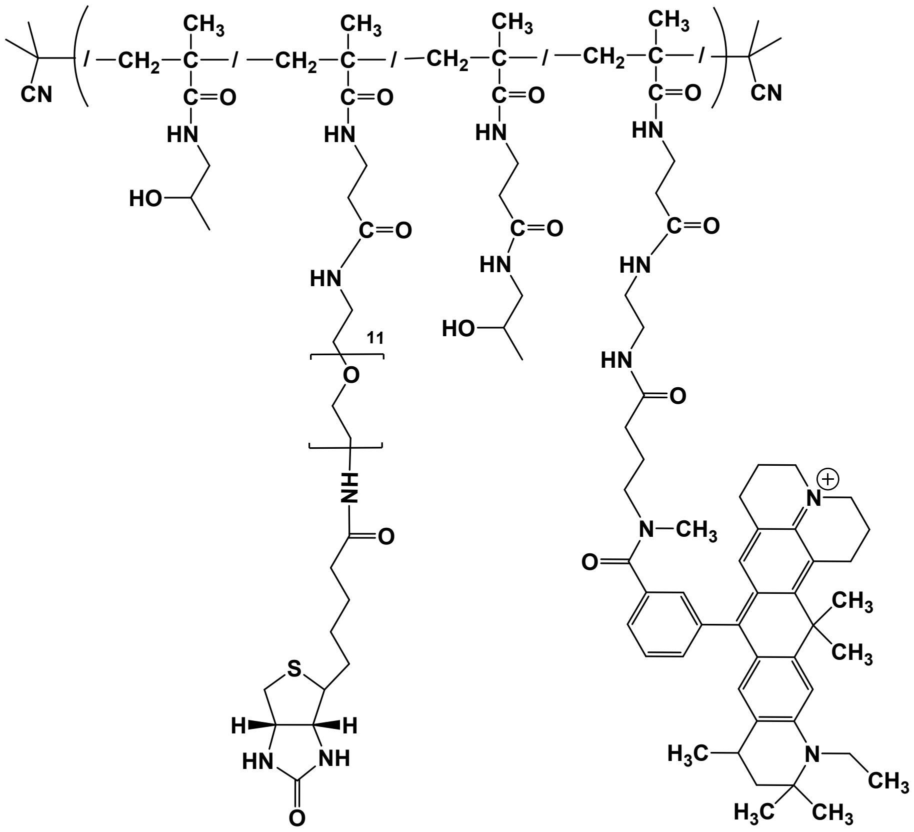

The copolymer precursor P1 (8.1 mg, 6.84 × 10^{-3} mmol TT groups), ATTO647N (0.36 mg, 3.93 × 10^{-4} mmol) and NH_2-PEG_{11}-biotin (2.0 mg, 2.59 × 10^{-3} mmol) were dissolved in DMSO (300 μL), and then DIPEA (4.9 μL, 2.8 × 10^{-2} mmol) was added. The synthetic procedure and purification was performed analogously to the synthesis of fC1, which is describe in details above in corresponding paragraph. The yield of the **fC3** was 9.2 mg. The content of ATTO647N was 1.84 wt%; NH_2-PEG_{11}-biotin 15.3 wt%. M_w and $Đ$ were not determined due to the interaction of the excitation maximal wavelength of ATTO647N with the wavelength of the laser in the multiangle light-scattering detector DAWN 8.

Synthesis of C4

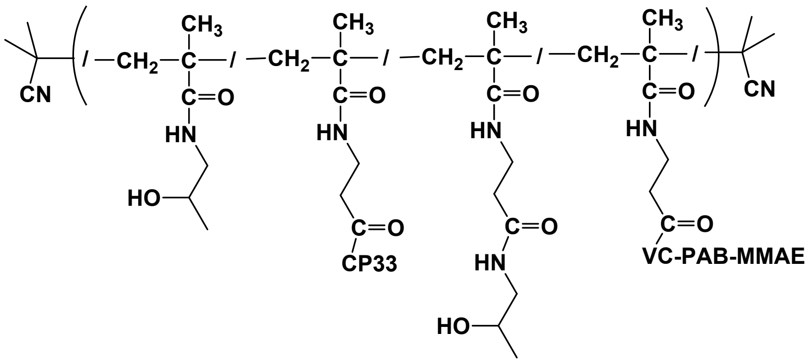

The copolymer precursor P1 (6.95 mg, 5.83 × 10^{-3} mmol TT groups), cp33 peptide (2.62 mg, 1.36 × 10^{-3} mmol) and NH_2-Val-Cit-PAB-MMAE (1.46 mg, 1.3 × 10^{-4} mmol) were dissolved in DMSO (300 µL), and then DIPEA (2.3 µL, 1.33 × 10^{-2} mmol) was added. The synthetic procedure and purification was performed analogously to the synthesis of fC1, which is describe in details above in corresponding paragraph. The yield of the **C4** was 9.8 mg. The content of anti-CD64 peptide was 19.3 wt%; NH_2-Val-Cit-PAB-MMAE 9.2 wt% and M_w = 306 000 g $mol^{-1}$and $Đ$ = 1.05.

Synthesis of C5

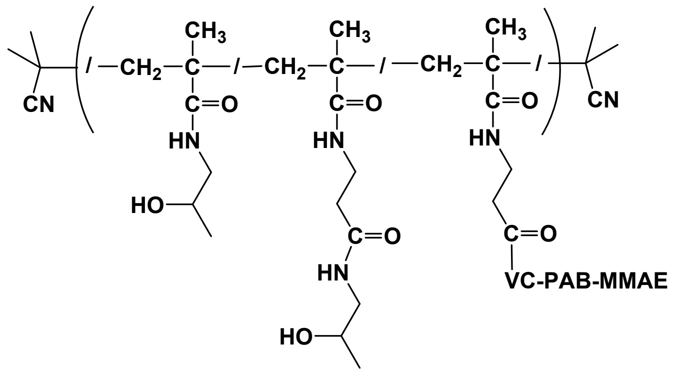

The copolymer precursor P1 (8.1 mg, 6.8 × 10^{-3} mmol TT groups) and NH_2-Val-Cit-PAB-MMAE (1.2 mg, 1.07 × 10^{-3} mmol) were dissolved in DMSO (300 µL), and then DIPEA (0.9 µL, 5.34 × 10^{-3} mmol) was added. The synthetic procedure and purification was performed analogously to the synthesis of fC1, which is describe in details above in corresponding paragraph. The yield of the **C5** was 8.9 mg. The content of NH_2-Val-Cit-PAB-MMAE was 8.5 wt% and M_w = 90 000 g mol^{-1} and $Đ$ = 1.17.

Synthesis of C6

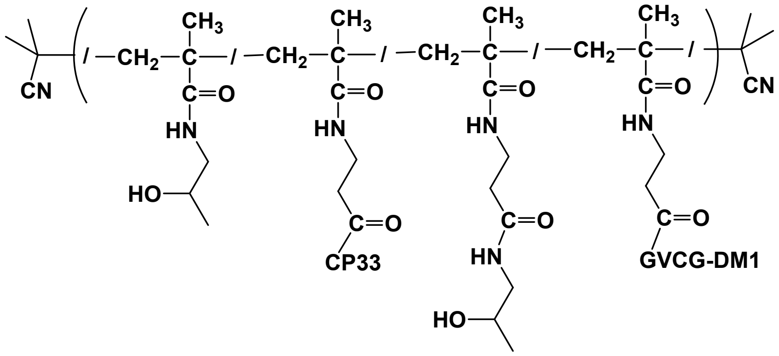

The copolymer precursor P1 (7.2 mg, 6.08 × 10^{-3} mmol TT groups), cp33 peptide (3.43 mg, 1.78 × 10^{-3} mmol) and NH_2-Gly-Val-Cit-Gly-DM1 (1.8 mg, 1.33 × 10^{-3} mmol) were dissolved in DMSO (300 µL), and then DIPEA (2.7 µL, 1.56 × 10^{-2} mmol) was added. The synthetic procedure and purification was performed analogously to the synthesis of fC1, which is describe in details above in corresponding paragraph. The yield of the **C6** was 10.3 mg. The content of anti-CD64 peptide was 24.2 wt%; NH_2-Gly-Val-Cit-Gly-DM1 12.5 wt% and M_w = 160 000 g mol^{-1} and $Đ$ = 1.21.

Synthesis of C7

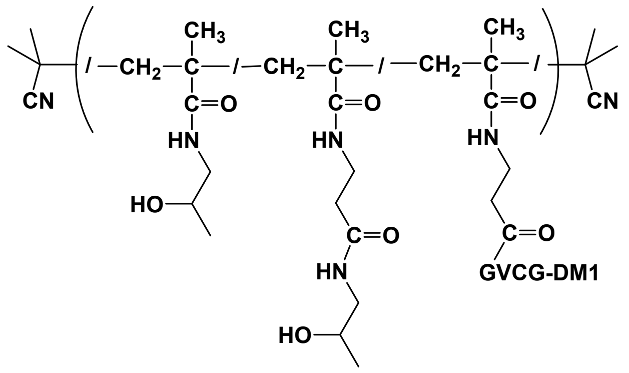

The copolymer precursor P1 (8.0 mg, 6.71×10^{-3} mmol TT groups) and NH_2-Gly-Val-Cit-Gly-DM1 (1.82 mg, 1.34×10^{-3} mmol) were dissolved in DMSO (300 μL), and then DIPEA (1.2 μL, 6.67×10^{-3} mmol) was added. The synthetic procedure and purification was performed analogously to the synthesis of fC1, which is describe in details above in corresponding paragraph. The yield of the **C7** was 8.2 mg. The content of NH_2-Gly-Val-Cit-Gly-DM1 was 16.6 wt% and M_w = 89 000 g mol^{-1} and $Đ$ = 1.22.

Synthesis of C8

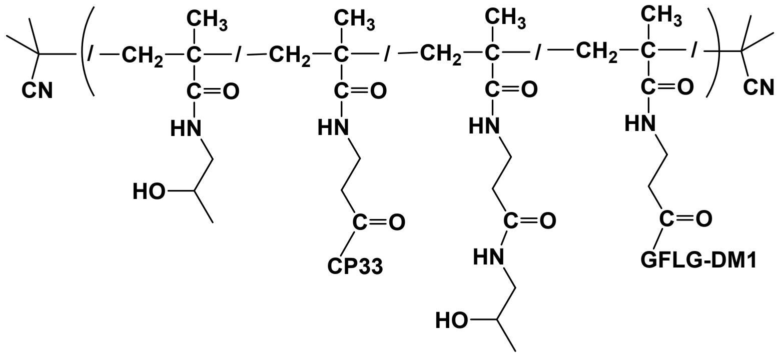

The copolymer precursor P1 (6.27 mg, 5.26×10^{-3} mmol TT groups), cp33 peptide (3.13 mg, 1.62×10^{-3} mmol) and NH_2-Gly-Phe-Leu-Gly-DM1 (1.65 mg, 1.21×10^{-3} mmol) were dissolved in DMSO (300 μL), and then DIPEA (2.47 μL, 1.42×10^{-2} mmol) was added. The synthetic procedure and purification was performed analogously to the synthesis of fC1, which is describe in details above in corresponding paragraph. The yield of the **C8** was 9.3 mg. The content of anti-CD64 peptide was 26.1 wt%; NH_2-Gly-Phe-Leu-Gly-DM1 14.5 wt% and M_w = 120 000 g mol^{-1} and $Đ$ = 1.15.

Synthesis of C9

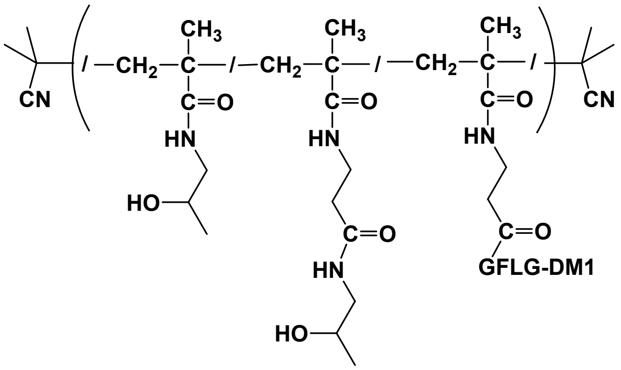

The copolymer precursor P1 (8.3 mg, 6.99 × 10^{-3} mmol TT groups) and NH_2-Gly-Phe-Leu-Gly-DM1 (1.75 mg, 1.28 × 10^{-3} mmol) were dissolved in DMSO (300 μL), and then DIPEA (1.1 μL, 6.4 × 10^{-3} mmol) was added. The synthetic procedure and purification was performed analogously to the synthesis of fC1, which is describe in details above in corresponding paragraph. The yield of the **C9** was 9.9 mg. The content of NH_2-Gly-Phe-Leu-Gly-DM1 was 15.7 wt% and M_w = 76 000 g mol^{-1} and $Đ$ = 1.13.

Synthesis of C10

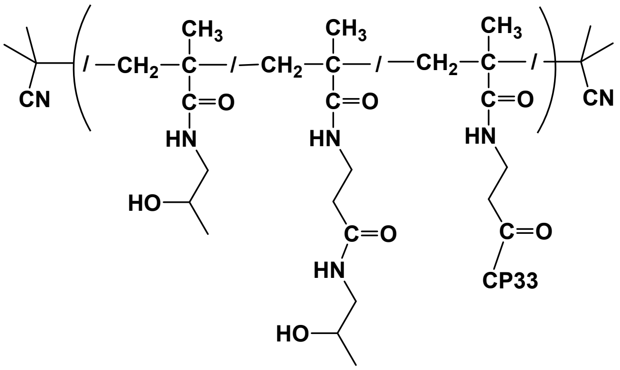

The copolymer precursor P3 (8.07 mg, 5.65 × 10^{-3} mmol TT groups) and cp33 peptide (1.61 mg, 0.836 × 10^{-3} mmol) were dissolved in DMSO (300 μL), and then DIPEA (0.6 μL, 3.34 × 10^{-3} mmol) was added. The synthetic procedure and purification was performed analogously to the synthesis of fC1, which is describe in details above in corresponding paragraph. The yield of the **C10** was 9.0 mg. The content of cp33 peptide was 10.6 wt% and M_w = 81 000 g mol^{-1} and $Đ$ = 1.18.

Synthesis of C11

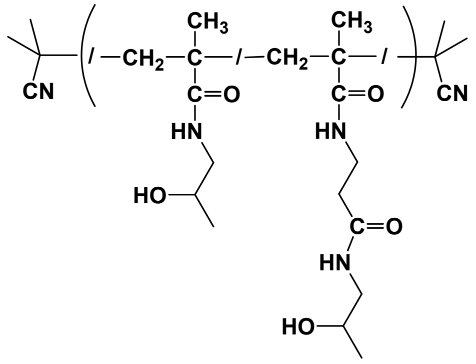

The copolymer precursor P4 (15.3 mg, 9.85×10^{-3} mmol TT groups) was dissolved in DMSO (300 µL) and thiazolidine-2-thione reactive groups were removed by the addition of 2 µl of 1-amino-propan-2-ol (2.59×10^{-2} mmol), and the reaction was stirred for 10 minutes. The **C11** containing reaction mixture was diluted with 1 ml of methanol, and the **C11** was separated on a 1.5 x 18 cm chromatography column with Sephadex LH-20 in methanol, equipped with the UV-Vis detector Azura UVD 2.1S (Knauer). Methanol was evaporated, the **C11** was dissolved in Milli-Q water and purified on a PD10 column and lyophilized. The M_w = 65 000 g mol^{-1} and $Đ$ = 1.05.

Synthesis of C12

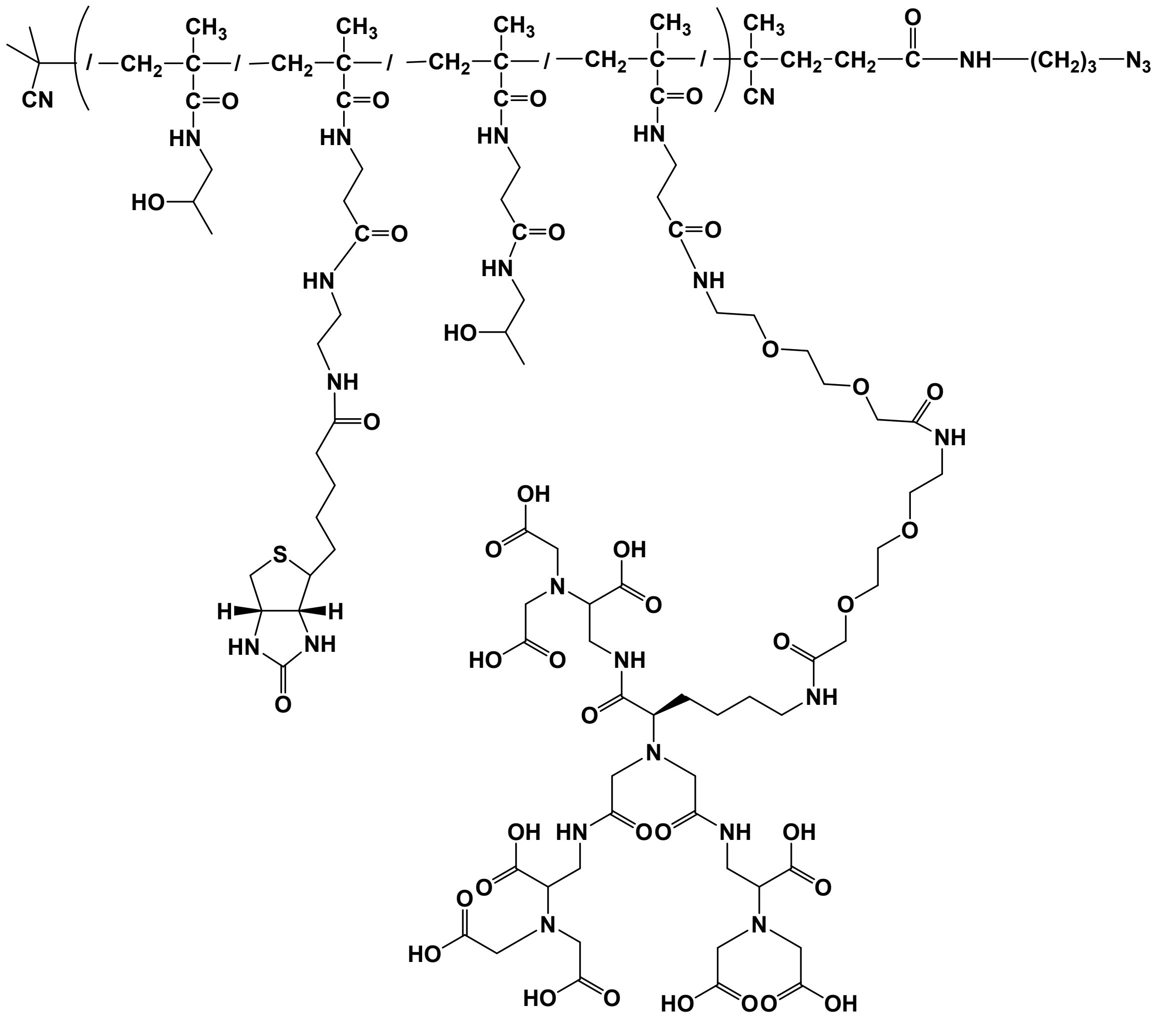

The copolymer precursor P1 (14.9 mg, 1.25 × 10^{-2} mmol TT groups), NH_2-OcOcTrisNTA (4.43 mg, 3.48 × 10^{-3} mmol) and NH_2-ED-biotin (1.5 mg, 3.75 × 10^{-4} mmol) were dissolved in DMSO (300 µL), and then DIPEA (9.3 µL, 5.35 × 10^{-2} mmol) was added. The synthetic procedure and purification was performed analogously to the synthesis of fC1, which is describe in details above in corresponding paragraph. The yield of the **C12** was 17.9 mg. The content of NH_2-OcOcTrisNTA was 19.7 wt%; NH_2-ED-biotin 3.2 wt% and M_w = 95 000 g mol^{-1} and $Đ$ = 1.19. The characterization of polymer precursors is summarized in Supplementary Table S1.

Chromatograms:

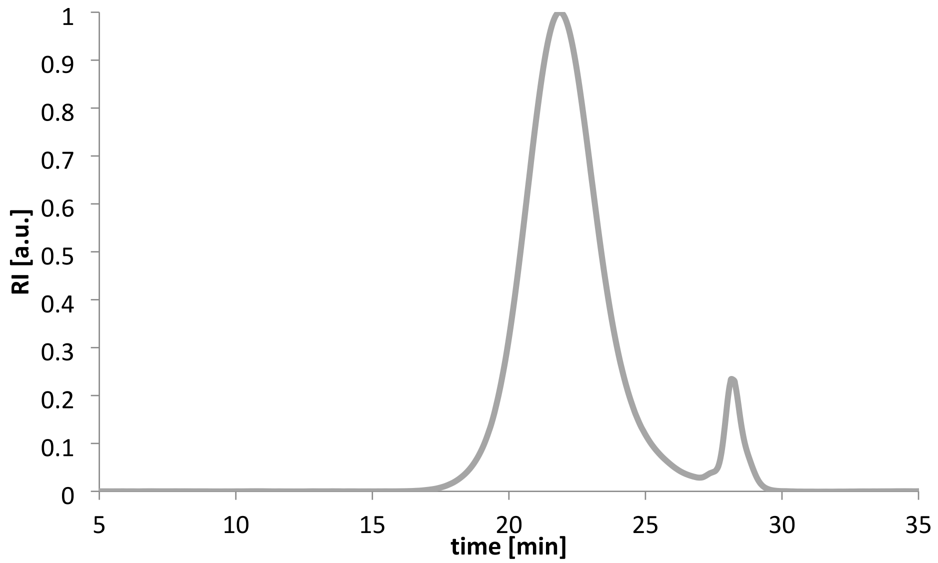

GPC chromatogram of polymer precursor P1, plotting dRI detector data.

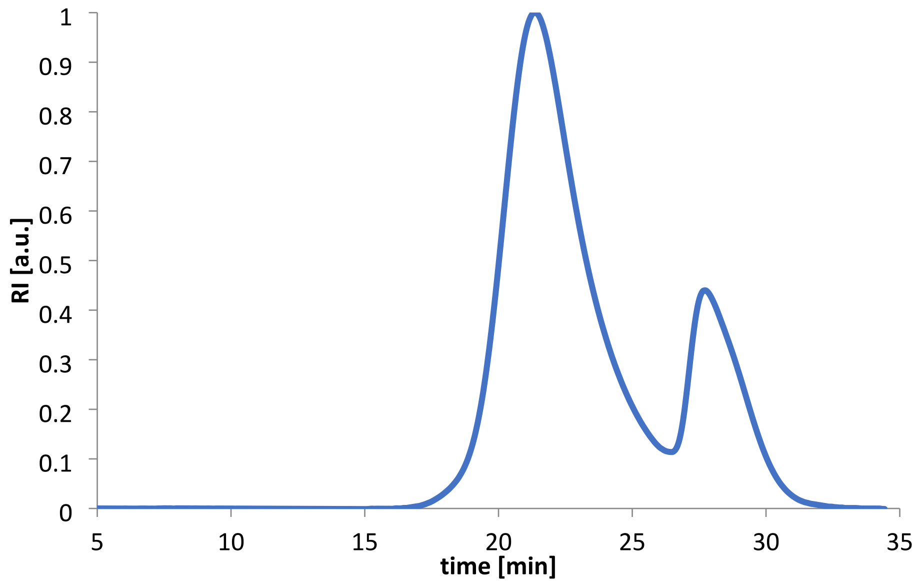

GPC chromatogram of polymer precursor P2, plotting dRI detector data.

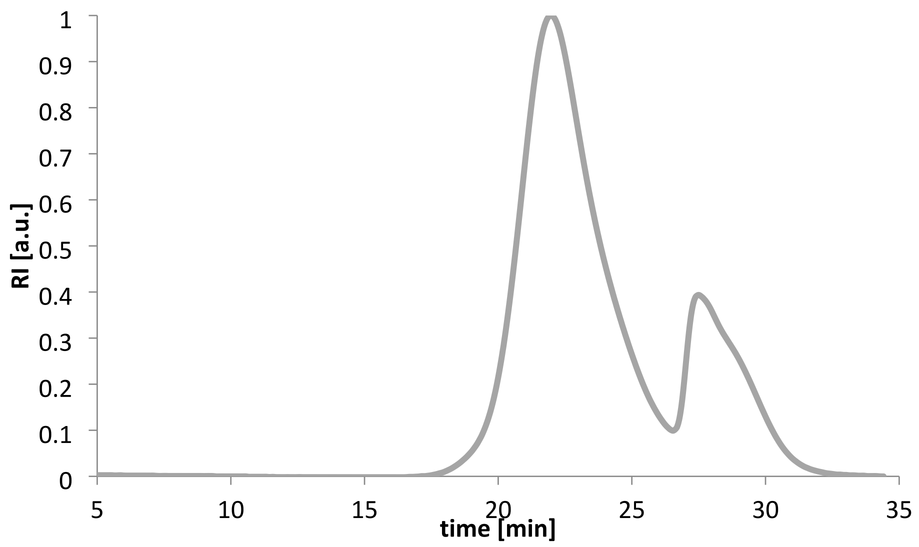

GPC chromatogram of polymer precursor P3, plotting dRI detector data.

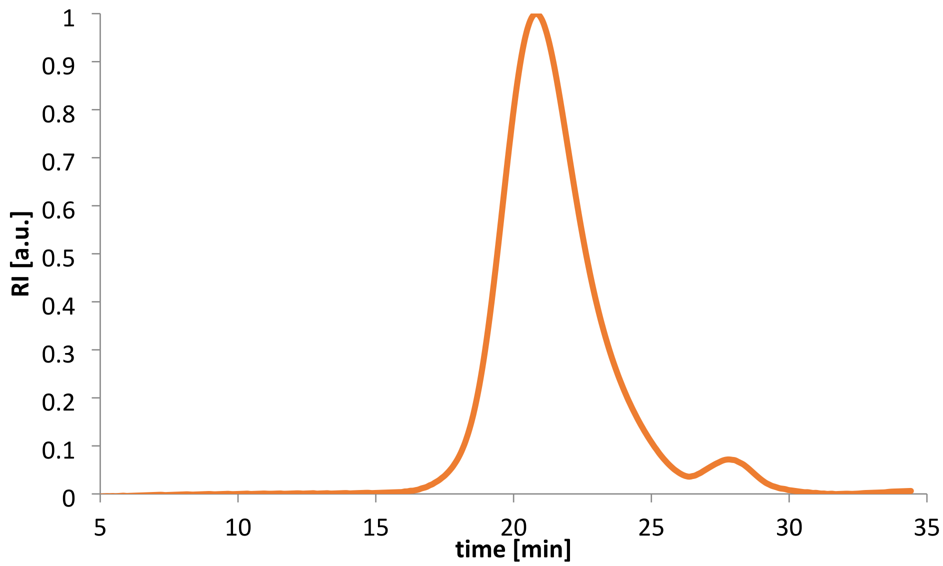

GPC chromatogram of polymer precursor P4, plotting dRI detector data.

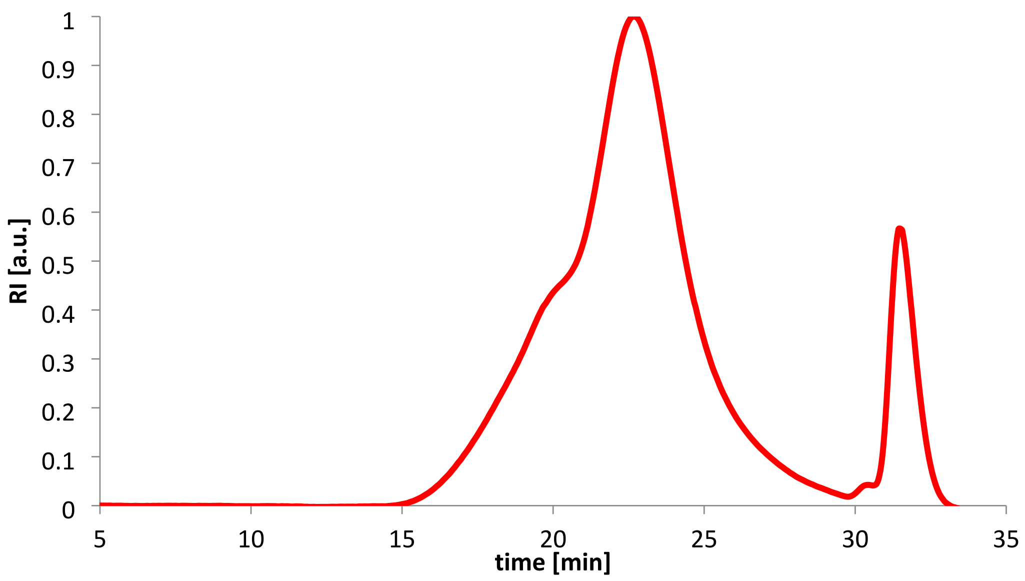

GPC chromatogram of **fC1**, plotting dRI detector data.

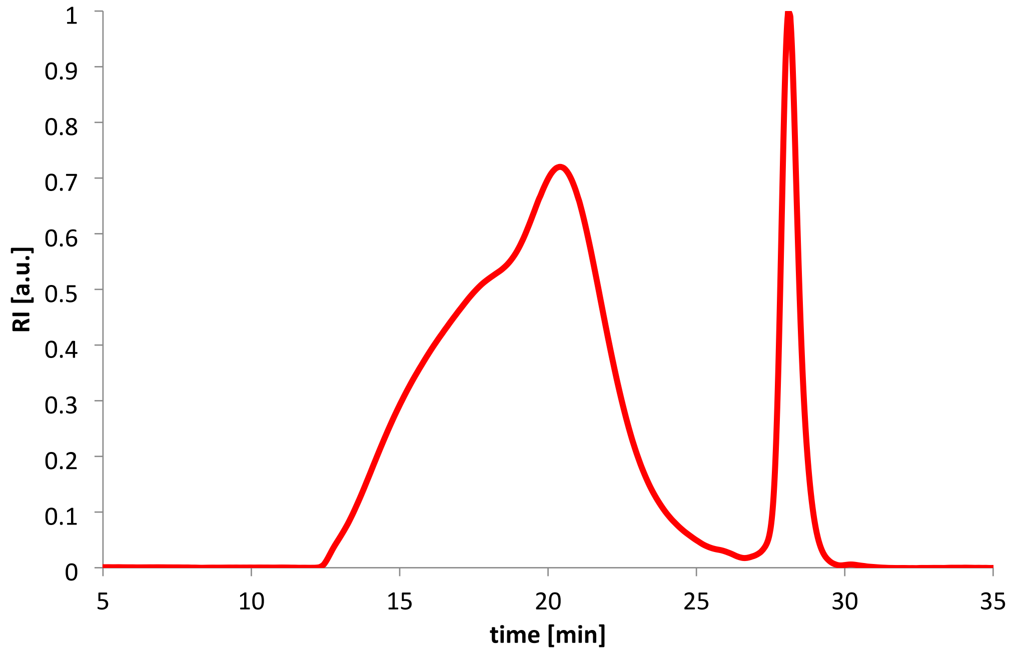

GPC chromatogram of **fC2**, plotting dRI detector data.

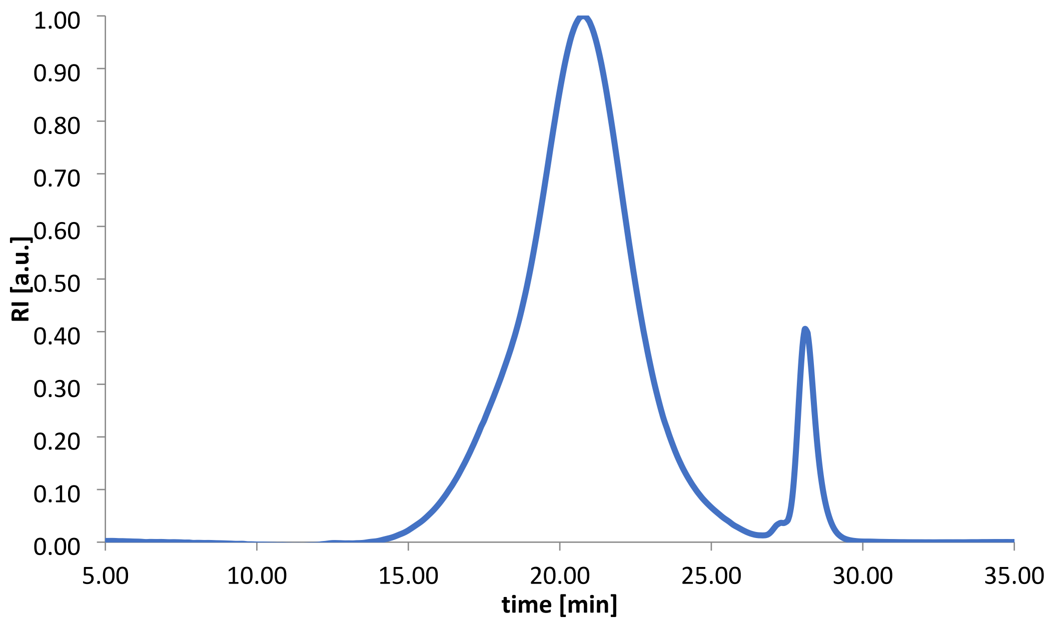

GPC chromatogram of **fC3**, plotting dRI detector data.

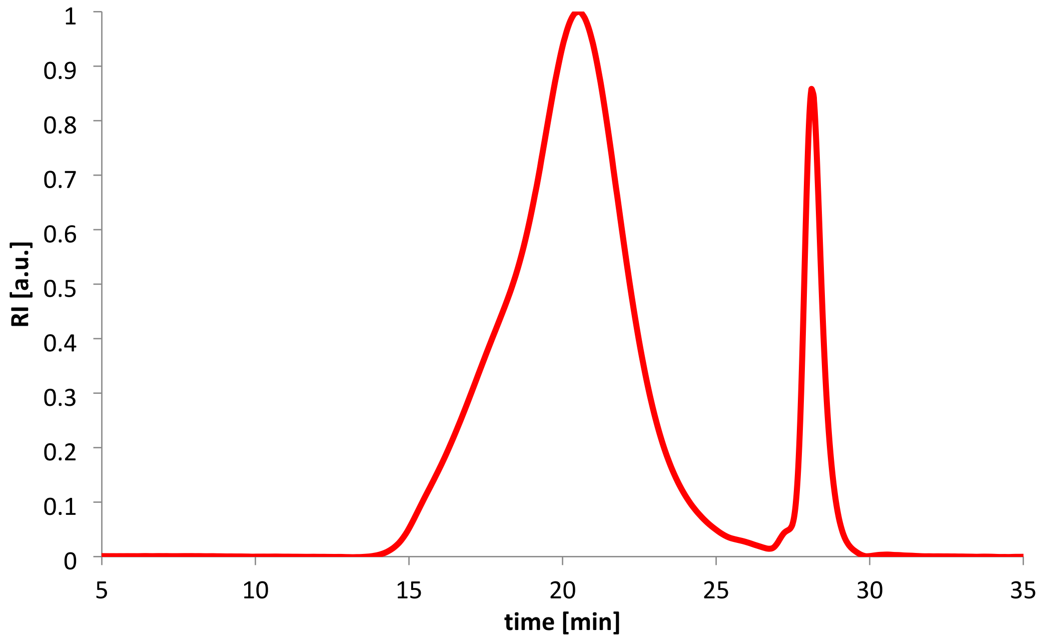

GPC chromatogram of **C4**, plotting dRI detector data.

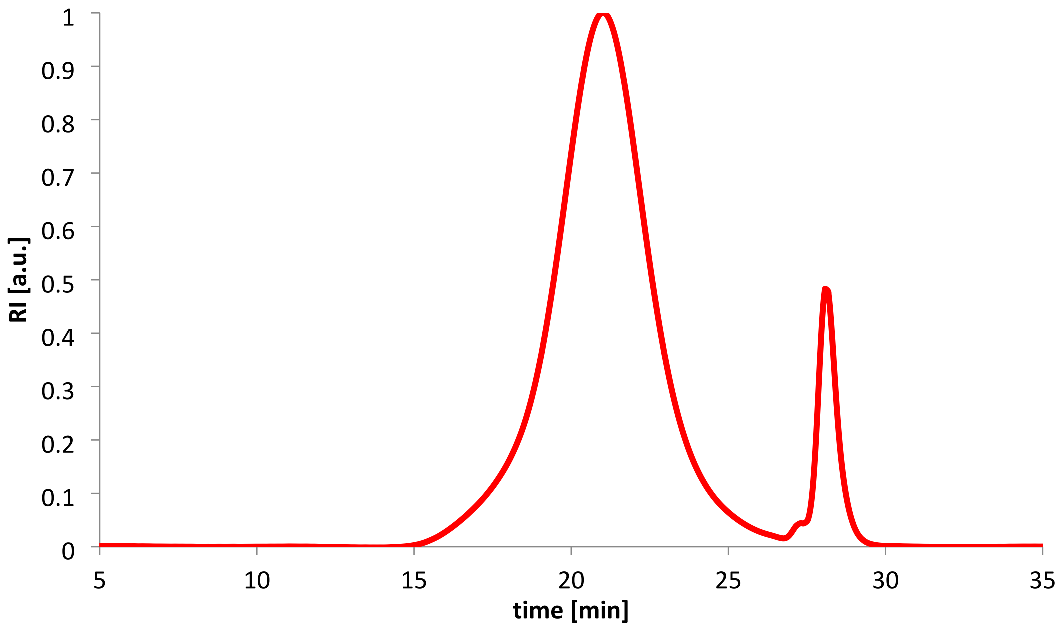

GPC chromatogram of **C5**, plotting dRI detector data.

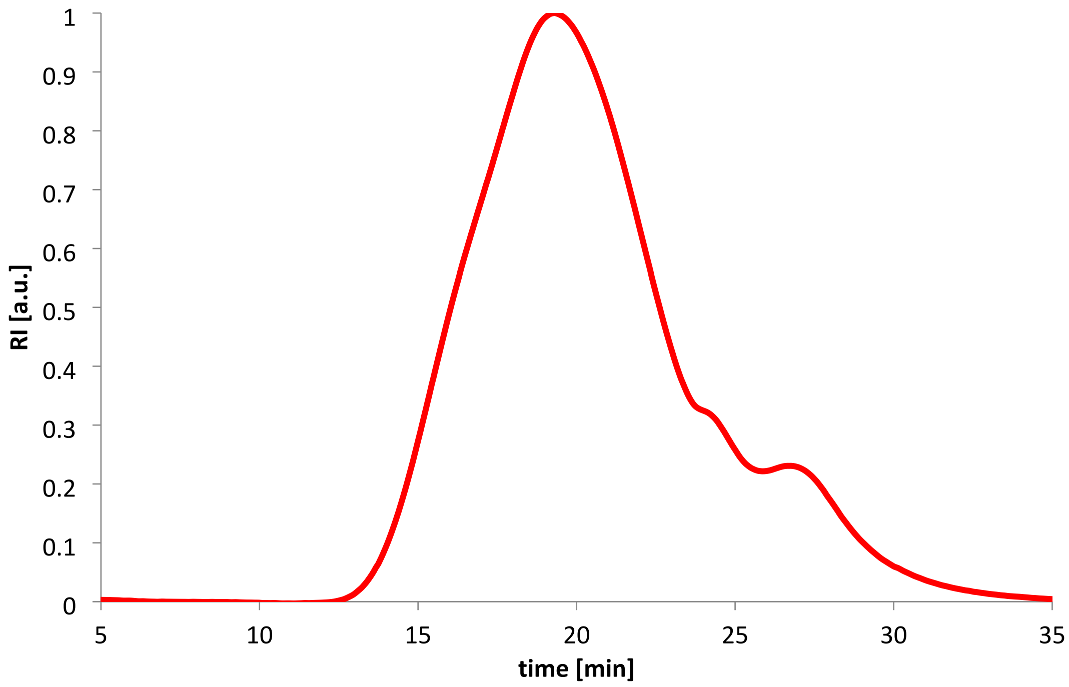

GPC chromatogram of **C6**, plotting dRI detector data.

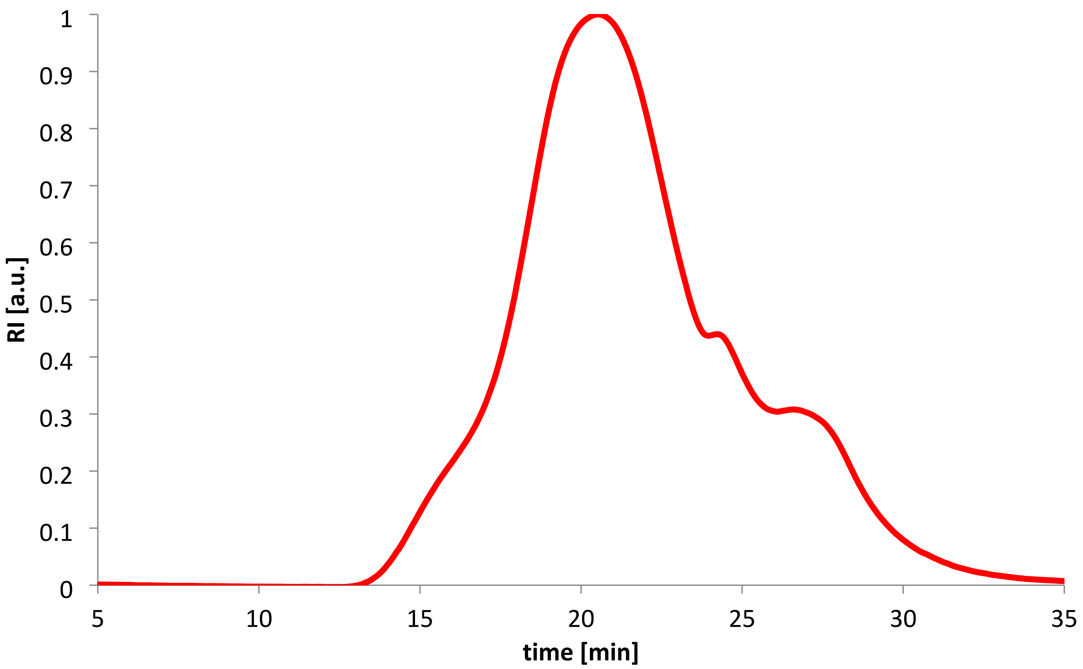

GPC chromatogram of **C7**, plotting dRI detector data.

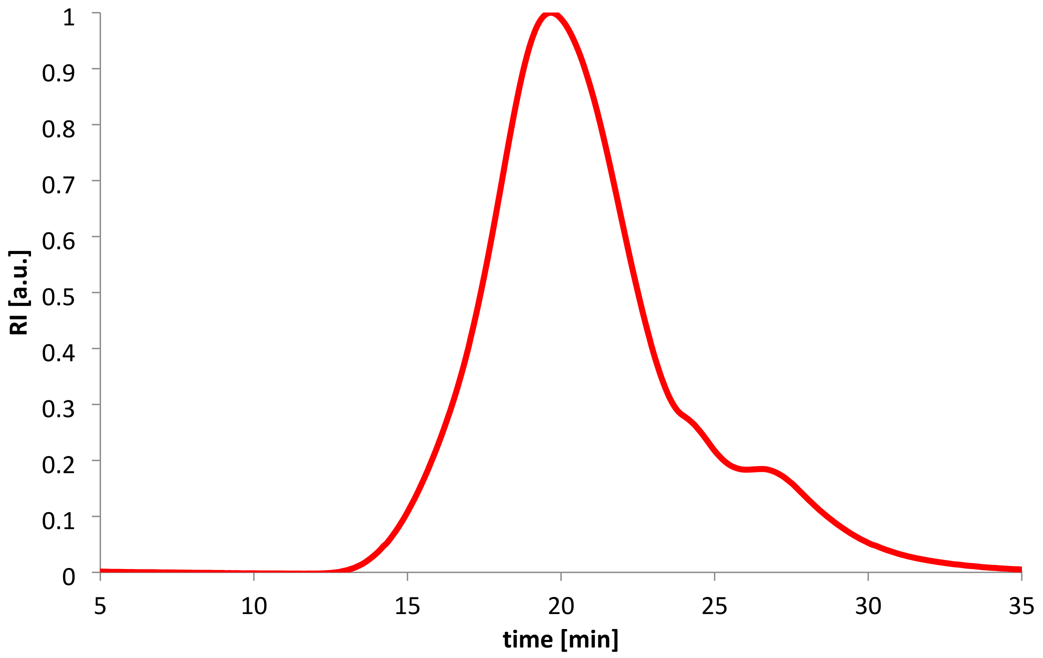

GPC chromatogram of **C8**, plotting dRI detector data.

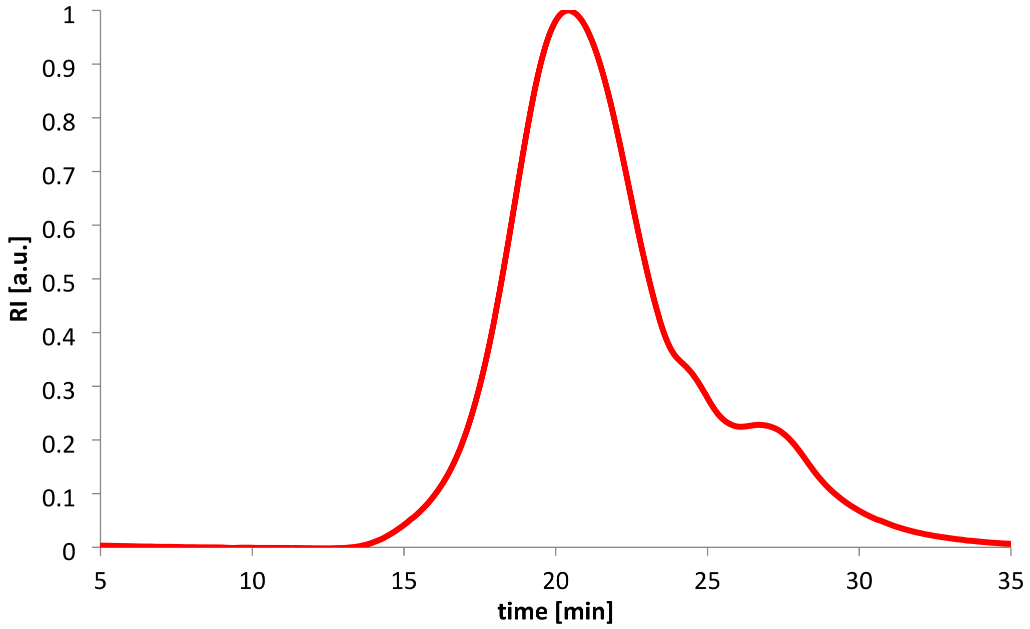

GPC chromatogram of **C9**, plotting dRI detector data.

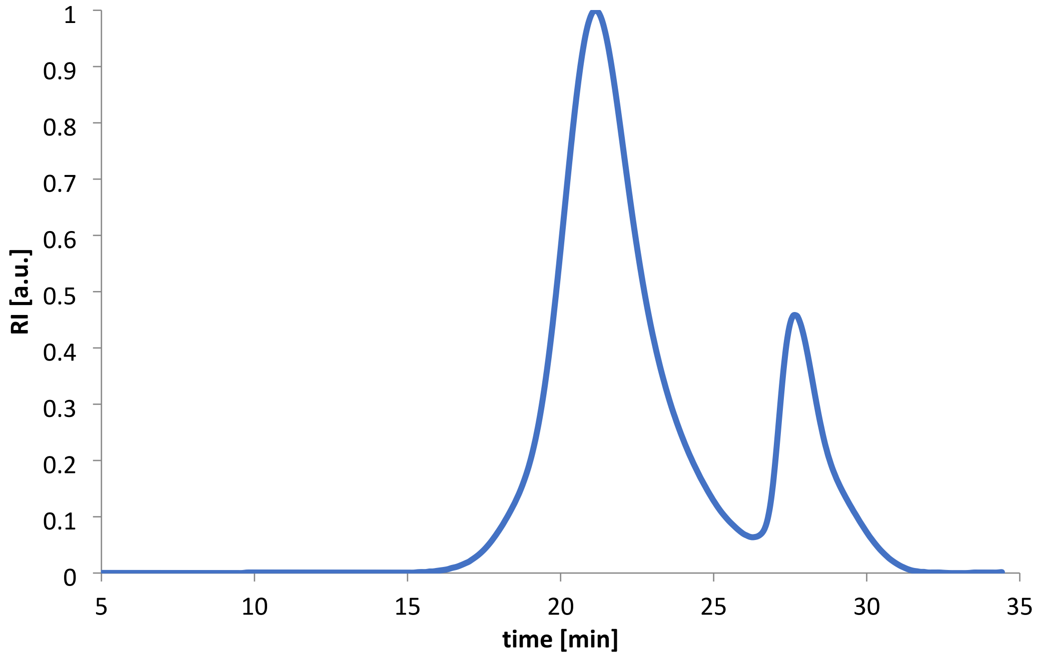

GPC chromatogram of **C10**, plotting dRI detector data.

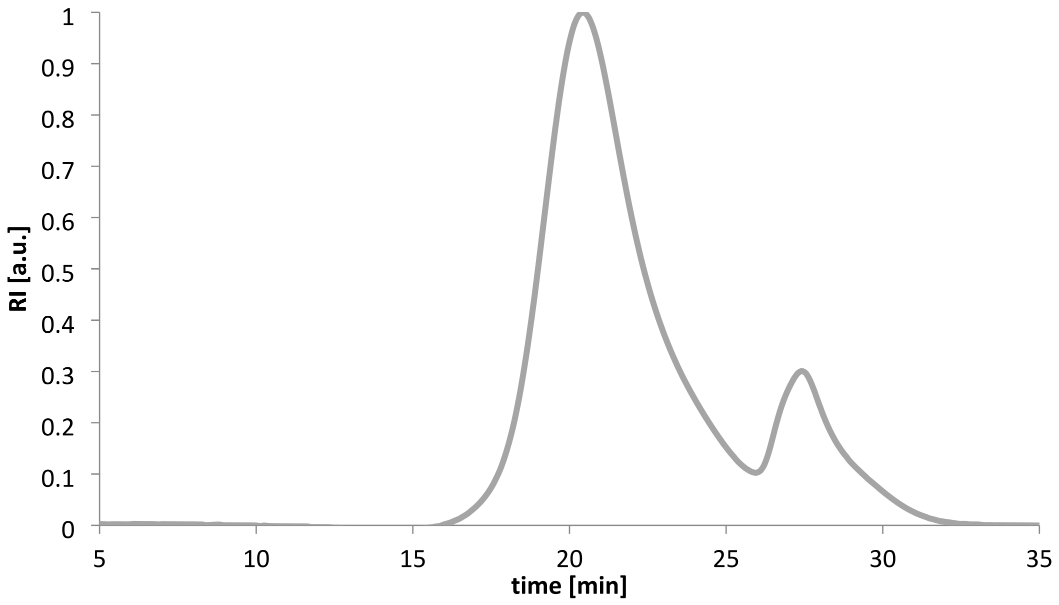

GPC chromatogram of **C11**, plotting dRI detector data.

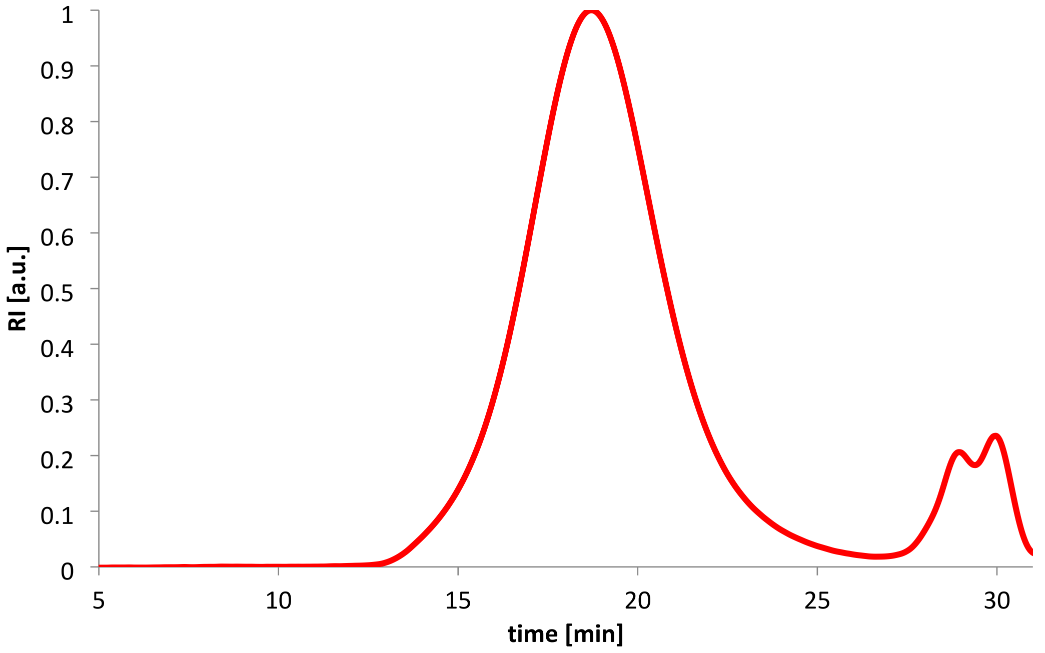

GPC chromatogram of **C12**, plotting dRI detector data.
